# Evaluation of Halogenopyridinium Cations as Halogen Bond Donors

**DOI:** 10.1021/acs.cgd.1c00805

**Published:** 2021-11-08

**Authors:** Luka Fotović, Nikola Bedeković, Vladimir Stilinović

**Affiliations:** Department of Chemistry, Faculty of Science, University of Zagreb, Horvatovac 102a, 10000 Zagreb, Croatia

## Abstract

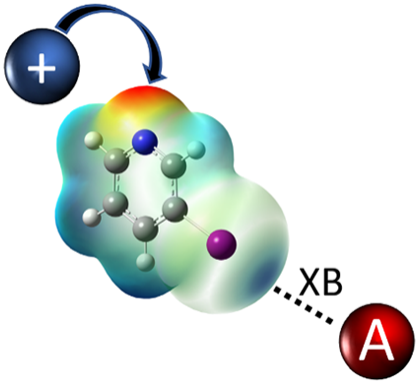

We have performed a database survey and a structural and computational study of the potential and the limitations of halogenopyridinium cations as halogen bond donors. The database survey demonstrated that adding a positive charge on a halogenopyridine ring increases the probability that the halogen atom will participate in a halogen bond, although for chloropyridines it remains below 60%. Crystal structures of both protonated and *N*-methylated monohalogenated pyridinium cations revealed that the iodo- and bromopyridinium cations always form halogen-bonding contacts with the iodide anions shorter than the sum of the vdW radii, while chloropyridinium cations mostly participate in longer contacts or fail to form halogen bonds. Although a DFT study of the electrostatic potential has shown that both protonation and *N*-methylation of halogenopyridines leads to a considerable increase in the ESP of the halogen σ-hole, it is generally not the most positive site on the cation, allowing for alternate binding sites.

## Abstract 2

Both protonation and *N*-methylation of halogenopyridines lead to a considerable increase in the ESP of the halogen σ-hole. This makes the cations better halogen bond donors, comparable to perfluorinated halobenzenes. However, chlorine atoms, in spite of a large positive σ-hole ESP, remain relatively poor halogen-bond donors.

## Introduction

The crucial feature that enables halogen atoms to act as Lewis acids in order to form halogen bonds^1−6^ is the presence of an area of depleted electron density (σ-hole)^7,8^ in the continuation of the covalent bond. The energy of a halogen bond formed between donors with a given acceptor increases with the electrostatic potential of the σ-hole on the donor halogen atom. Therefore, the best halogen-bond donors are those that comprise polarizable halogen atoms (iodine and bromine) bonded to a molecule that can exhibit an electron-withdrawing effect on the halogen atom—thus increasing the electrostatic potential of the σ-hole on the halogen atom and consequently the potential of the halogen atom to form a halogen bond with a Lewis base.^9−12^

To date, most commonly used halogen bond donors have been perfluorinated iodo- or/and bromohydrocarbons,^13−22^ where the electronegative fluorine substituents on the hydrocarbon skeleton act as electron-withdrawing fragments. Other electron-withdrawing substituents, such as cyano and nitro groups, can also be employed.^23−27^ Halogenoethyne derivatives were also successfully used as halogen bond donors, as there the large positive potential of the σ-hole is ensured by the electron-withdrawing properties of the C–C triple bond.^28−31^ Alternatively, halogen atoms can be directly bonded to a (more electronegative) heteroatom, such as nitrogen in (*N*-halogeno)imides—a strategy that has yielded some of the strongest organic halogen bond donors studied to date.^32−39^ Another approach is using hypervalent halogens such as in iodine(I) and iodine(III) compounds. This can lead to much higher positive charges on the halogen atoms, making them very strong halogen bond donors.^40,41^

The aforementioned principles have mainly been employed for the design of neutral halogen bond donors. There is, however, another approach for ensuring a large positive electrostatic potential on a halogen atom: making the halogen atom a part of a positively charged species. The most widely studied group of compounds has been based on halogenated aromatic (primary) amines,^42−45^ as well as halogenated N-heterocycles (such as halogenopyridines and halogenoimidazoles) and their derivatives.^46−77^ These compounds are a logical starting point for the synthesis of cationic halogen-bond donors: on the one hand, their structural rigidity allows for simple control of the geometry of the formed bonds, and on the other hand, they can easily be transformed into cations, either by protonation or by alkylation of the nitrogen atom.

Over the last 20 years both protonated and methylated halogenopyridines have been extensively studied as halogen-bond donors. They have been found to form halogen bonds with organic (such as saccharinate,^46^ bromanilate^47^) and inorganic anions (such as halogenides,^48−53^ halogenometalates,^54−64^ cyanometalates,^65−67^ etc.) Molecules containing iodopyridinium groups have also been designed to act as anion receptors^68−72^ and even as halogen-bond donors in catalysts of halogenide abstraction.^73^

*N*-Alkylated halogenopyridinium cations have also found their place as counterions in Ni(dmit)_2_ (dmit = 1,3-dithiol-2-thione-4,5-dithiolate) salts, which have been synthesized and investigated as supramolecular conductors—because of the possibility of participating in multiple hydrogen and halogen bonds, cations of this type were used to control the conductivity and magnetic properties of these materials.^74−77^

Similarly to neutral halogen-bond donors, cationic halogen-bond donors decrease in strength from iodo to chloro derivatives.^50,52,70,78,79^ Indeed, while iodopyridinium cations form halogen bonds rather predictably, chloropyridinium cations often fail to form halogen bonds, even though accessible acceptors are present in the crystal structure. Willett and co-workers have shown that in the structures of halogenopiridinium tetrahalocuprate(II) salts the C–Br···X^–^ halogen bonds are relatively shorter than the analogous C–Cl···X^–^ halogen bonds, which sometimes are even not present.^55^ Likewise, our previous work on halogenopyridinium hexacyanoferrates has shown that in structures containing a chloropyridinium cation a halogen bond with chlorine as the donor was not present, despite the presence of multiple potential acceptor sites.^67^

In this paper we have endeavored to investigate more closely both the potential and the limitations of halogenopyridinium cations as halogen-bond donors. For this purpose, we have selected monohalogenated pyridine derivatives (*ortho*, *meta*, and *para*; chloro, bromo, and iodo), as both protonated and *N*-methylated pyridinium cations. For the study of their halogen-bonding potential in the solid state, we have opted for the iodide salts—the iodide anion has been shown to be the most reliable halogen bond acceptor among halogenides.^50^ The structures of (protonated) halogenopyridinium iodides have been previously reported^50,51^ and were included in the analysis as such, while the *N*-methylated halogenopyridinium iodides were synthesized and structurally characterized. Along with the comparative study of halogen bond geometries in the crystals of the two series of crystalline solids, we have performed DFT calculations in order to better understand the fundamental reasons for the observed behavior of halogenopyridinium cations.

## Results and Discussion

To ascertain whether there is a statistical trend toward an increase in the halogen-bond probability with addition of a positive charge on a halogenopyridine ring, we have performed a Cambridge Structural Database^80^ (version 5.42 Update 3 (May 2021) CSD database using ConQuest Version 2020.3.0) survey of structures that comprise a halogen substituent on an aromatic nitrogen heterocycle. This has yielded a total of 3112 data sets, the majority of which corresponded to neutral molecules, and 655 to cations derived from them. Of these, 524 were structures containing *N*-protonated cations and only 131 structures with *N*-alkylated cations. For neutral halogenoheterocycles it has been found that in ca. 33% of the structures the halogen atom is in close contact (less than the sum of van der Waals radii) with a potential halogen-bond acceptor (either a nitrogen or oxygen atom or a halogenide anion), indicating the presence of a halogen bond. Halogen-bonding contacts were expectedly found to be the least frequent in chloroheterocycles (27%) followed by bromoheterocycles (39%) and finally iodoheterocycles, where they were found with the greatest frequency (70%). Adding a positive charge (either by protonation or by alkylation of the heterocyclic nitrogen atom) led to a definite increase in the frequencies of halogen bonding, which increased to 56% for cations derived from chloroheterocycles, 82% for bromobromoheterocycles, and 89% for iodoheterocycles. In order to obtain a more detailed picture, we have also performed a series of searches limited to monohalogenopyridines. This has also shown a clear and drastic increase of incidence of halogen bonding upon addition of a positive charge to the pyridine ring—from ca. 7% to ca. 51% for chloropyridines, from ca. 8% to ca. 87% for bromopyridines, and from ca. 40% to ca. 90% for iodopyridines. The increase appears to be somewhat larger for *o*-halogenopyridines (from ca. 8% to ca. 79%) than for *m*-halogenopyridines (from ca. 4% to ca. 70%), as one might expect on the basis of both the proximity of the halogen to the protonated/alkylated nitrogen atom and the resonance effect. The influence of the resonance should also be significant when the halogen is in the *para* position. Unfortunately, this could not be confirmed on the basis of the CSD data—while the incidence of halogen bonds in cations derived from *p*-halogenopyridines is close to that in *o*-halogenopyridinium cations (81%), the number of structures with neutral *p*-halogenopyridines (only four structures with *p*-iodopyridine) in the CSD was too low to allow for a reasonable estimate of the incidence of halogen bonds.

Overall, the CSD data indicate that halogen atoms on a neutral pyridine ring are quite poor halogen-bond donors (except for iodine), but when the pyridine ring is charged, the incidence of the halogen atom acting as a halogen-bond donor dramatically increases. However, this increase does not necessarily make the halogen atoms on charged pyridine rings optimal halogen bond donors—while iodine atoms on either protonated or *N*-alkylated pyridinium cations act as halogen bond donors in ca. 90% of the cases, chlorine does so in only ca. 40–50% of the structures. For comparison, neutral molecules used as “classical” halogen bond donors generally form halogen bonds with reliability similar to that of iodopyridinium cations: fluorinated iodobenzenes form a halogen bond with a potential halogen-bond acceptor (a nitrogen or oxygen atom or a halogenide anion) in 85% (447 out of 524) of the structures where such acceptors are present, fluorinated bromobenzenes in 87% (45 out of 52) of structures, iodoalkynes in 85% (289 out of 339), bromoalkynes in 69% (34 out of 49), and in virtually all structures of *N*-halogenoimides (37 out of 38 *N*-bromoimides and 32 out of 32 *N*-iodoimides).

As the CSD search provided us with the average behavior of halogenopyridines and corresponding cations with a varied collection of (potential) halogen bond acceptors, we decided to take a closer look at the halogen bonding of protonated and *N*-methylated pyridinium cations with the iodide anion. The iodide was a logical choice, as it is on the one hand a halogenide, a simple spherical anion without steric or other issues that might complicate the overall picture of the supramolecular interactions in the crystal, and also as it was found to form halogen bonds most reliably among the halogenides.^50^ The crystal structures of chloropyridinium and bromopyridinium iodides have been investigated by Awwadi, Willett and co-workers,^51^ while iodopyridinium iodides were reported recently by our group.^50^ All of the crystal structures featured in both studies have been determined at room temperature, allowing for meaningful comparisons among the halogen bond geometries. The bond lengths and angles are given in Table 1.

**Table 1:** Overview of Halogen Bonding in Iodides of Protonated and Methylated Halogenopyridinium Cations (X- Ray Data Measured at Room Temperature)

protonated halogenopyridinium iodide	*d*(XB)/Å	RS^a^/%	*N*-methylated halogenopyridinium iodide	*d*(XB)/Å	RS/%
[**2-ClPy**H]I	3.768	–0.1	[**2-ClPy**Me]I^b^	3.496	6.3
				3.509	5.9
				3.511	5.9
[**2-BrPy**H]I	3.575	5.9			
[**2-IPy**H]I	3.467	12.4	[**2-IPy**Me]I	3.459	12.7
[**3-ClPy**H]I	3.739	–0.2	[**3-ClPy**Me]I	3.774	–1.2
[**3-BrPy**H]I	3.589	6.3	[**3-BrPy**Me]I	3.637	5.0
[**3-IPy**H]I	3.516	11.2	[**3-IPy**Me]I	3.538	10.7
[**4-ClPy**H]I	3.733	–0.1	[**4-ClPy**Me]I	3.587	3.8
[**4-BrPy**H]I	3.648	4.8			
[**4-IPy**H]I	3.532	10.8	[**4-IPy**Me]I	3.552	10.3

a RS(XB) = 100[1 – (*d*(X···I^–^)/(*r*(X) + *r*(I^–^))],where *r*(X) and *r*(I^–^) are the van der Waals radii of the corresponding atoms.

b Measured at 170 K.

**Figure 1:**
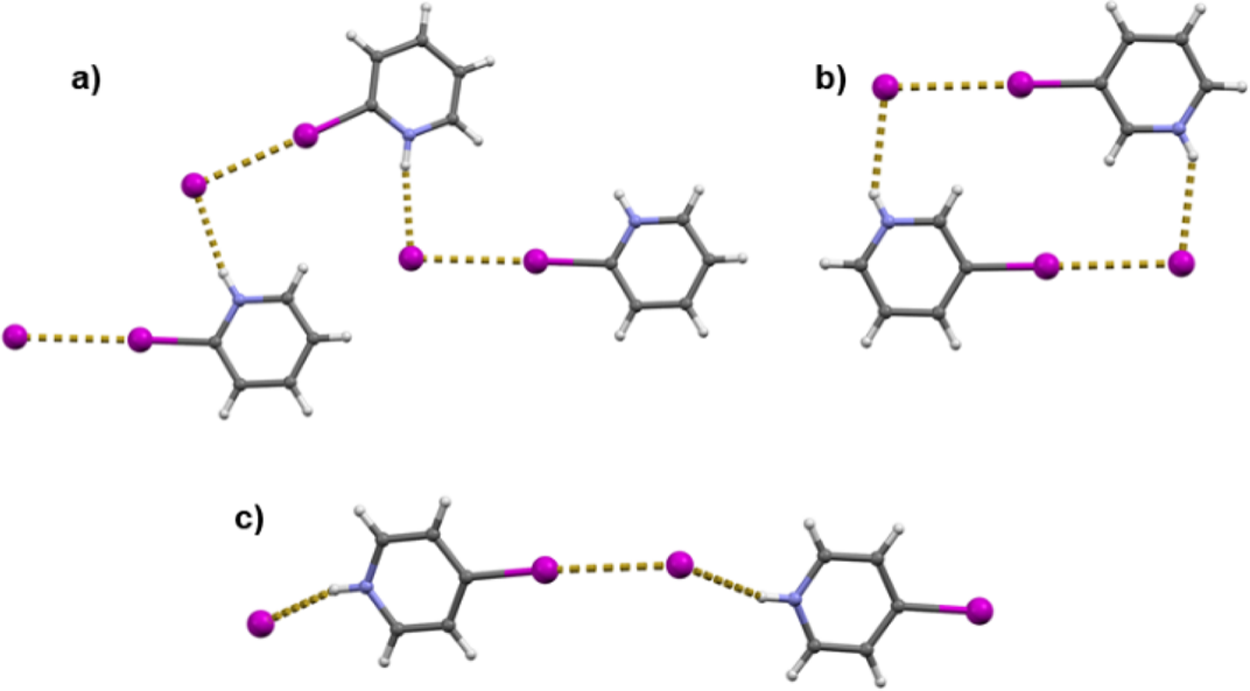
(a) Halogen- and hydrogen-bonded chains in *o*-iodopyridinium iodide. (b) Halogen- and hydrogen-bonded tetramers in *m*-iodopyridinium iodide. (c) Halogen- and hydrogen-bonded chains in *p*-iodopyridinium iodide.^51^ Halogen bond lengths are given in Table 1.

In the structures of all three *o*-halogenopyridinium ([**2-XPy**H]) iodides, halogenopyridinium cations and iodide anions are interconnected in chains trough N–H···I^–^ hydrogen and C–X···I^–^ halogen bonds (Figure 1a). In [**2-ClPy**H]I the C–X···I^–^ halogen bonds are longer than in the bromo and iodo analogues and longer than the sum of van der Waals radii (Table 1). The crystal structures of *m*-halogenopyridinium ([**2-XPy**H]) iodides comprise centrosymmetric cyclical (**3-XPy**H)_2_I_2_ tetramers in which **3-XPy**H^+^ cations and iodide anions are interconnected trough N–H···I^–^ hydrogen and C–X···I^–^ halogen bonds (Figure 1b). The relative shortening of the C–X···I^–^ halogen bonds decreases from ca. 11.2% in the **3-IPy** derivative over ca. 6.3% in the **3-BrPy** derivative to ca. 0.2% in the **3-ClPy** derivative, again with the C–Cl···I^–^ contact being longer than the sum of the van der Waals radii. Also, the halogen bonds in [**3-IPy**H]I and [**3-BrPy**H]I are almost linear (C–X···I^–^ angles above 176°), while in [**3-ClPy**H]I the analogous C–Cl···I^–^ angle is ca. 165°. In the case of *p*-halogenopyridinium (**4-XPy**) iodides, chains are again formed by alternating hydrogen and halogen bonds between alternating **4-XPy**H^+^ cations and iodide anions (Figure 1c). The C–X···I^–^ halogen bonds in the iodide salts of the *para* isomers are somewhat longer than in the *ortho* and *meta* isomers but also follow the same trend in decreasing from ca. 10.8% in [**4-IPy**H]I to ca. 4.8% in [**4-BrPy**H]I. The C–Cl···I^–^ contact in [**4-ClPy**H]I is again longer than the sum of the van der Waals radii by ca. 0.1%. The halogen bond angle is approximately linear only in [**4-IPy**H]I, whereas in both [**4-ClPy**H]I and [**4-BrPy**H]I the angles considerably deviate from linearity (C–X···I^–^ angles of ca. 164°).

In order to observe the halogen-bonding behavior of positively charged halogenopyridines in the absence of a strong N–H···I^–^ hydrogen bond, we decided to synthesize and crystallize an equivalent series of *N*-methylated halogenopyridinium iodides. Although we were unfortunately unable to produce two members of the series (derived from **2-BrPy** and **4-BrPy**; see the Experimental Section for details), the crystal structures of the seven compounds we have obtained were sufficient to accentuate significant differences in the observed trends in protonated halogenopyridinium iodides.

Among the salts of *N*-methylated halogenopyridines there is considerably less structural similarity within the *o*-, *m*-, and *p*- substituted groups. This is to be expected, as in the absence of the directing influence of the hydrogen bond that can combine with the halogen bond, the only interaction of significance is the halogen bond, and the crystal structure will be predominantly determined by assembly of (halogen-bonded) ion pairs by weak interactions.

**Figure 2:**
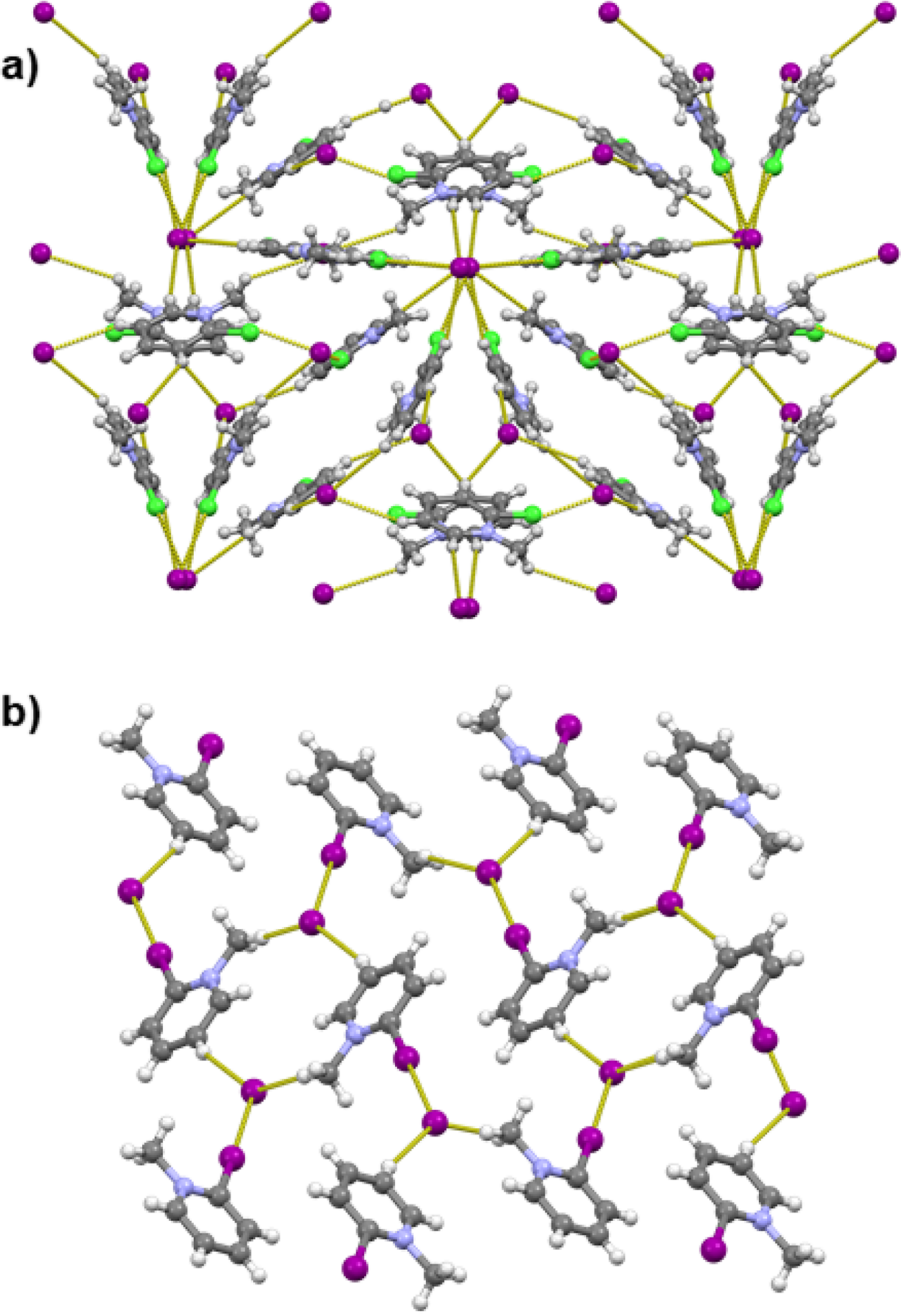
Halogen bonds and short C–H···I^–^ contacts in crystal structures of *N*-methyl-2-halogenopyridinium iodides: (a) the 3D network in [**2-ClPy**Me]I (view along the *c* axis); (b) the 2D network in [**2-IPy**Me]I (view along the *a* axis). Halogen bond lengths are given in Table 1.

The most dissimilar are the structures of the *o*-substituted [**2-ClPy**Me]I and [**2-IPy**Me]I. The structure of [**2-IPy**Me]I consists of the expected assembly of halogen-bonded ion pairs of *o*-iodopyridinium cations and iodide anions trough C–I···I^–^ halogen bonds, which further interconnect via C–H···I^–^ hydrogen bonds into helical chains (Figure 2a). Conversely, the structure of [**2-ClPy**Me]I is a complex 3D network assembled through C–Cl···I^–^ and C–H···I^–^ contacts with four *o*-chloropyridinium cations and four iodide anions in the asymmetric unit (Figure 2a). These do not form clear halogen-bonded ion pairs as was the case in [**2-IPy**Me]I—only two iodide anions and three cations form halogen bonds (two cations bind to the same iodide), while the remaining cation and anions participate only in C–H···I^–^ hydrogen bonds. Of the four cations, one is disordered over a crystallographic inversion center; the disordered cations form C–Cl···I^–^ halogen bonds along the crystallographic *b* axis and C–H···I^–^ hydrogen bonds along the crystallographic *c* axis with independent iodide anions (both somewhat disordered over inversion centers), thus forming disordered layers perpendicular to the crystallographic *a* axis.

**Figure 3:**
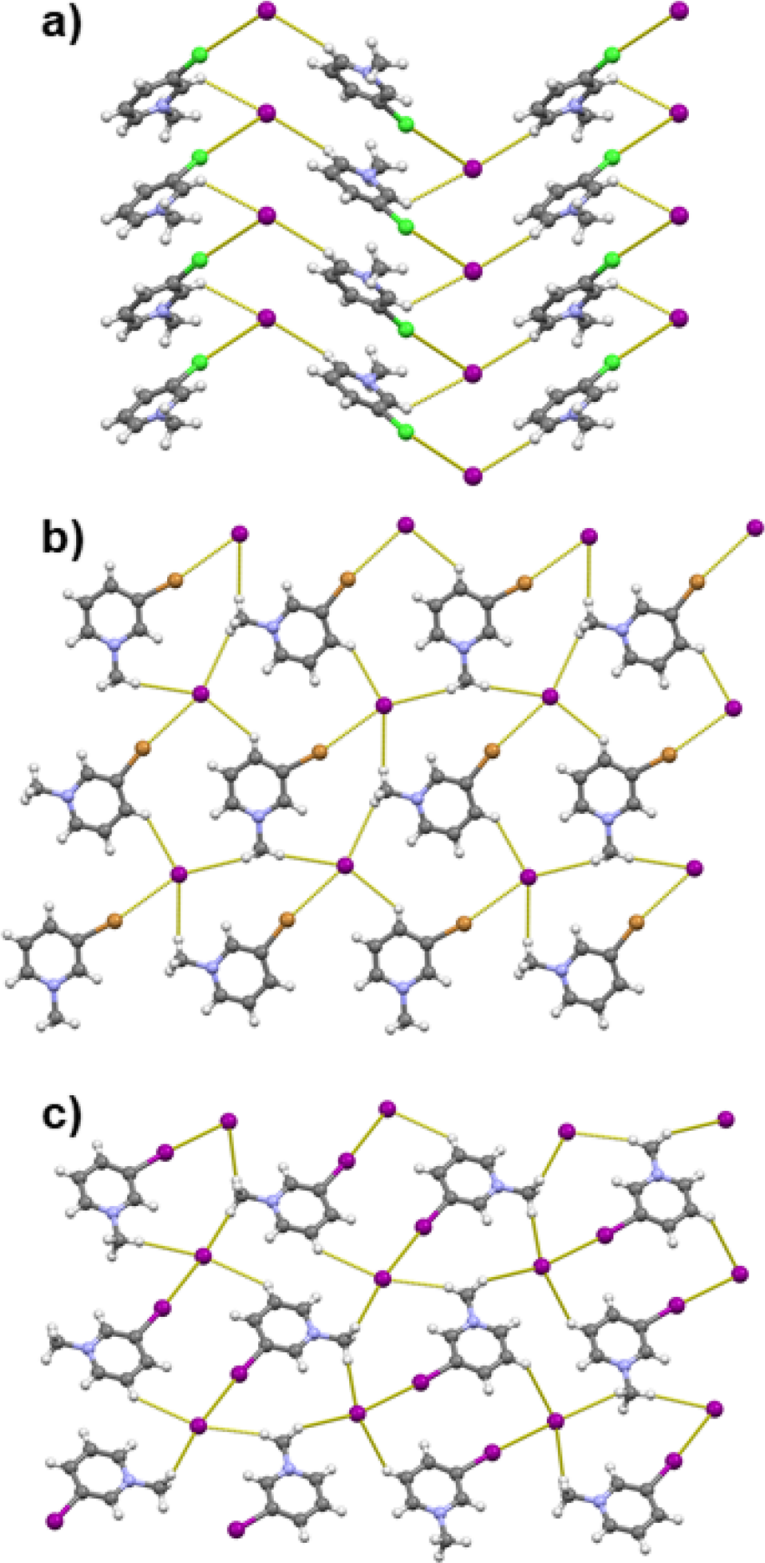
Halogen- and hydrogen (C–H···I^–^)-bonded 2D networks in crystal structures of *N*-methyl-3-halogenopyridinium iodides: (a) [**3-ClPy**Me]I (view along the *a* axis); (b) [**3-BrPy**Me]I (view along the *c* axis); (c) [**3-IPy**Me]I (view along the *a* axis). Halogen bond lengths are given in Table 1.

In the structures of iodides derived from *meta*-substituted pyridines ([**3-IPy**Me]I, [**3-BrPy**Me], and [**3-ClPy**Me]I) the halogenopyridinium cations and iodide anions are connected into chains via C–X···I^–^ halogen and C–H···I^–^ hydrogen bonds. [**3-BrPy**Me]I and [**3-IPy**Me]I are quite similar in structural arrangement, although they are not isostructural, as they differ in the space group symmetry. In both structures, the hydrogen- and halogen-bonded chains are interconnected into planar layers trough additional C–H···I^–^ hydrogen-bonding contacts (Figure 3b,c). The C–I···I^–^ and C–Br···I^–^ halogen bonds are shorter than the corresponding sum of van der Waals radii by ca. 11% and 5%, respectively. In [**3-ClPy**Me]I the chains of ion pairs are also interconnected into layers through C–H···I^–^ hydrogen bonds (Figure 3a); however, the layers formed here are not planar but corrugated. Although it is longer (ca. 1%) than the sum of the corresponding van der Waals radii, the C–Cl···X^–^ contact can still have a significant effect on the structural arrangement,^81^ as appears to be the case in the structure of [**3-ClPy**Me]I.

**Figure 4:**
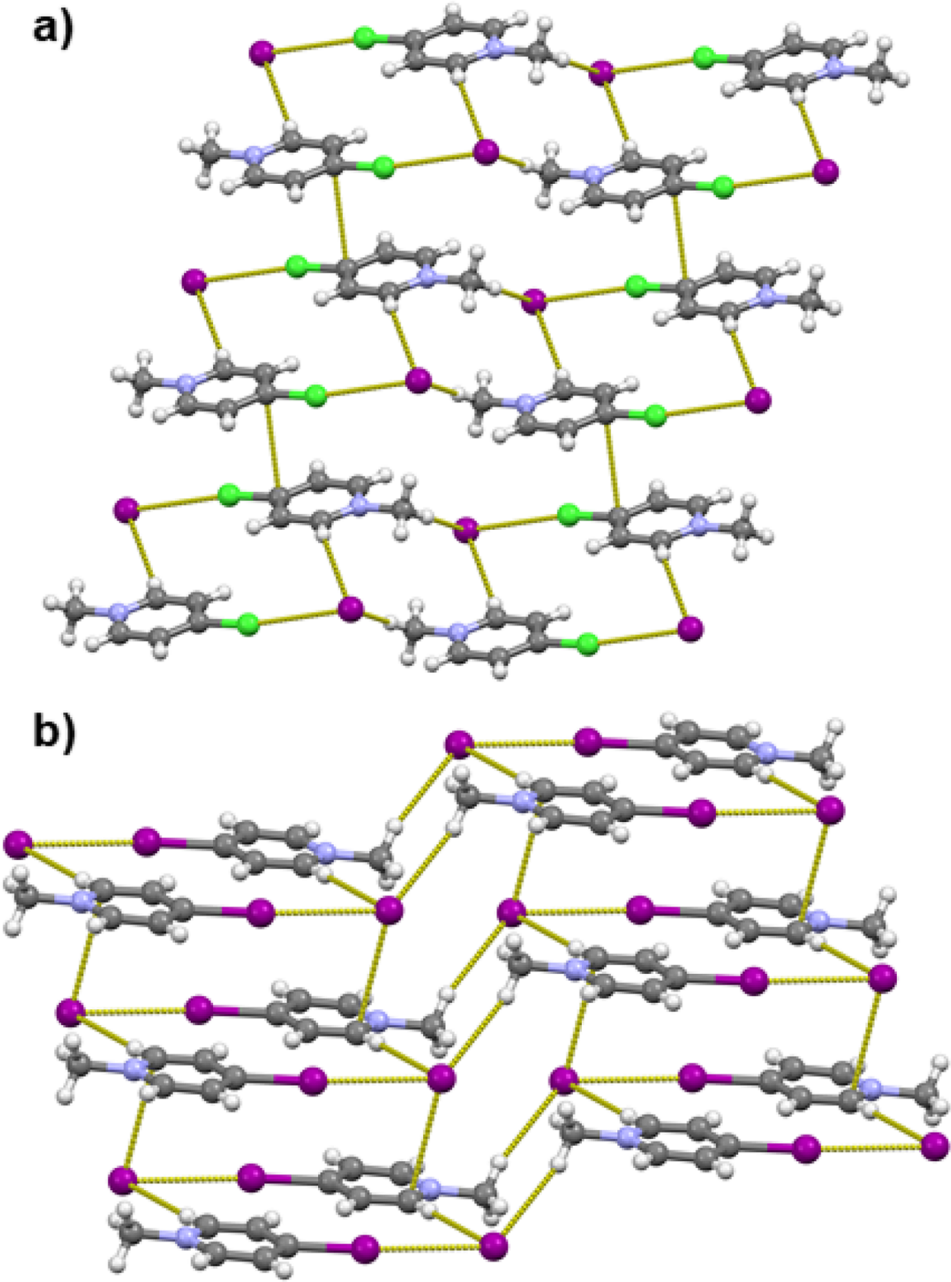
Halogen- and hydrogen (C–H···I^–^)-bonded tetramers of *N*-methyl-4-halogenopyridinium iodides interconnected by (a) π-stacking in [4**-ClPy**Me]I and (b) anion−π contacts in [**4-IPy**Me]I. Halogen bond lengths are given in Table 1.

In the case of the structures derived from *para*-substituted pyridines, [**4-IPy**Me]I and [**4-ClPy**Me]I, the cations and the anions are connected via C–X···I^–^ halogen and C–H···I^–^ hydrogen bonds into centrosymmetric cyclical ([**4-XPy**Me]I)_2_ tetramers (Figure 4). In both structures the C–I···X^–^ contacts are shorter than the sum of van der Waals radii and are quite linear (ca. 10% and 4% with a ∠(C–I···I^–^) angle of ca. 172° and a ∠(C–Cl···I^–^) angle of ca. 170°, respectively). The C–H···I^–^ hydrogen bond is achieved in both cases through equivalent hydrogen atoms (*ortho* relative to the methylated nitrogen), but the geometry of the C–H···I^–^ hydrogen-bonded tetramers is quite different. In the case of [**4-IPy**Me]I the hydrogen bond is almost linear (∠(C–H···I^–^) angle of ca. 178°) and the two pyridinium rings within the tetramer are almost perfectly coplanar (the mean planes of the cations are offset by a mere 0.12 Å). Conversely, in [**4-ClPy**Me]I the ∠(C–H···I^–^) angle is significantly lower (ca. 152°) and the mean planes of the pyridinium cations are offset by ca. 2.18 Å.

The lengths of halogen bonds formed by the *N*-methylated iodopyridinium cations are in most structures comparable to those formed by their respective protonated analogues, the differences between the relative shortenings of any two analogues generally being less than 0.5% (Table 1). However, there is a considerable difference between protonated and methylated chloropyridinium iodides, the C–Cl···I^–^ contact in [**4-ClPy**Me]I being 3.8% shorter and in [**4-ClPy**H]I 0.1% longer than the sum of the van der Waals radii. This implies that the presence of a strong N–H···I^–^ hydrogen bond has a greater effect on the elongation of the (weaker) C–Cl···I^–^ halogen bond, while its effect on the (stronger) C–I···I^–^ halogen bond is negligible. The comparison of halogen bond lengths in [**2-ClPy**Me]I and [**2-ClPy**H]I would also seem to support this conclusion on first glance. However, as the two data sets have not been measured at the same temperature ([**2-ClPy**Me]I could not be measured at room temperature due to the instability of the crystals), the even shorter C–Cl···I^–^ contacts (5.9–6.3% shorter than the sum of the van der Waals radii) in [**2-ClPy**Me]I are at least partially due to the lower temperature (170 K) at which the crystal was measured and therefore cannot be compared to the rest of the halogen bonds in this study, which were all measured at room temperature.

On comparison of halogen bonds between the “classical” (neutral) halogen bond donors and the iodide anion as an acceptor, it can be seen that there is only a slight difference in halogen bond length between halogen bonds formed with iodopyridinium cations and neutral perfluorinated iodobenzenes as halogen bond donors. The former form halogen bonds which are on average 11.4% shorter than the sum of the van der Waals radii, while the latter form halogen bonds which are on average 9.9% shorter than the sum of the van der Waals radii (based on 19 crystal structures of cocrystals of iodides with neutral perflorinated iodobenzenes measured at room temperature deposited in the CSD). On comparison to an even stronger neutral halogen bond donor such as *N*-iodosuccinimide, the iodopyridinium cations are clearly poorer halogen-bond donors, as in the only crystal structure reported to date of a cocrystal of *N*-iodosuccinimide and an iodide^82^ the halogen bond is ca. 21% shorter than the corresponding sum of the van der Waals radii. Comparing the lengths of halogen bonds with N and O acceptors where the donors are cations derived from halogenoheterocycles with those formed by different types of neutral donors (Table 2) shows that with nitrogen acceptors all three groups of cationic halogen-bond donors tend to form longer bonds in comparison to some of the most commonly used neutral donors (fluorinated halogenobenzenes, halogenoalkynes, and halogenoimides). Only in the case of halogen bonds with oxygen acceptors do the lengths of halogen bonds formed by bromo- and iodo-heterocyclic cations approach the mean values for halogen bonds formed by their fluorinated halogenobenzene counterparts.

**Table 2:** Relative Shortenings (Mean Values in Percent Based on the CSD Data for Structures Measured at Room Temperature) for Halogen Bonds with N and O Acceptors

	halogen bond acceptor	
halogen bond donor	N	O
chloro-heterocyclic cation	1.2	5.5
bromo-heterocyclic cation	6.5	6.2
iodo-heterocyclic cation	7.3	10.9
fluorinated iodobenzenes	15.3	13.3
fluorinated bromobenzenes	12.0	5.8
iodoalkynes	23.0	16.5
bromoalkynes	16.5	9.6
*N*-bromoimides	31.4	19.2
*N*-iodoimides	29.9	26.3

As was mentioned earlier, the C–I···I^–^ halogen bonds in both protonated and *N*-methylated iodopyridinium iodides display the largest relative shortening (RS) in comparison to the corresponding van der Waals radii (generally double that for C–Br···I^–^ halogen bonds). In comparison to the hydrogen bonds present in all three structures with protonated cations, C–I···I^–^ halogen bonds have somewhat smaller RS values than the hydrogen bonds (19.3% for N–H···I^–^ in **[2-IPyH]I**, 11.5% in **[3-IPyH]I**, and 14.5% in **[4-IPyH]I**), except in [**3-IPy**H]I, where they are practically equivalent. As expected, aromatic hydrogen atoms in [**3-IPy**Me]I and [**4-IPy**Me]I are again involved in C–H···I^–^ hydrogen-bonding contacts shorter than the sum of the van der Waals radii (with **3-IPy** hydrogen atoms in the *ortho* and *para* positions to the nitrogen atom by 1% and 3%, respectively, and with **4-IPy** hydrogen atoms in both *ortho* positions by 3% and 1%). In the methylated series additional hydrogen bonds have been formed between methyl hydrogen atoms and the iodide (with **3-IPy** methyl hydrogen atoms by 5% and with **4-IPy** methyl hydrogen and aromatic hydrogen atom in a *para* position longer than sum of the van der Waals radii by 1%). Interestingly, in [**4-IPy**Me]I the iodide anion also participates in a short contact with an aryl carbon atom of a [**4-IPy**Me]^+^ cation. The carbon atom is in the *ortho* position relative to the methylated nitrogen, and the iodide approaches it orthogonally to the plane of the ring. This anion···π contact (with a corresponding RS value of ca. 1%)^83,84^ can therefore be classified as a tetrel π-hole interaction between the iodide and a positive region on the carbon atom perpendicular to the ring plane. Similar anion···π contacts have also been found in crystal structures of several *N*-alkyl-3-halogenopyridinium halogenides.^85^

It can be seen that, although in all of the studied structures the C–X···I^–^ halogen bonding contacts are present, only among the *N*-methyl-3-bromopyridinium and *N*-methyl-iodopyridinium iodides is this halogen bond clearly the dominant interaction in the crystal structures. The cations interact with the iodides not only through the halogen atom (and the N–H group in the protonated pyridine series) but also through fairly short C–H···I^–^ hydrogen-bonding contacts and even C···I^–^ π-hole tetrel bonding contacts. Indeed, in chloropyridinium iodides these “weak” interactions seem to be dominant. The reasons for this phenomenon can be made clear by a detailed study of the electrostatic potential of the potential donor and acceptor sites on both protonated and *N*-methylated pyridinum cations.

As expected, both protonation and *N*-methylation of halogenopyridines lead to an increase in the ESP of the halogen atom σ-hole (*V*_max_(X)). This is most pronounced in *o*-halogenopyridines where the *V*_max_(X) value increases by ca. 400–420 kJ mol^–1^ e^–1^, while in *m*- and *p*-halogenopyridines it increases by ca. 320–350 kJ mol^–1^ e^–1^. Protonation leads to somewhat larger increase of *V*_max_(X) (10–20 kJ mol^–1^ e^–1^) on the halogen atom in comparison to *N*-methylation, as can be expected since in the protonated pyridinium cation there are fewer atoms on which the positive charge is distributed.

Along with the increase in the *V*_max_(X) value, adding a positive charge on the halogenopyridine ring also leads to a change in the polarization of the halogen atom. A convenient measure for the polarization of the halogen atom is the difference between the *V*_max_(X) value and the ESP of the halogen atom perpendicular to it (*V*_min_(X)) (see Tables S4–S9 in the Supporting Information). The *V*_max_(X) – *V*_min_(X) difference (Δ*V*(X)) was generally found to not be considerably affected by the position of the halogen atom and was found in neutral halogenopyridines to be on the average 80 ± 2 kJ mol^–1^ e^–1^ for chlorine, 122 ± 2 kJ mol^–1^ e^–1^, for bromine and 151 ± 3 kJ mol^–1^***e***^–1^ for iodine. In the cations, both *V*_max_(X) and *V*_min_(X) are considerably more positive. The Δ*V*(X) value is again barely affected by the position of the halogen atom as well as whether the cation is protonated or *N*-methylated and depends primarily on the halogen atom. If one is to compare the Δ*V*(X) in neutral halogenopyridines to those in the cations, one can see that in the case of all three chloropyridines the Δ*V*(X) value in the cations is reduced on average to 59 ± 4 kJ mol^–1^ e^–1^ and for bromopyridines it remains almost unchanged (122 ± 7 kJ mol^–1^ e^–1^), while for iodopyridines the Δ*V*(X) value in the cations increases to 164 ± 6 kJ mol^–1^ e^–1^. It therefore follows that the polarization of chlorine is reduced by the presence of the positive charge of the pyridine ring and the polarization of bromine is not affected, while the iodine becomes more polarized when pyridine is protonated or methylated (note that in all cases the *V*_min_(X) value changes sign—it is negative in all neutral halogenopyridines and positive in the corresponding cations).

One would expect that the dramatic increase in the *V*_max_(X) value should also lead to an equally dramatic increase in the potential of the halogenopyridinium cations as halogen-bond donors. However, as evidenced by CSD data, although there is a definite increase in the incidence of a halogen bond in structures comprising halogenopyridinium cations (in comparison to neutral halogenopyridines), it does not appear as dramatic, particularly in the case of chloropyridinium cations. This discrepancy seems particularly surprising if one takes into account that, even in the case of choropyridinium cations, the *V*_max_(X) value on the halogen atom is considerably larger (390–435 kJ mol^–1^ e^–1^) than that on neutral donors (e.g., 175 kJ mol^–1^ e^–1^ in 1,4-diodotetrafluorobenzene), which form halogen bonds with potential donors present in the crystal structures much more reliably in comparison to cations derived from chloroheterocycles (see above).

**Figure 5:**
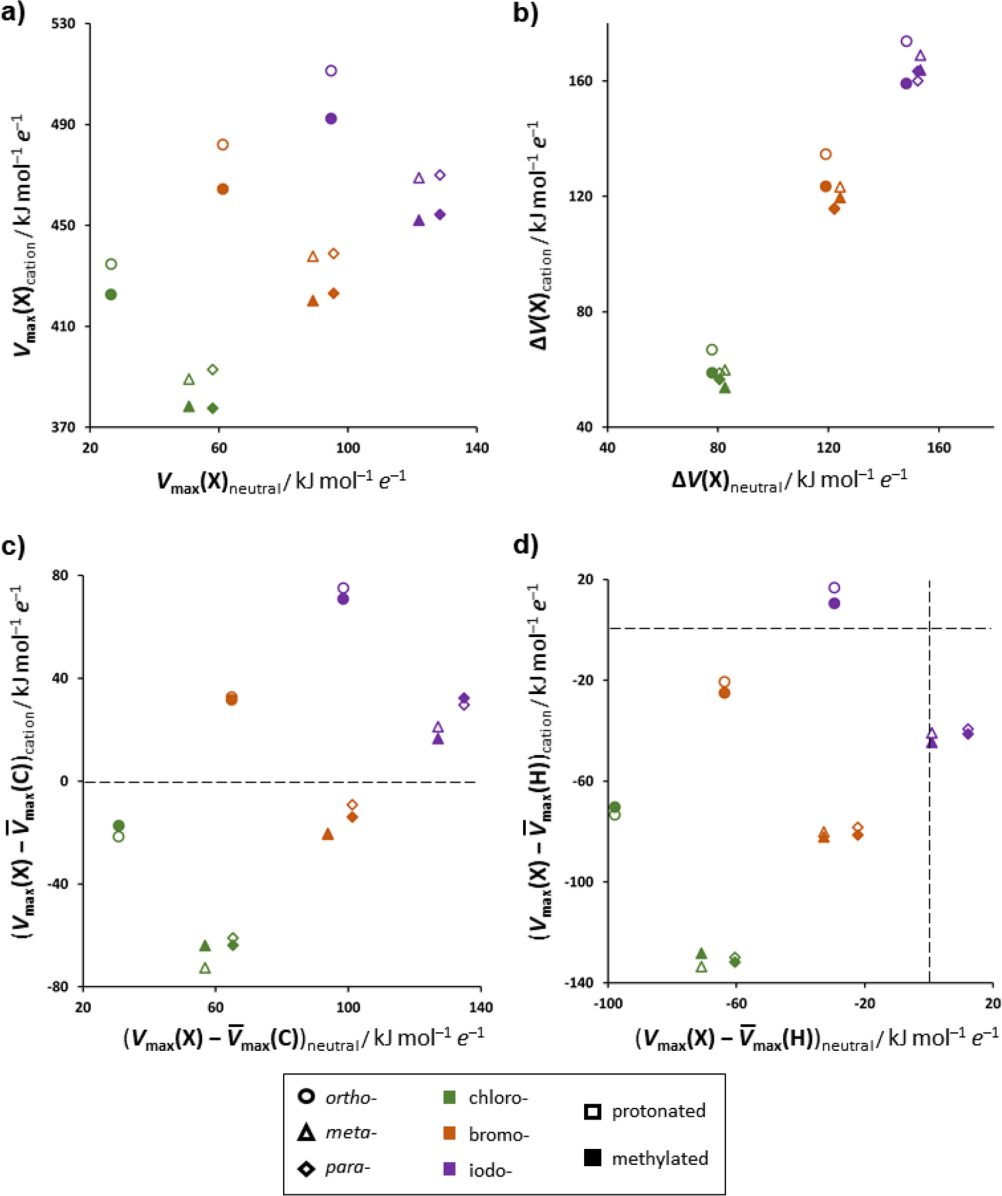
Changes in molecular ESP on halogenopyridines upon protonation and *N*-methylation: (a) plot of the halogen σ-hole ESP (*V*_max_(X)) on cations vs neutral halogenopyridines; (b) plot of the difference between the *V*_max_(X) value and the ESP of the halogen atom perpendicular to it ((Δ*V*(X)) on cations vs neutral halogenopyridines; (c) plot of the difference between the *V*_max_(X) value and the mean ESP on non-hydrogen atoms of the pyridine ring (ESP perpendicular to the plane of the ring) on cations vs neutral halogenopyridines; (d) plot of the difference between the *V*_max_(X) value and the mean ESP on the pyridine ring C–H hydrogen atoms on cations vs neutral halogenopyridines. In (c) and (d) the dashed lines denote where the corresponding values equal zero.

As a probable reason for this, we propose the distribution of the positive charge throughout the halogenopyridinium cation. Adding a proton or a methyl group on the pyridine nitrogen does not affect just the halogen atom—the positive charge is distributed on the entire molecule. To test how this would affect the halogen-bonding proclivity of the halogenopyridinium cation, we set out to see how the *V*_max_(X) value compares to the ESP of the remainder of the molecule in the case of the neutral halogenopyridines as well as halogenopyridinium cations (Figure 5).

If one is to compare the *V*_max_(X) value with the ESP on the other non-hydrogen atoms on the neutral halogenopyridine molecules (i.e., their corresponding π-holes, perpendicular to the plane of the ring), one can see that a σ-hole on the halogen is in all cases the most positive feature. This dramatically changes upon adding a positive charge on a pyridine ring. The added charge is distributed over the entire pyridine ring, leading to an increase in the ESP of all atoms of the molecule (most prominently that of the nitrogen atom and the neighboring carbon atoms). As a result, the σ-hole of the halogen atom does not necessarily correspond to the most positive region on the cation. Indeed, only in the case of iodopyridines and *o*-bromopyridine does the *V*_max_(X) value remain larger than the mean ESP of the nitrogen and carbon atoms of the pyridine ring. However, even in these structures the ESP on the π-holes of the nitrogen and carbon atoms remains significant and in the structure of [**4-IPy**Me]I leads to the appearance of a short iodide···carbon contact corresponding to an anion···π interaction; with the ESP carbon atom π-hole the *V*_max_(C) value of 436 kJ mol^–1^ e^–1^ is only slightly lower than that of the iodine atom σ-hole (456 kJ mol^–1^ e^–1^).

The most ubiquitous contacts in all of the crystal structures have been the C–H···I^–^ hydrogen bonds. If one is to compare the *V*_max_(X) value with the ESP of hydrogen atoms of the pyridine ring (*V*_max_(H)), in neutral halogenopyridines, the mean *V*_max_(H) value is more positive than the σ-hole of the halogen in all cases except for *m*- and *p*-iodopyridines. In the cations the *V*_max_(H) values of all the hydrogen atoms increase dramatically so that almost all hydrogen atoms of the aromatic ring in the *m*- and *p*-iodopyridinium cations become more positive than the *V*_max_(X) value of the iodine. However, in both *o*-iodopyridinium cations the *V*_max_(X) value is sufficiently increased so that it becomes more positive than the aromatic hydrogen atoms, with the exception of the hydrogen atom in the position *ortho* to the pyridinium nitrogen. Of course, in the case of all protonated halogenopyridinium cations the hydrogen located on the nitrogen atom is by far the most positive feature of the molecule (the *V*_max_(H) value being in the range ca. 665–690 kJ mol^–1^ e^–1^, exceeding the *V*_max_(X) value by ca. 150–310 kJ mol^–1^ e^–1^, depending on the cation). It is therefore evident that, in the case of protonated halogenopyridinium cations, the hydrogen bond formed by the N–H group will always be the dominant interaction, while in the *N*-methylated cations a competition between the C–H hydrogen-bond donors and C–X halogen-bond donors can always be expected, even in the case of iodopyridinium cations.

**Figure 6:**
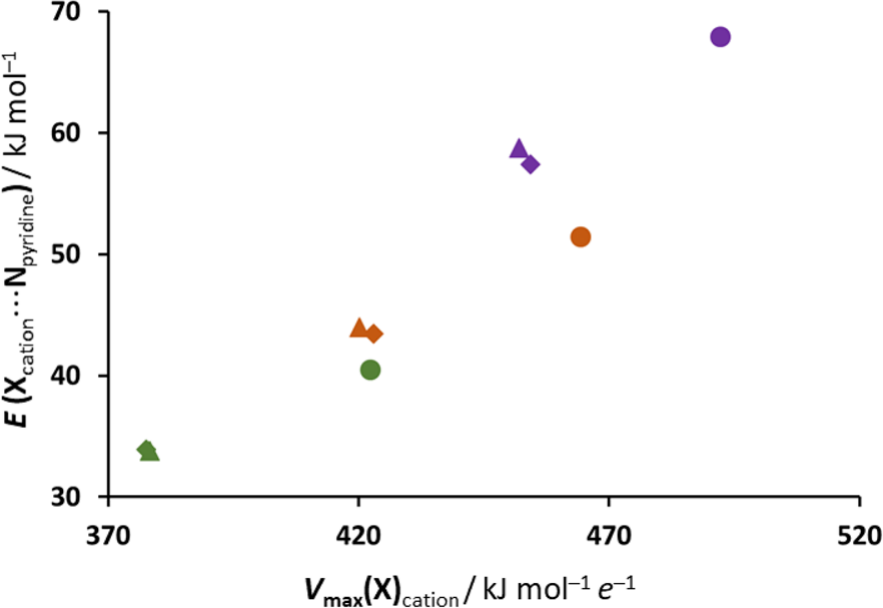
Correlation of *V*_max_(X) values of *N*-methylated halogenopyridines with energies of halogen bonds between a (neutral) pyridine molecule and *N*-methylated halogenopyridines.

In order to correlate the *V*_max_(X) values with halogen-bonding energies, we have computed the binding energies of a pyridine molecule (a neutral halogen bond acceptor) to *N*-methylated halogenopyridines (Figure 6). The bond energies obtained were quite considerable: ca. 34–40 kJ mol^–1^ for chloropyridinium, 43–51 kJ mol^–1^ for bromopyridinium, and 57–68 kJ mol^–1^ for iodopyridinium cations. Although the absolute values of the obtained energies may be somewhat untrustworthy, they do seem to indicate that the binding energies follow the same general trend as do the *V*_max_(X) values within each series of isomers—the *meta* and *para* isomers form bonds with energies very similar to one another, and the *ortho* isomer forms a bond which is ca. 6–10 kJ mol^–1^ stronger. Indeed, the bond energies for chloropyridines and bromopyridines are very closely linearly dependent on the *V*_max_(X) values for the free cations (*R*^2^ = 0.96), while the bond energies for iodopyridinium cations are somewhat higher: although *V*_max_(Br) on the **2-BrPy**Me^+^ cation is ca. 40 kJ mol^–1^ e^–1^ larger than *V*_max_(I) on **3-IPy**Me^+^ and **4-IPy**Me^+^ cations, the energy of the Br···N halogen bond it forms with a pyridine molecule is ca. 6–7 kJ mol^–1^ less than those of the I···N halogen bonds formed by **3-IPy**Me^+^ and **4-IPy**Me^+^. This implies that the Cl···N and the Br···N halogen bonds formed by the halogenopyridinium cations are primarily electrostatic, while the I···N halogen bonds have a more significant charge-transfer component.

The rather high bond energies for all of the studied cases show that all halogenopyridinium cations form rather strong halogen bonds. This would seem to be in contradiction with the fact that chloropyridinium cations participate only rarely in halogen bonding. However, as the positive charge is distributed over the entire molecule, leading to numerous local maxima of the ESP, the halogen bond formed through the chloropyridinium chlorine σ-hole is not necessarily the most favorable interaction between the cation and the pyridine molecule. On the other hand, the fact that chloropyridinium cations do participate in halogen-bonding contacts in almost 50% of the crystal structures clearly shows that the behavior of chlorine as a donor atom cannot be accounted for solely by the ESP of the σ-hole—a significant role must also be played by other contributions such as the size of the halogen atom (making the approach of the Lewis base easier) and possibly dispersion.^86^

## Conclusion

Although both protonation and *N*-methylation of halogenopyridines lead to a considerable increase in the ESP of the halogen σ-hole, a closer inspection of the distribution of the charge on the resulting cations clearly demonstrates that the halogen σ-hole is generally not the most positive site on the surface of the cation. This is most pronounced in the case of chloropyridinium cations, where the electrostatic potential maxima on almost all hydrogen and carbon atoms of the cation are more positive than the chlorine σ-hole. Such a charge distribution leads to the observed relatively low percentage of occurrence of halogen bonds with chloropyridinium chlorine donors in the CSD and the long C–Cl···I^–^ contacts encountered in the structures of chloropyridinium iodides. Even among iodopyridinium cations, only in those derived from **2-IPy** (with the iodine atom on the first neighbor of the “charged” nitrogen) is the iodine σ-hole ESP more positive than that of the majority of hydrogen atoms. Consequently, they form the shortest C–I···I^–^ halogen bonds both among protonated and methylated halogenopyridinium iodides. In spite of this, cations derived from not only all iodopyridines but also those derived from bromopyridines form halogen bonds with remarkable consistency—they have formed halogen-bonding contacts shorter than the sum of the van der Waals radii in all of the iodide salts and have been found to act as halogen bond donors in a formidable 90% of structures deposited in the CSD which comprise an iodo- or bromopyridinium cation and an appropriate acceptor. It is therefore probable that the lower ESP on the halogen σ-hole is compensated by the size of the halogen atom: enabling on the one hand a closer approach of a Lewis base (with less steric hindrance to the halogen σ-hole as opposed to the hydrogen atoms of the pyridine ring) and on the other hand a larger potential contact surface between the contact atoms. This, however, does not exclude the formation of competing interactions, as evidenced in the structures of iodides by numerous short C–H···I^–^ and even π···I^–^ contacts between the cations and the iodide. Therefore, the halogenopyridinium cations, in spite of much larger *V*_max_(X) values, have not been shown to be superior in comparison to “classical” neutral halogen bond donors.

This having been said, although adding a positive charge on the halogenopyridine ring forms numerous potential binding sites for Lewis bases in bromopyridinium and particularly in iodopyridinium cations, binding of the base to the halogen atom is likely to occur. This makes halopyridinium cations (particularly iodopyridinium) fairly reliable halogen-bond donors and thus justifies their use in the development of design strategies for the deliberate synthesis of halogen-bonded supramolecular structures.

## Experimental Section

### Synthesis

Halogenopyridines (**2-ClPy**, **2-BrPy**, **2-IPy**, **3-ClPy**, **3-BrPy**, **3-IPy** and **4-IPy**), methyl iodide, and solvents were purchased from Sigma-Aldrich and used as received. **4-ClPy** was prepared by dissolving 4-hydroxypyridine (700 mg, 6 mmol) in thionyl chloride (5 mL) and heating the mixture until the evolution of HCl gas ceased, after which the excess thionyl chloride was removed by distillation. To the remaining solid was added diethyl ether, and the mixture was treated with aqueous sodium hydrogencarbonate. The ether layer was separated and dried over sodium sulfate. The resulting solution was directly used in further synthesis.

*N*-Methylhalogenopyridinium iodides (except for [**4-ClPy**Me]I) were obtained by dissolving the halogenopyridine (1 mmol) in hot acetone and adding methyl iodide in excess (ca. 20% above the stoichiometric amount), whereupon the solutions were left to cool and evaporate. [**4-ClPy**Me]I was prepared in a similar fashion by adding methyl iodide to an ether solution obtained as described above. Solid products appeared in 1–3 days. Single crystals (suitable for single-crystal X-ray diffraction experiments) of [**2-IPy**Me]I, [3**-ClPy**Me]I, [**3-BrPy**Me]I, [**3-IPy**Me]I, [**4-ClPy**Me]I and [**4-IPy**Me]I were prepared by the synthesis procedure or by recrystallizing the obtained salt from a mixture of ethanol and water.

Single crystals (suitable for a single-crystal X-ray diffraction experiment) of [**2-ClPy**Me]I were prepared by recrystallizing the obtained salt from a mixture of ethanol, acetone, and water.

In the case of [**2-BrPy**Me]I, all attempts at crystallization led to partial or complete replacement of covalently bonded bromine with iodine and subsequent crystallization of mixed crystals containing both [**2-BrPy**Me]^+^ and [**2-IPy**Me]^+^ cations on equivalent crystallographic positions. This has precluded us from obtaining single crystals of [**2-BrPy**Me]I suitable for study.

FT-IR spectra (see the Supporting Information) of all prepared salts were recorded on a PerkinElmer Spectrum Two FTIR spectrometer using the attenuated total reflectance (ATR) technique.

[**2-ClPy**Me]I: selected IR data (cm^–1^) 1617 (C=N)_py_, 1567 (C=C)_ring_, 1439 (N–C)_methyl_, 2950–3050 (C–H); yield 116 mg (45.4%).

[**2-IPy**Me]I: selected IR data (cm^–1^) 1616 (C=N)_py_, 1500 (C=C)_ring_, 1436 (N–C)_methyl_, 2900–3050 (C–H); yield 91 mg (26.2%).

[3**-ClPy**Me]I: selected IR data (cm^–1^) 1620 (C=N)_py_, 1560 (C=C)_ring_, 1484 (N–C)_methyl_, 2950–3050 (C–H); yield 183 mg (71.6%).

[**3-BrPy**Me]I: selected IR data (cm^–1^) 1622 (C=N)_py_, 1486 (C=C)_ring_, 1460 (N–C)_methyl_, 2950–3100 (C–H); yield 231 mg (77%).

[**3-IPy**Me]I: selected IR data (cm^–1^) 1622 (C=N)_py_, 1489 (C=C)_ring_, 1453 (N–C)_methyl_, 2940–3070 (C–H); yield 276 mg (79.6%).

[**4-ClPy**Me]I: selected IR data (cm^–1^) 1627 (C=N)_py_, 1495 (C=C)_ring_, 1465 (N–C)_methyl_, 2850–3070 (C–H); yield 93 mg (6.1%).

[**4-IPy**Me]I: only a few crystals were isolated, which were used for X-ray and thermal analysis.

### X-ray Diffraction Measurements

All single-crystal X-ray diffraction experiments were performed using an Oxford Diffraction XtaLAB Synergy, Dualflex, HyPix Xray four-circle diffractometer with mirror-monochromated Mo Kα (λ = 0.71073 Å) radiation. The data sets were collected using the ω-scan mode over a 2θ range up to 60°. The programs CrysAlis PRO CCD and CrysAlis PRO RED were employed for data collection, cell refinement, and data reduction.^87,88^ The structures were solved by SHELXT or by direct methods using SHELXS and refined using SHELXL programs.^89,90^ The structural refinement was performed on *F*^2^ using all data. The hydrogen atoms were placed in calculated positions and treated as riding on their parent atoms (C–H = 0.93 Å and *U*_iso_(H) = 1.2[*U*_eq_(C)] for aromatic hydrogen atoms; C–H = 0.97 Å and *U*_iso_(H) = 1.5[*U*_eq_(C)] for methyl hydrogen atoms). All calculations were performed using the WinGX or Olex2 1.3-ac4 crystallographic suite of programs.^91^ A summary of data pertinent to X-ray crystallographic experiments is provided in Table S1 in the Supporting Information. Further details are available from the Cambridge Crystallographic Centre (CCDC 2089226–2089232 contain crystallographic data for this paper). The molecular structures of compounds and their packing diagrams were prepared using Mercury.^92^

### Thermal Analysis

Differential scanning calorimetry (DSC) and thermogravimetric (TG) measurements were performed simultaneously on a Mettler-Toledo TGA/DSC 3+ module (Mettler Toledo, Greifensee, Switzerland). Samples were placed in alumina crucibles (40 μL) and heated from 25 to 300 °C, at a heating rate of 10 °C min^–1^ under a nitrogen flow of 150 mL min^–1^.

Data collection and analysis were performed using the program package STARe (Version 15.00, Mettler Toledo, Greifensee, Switzerland).^93^ TG and DSC thermograms of the prepared compounds are shown in Figures S11–S15 in the Supporting Information.

### Database Survey

A data survey has been performed on the CSD database, version 5.42 (May 2021) with three updates using ConQuest Version 2020.3.0. For hydrogen- and halogen-bonded contacts, the upper limit of the distance between the donor atom (hydrogen or iodine) and acceptor ions (Cl^–^, Br^–^, I^–^) was defined as the sum of their van der Waals radii. Nitrogen halogenoheterocycles were defined as containing a six-membered aromatic ring with one nitrogen atom and a halogen substituent on the *ortho*, *meta*, or *para* position. Perfluorinated iodobenzenes were searched as the aromatic ring of six carbon atoms with iodo and fluoro substituents in *ortho* positions. The resulting hits were checked manually.

In order to ascertain the frequency of halogen bonding, for each donor a pair of searches were made. One was for structures with a close contact of a donor halogen and nitrogen, oxygen, chloride, bromide, or iodide (fluoride was excluded because of the extremely small number of structures). The other search was for structures containing both the donor and the potential acceptor (defined as above) without the two necessarily being in short contact. From these the structures that contained only quaternary nitrogen (which cannot act as halogen bond acceptor) were removed manually. The incidence of each donor type was than calculated as the ratio of the number of hits obtained by the two searches.

### Computational Details

All calculations were performed using the Gaussian 09 software package.^94^ Geometry optimizations were performed using the M062X/dgdzvp level of theory,^95−97^ with an ultrafine integration grid (99 radial shells and 590 points per shell). This method has been shown to reproduce experimental halogen-bond energies in the gas phase with good accuracy, which are comparable to energies obtained by using larger and more time consuming triple-ζ basis sets.^98^ Harmonic frequency calculations were performed on the optimized geometries to ensure the success of each geometry optimization. The figures were prepared using GaussView.^99^

## Supporting Information Available

Crystallographic data for all compounds, ORTEP representations of the formula units of the prepared compounds, overview of supramolecular interactions in iodides of *N*-methylated and protonated halogenopyridinium cations, TG and DSC curves, total electron energies and Cartesian coordinates for optimized structures of *N*-methylated, protonated, and neutral halogenopyridines, molecular electrostatic potentials (MEPs) on halogen, nitrogen, carbon, and hydrogen atoms in *N*-methylated, protonated, and neutral halogenated pyridines, and bonding energies and Cartesian coordinates for optimized complexes of *N*-methylated halogenopyridinium cations and a neutral pyridine molecule (PDF)

## Accession Codes

CCDC 2089226–2089232 contain the supplementary crystallographic data for this paper. These data can be obtained free of charge via www.ccdc.cam.ac.uk/data_request/cif, or by emailing data_request@ccdc.cam.ac.uk, or by contacting The Cambridge Crystallographic Data Centre, 12 Union Road, Cambridge CB2 1EZ, UK; fax: +44 1223 336033.

## Abbreviations

**2-ClPy**: 2-chloropyridine
**2-BrPy**: 2-bromopyridine
**2-IPy**: 2-iodopyridine
3**-ClPy**: 3-chloropyridine
**3-BrPy**: 3-bromopyridine
**3-IPy**: 3-iodopyridine
**4-ClPy**: 4-chloropyridine
**4-BrPy**: 4-bromopyridine
**4-IPy**: 4-iodopyridine
Me: methyl group
CSD: Cambridge Structural Database
X: halogen
*V*_min_: minimum electrostatic potential of the corresponding atom
*V*_max_: maximum electrostatic potential of the corresponding atom
DSC: differential scanning calorimetry
TG: thermogravimetric
