# Supplementary material for: Evaluation of Halogenopyridinium Cations as Halogen Bond Donors
Source: Cryst Growth Des. 2021 Nov 8;21(12):6889–901. doi: 10.1021/acs.cgd.1c00805 (PMC8641392; doi:10.1021/acs.cgd.1c00805)
Supplement: Supplementary file 1 — cg1c00805_si_001.pdf [file cg1c00805_si_001.pdf]

SUPPORTING INFORMATION

Evaluation of halogenopyridinium cations as halogen bond donors

Luka Fotović, Nikola Bedeković and Vladimir Stilinović

Department of Chemistry, Faculty of Science, University of Zagreb, Horvatovac 102a, HR-10000 Zagreb, Croatia

Email: vstilinovic@chem.pmf.hr
Fax: +385 1 4606 341
Tel: +385 1 4606 371

Table of Contents

Item	Page
**Table S1** (Crystallographic data)	**3**
**Figures S1–S7** (ORTEP representations of the formula units of the prepared compounds)	**5**
**Table S2.** An overview of supramolecular interactions in iodides of *N*-methylated halogenopyridinium cations at room temperature.	**9**
**Table S3.** An overview of supramolecular interactions in iodides of protonated halogenopyridinium cations at room temperature.	**11**
**Figures S8–S14** (DSC and TG curves of the prepared compounds)	**12**
**Figures S15–S20** (IR spectra of the prepared compounds)	**16**
Total electron energies and Cartesian coordinates for optimised structures of halogenopyridines:	**18**
Total electron energies and Cartesian coordinates for optimised structures of protonated halogenopyridines:	**23**
Total electron energies and Cartesian coordinates for optimised structures of *N*-methylated halogenopyridines:	**28**
Total electron energies and Cartesian coordinates for optimised structures of the complexes of *N*-methylated halogenopyridinium cations with pyridine	**34**
**Table S4.** Molecular electrostatic potentials (*MEP*s) on halogen, nitrogen and carbon atoms in neutral halogenated pyridines. All values are in kJ mol^{-1} e^{-1}.	**45**

**Table S5.** Molecular electrostatic potentials (*MEP*s) on hydrogen atoms in neutral halogenated pyridines. All values are in kJ mol^{-1} e^{-1}.	**45**
**Table S6.** Molecular electrostatic potentials (*MEP*s) on halogen, nitrogen and carbon atoms in halogenpyridinium cations. All values are in kJ mol^{-1} e^{-1}.	**46**
**Table S7.** Molecular electrostatic potentials (*MEP*s) on hydrogen atoms in halogenpyridinium cations. All values are in kJ mol^{-1} e^{-1}.	**46**
**Table S8.** Molecular electrostatic potentials (*MEP*s) on halogen, nitrogen and carbon atoms in methylpyridinium cations. All values are in kJ mol^{-1} e^{-1}.	**47**
**Table S9.** Molecular electrostatic potentials (*MEP*s) on hydrogen atoms in methylpyridinium cations. All values are in kJ mol^{-1} e^{-1}.	**47**
**Table S10.** Binding energies for the pyridine-methylpyridinium cation complexes	**48**
**Figure S21**. Positions in molecules and ions from which the values of molecular electrostatic potentials were used for an analysis.	**49**
**Tables S10-12.** Results of CSD survay on halogen bonding of neutral, protonated and *N*-methylated halogenoheterocycles as halogen bond donors and oxygen, nitrogen, chloride, bromide and iodide as halogen bond acceptor.	**49**
List of refcodes covered by the CSD survay	**51**
**Table S13.** Halogen bond lengths (CSD data for structures measured at room temperature) for halogen bonds with N, O and I^- as acceptors and chloroheterocycles as donors.	**66**
**Table S14.** Halogen bond lengths (CSD data for structures measured at room temperature) for halogen bonds with N, O and I^- as acceptors and bromoheterocycles as donors.	**66**

Table S1. Crystal data and refinement details for the prepared salts.

	N-Me-2ClPy⁺ I⁻	*N*-Me-2IPy⁺ I⁻	*N*-Me-3ClPy⁺ I⁻	*N*-Me-3BrPy⁺ I⁻
Molecular formula	C_{84} H_{98} Cl_{14} I_{14} N_{14}	$C_6H_7I_2N$	C_6H_7NICl	C_6H_7BrIN
M_r	3576.66	346.9	255.48	299.9
Crystal system	monoclinic	monoclinic	orthorhombic	orthorhombic
Space group	$P\ 2_1/c$	$P\ 2_1/c$	*P* bca	*P* bcm
Crystal data:				
a / Å	12.6587(4)	6.6729(6)	11.3962(4)	10.3370(3)
b / Å	20.9967(6)	10.8593(12)	8.5424(3)	12.1641(3)
c / Å	11.3612(4)	14.1704(18)	17.1511(7)	7.3528(2)
α / °	90	90	90	90
β / °	91.667(3)	118.093(1)	90	90
γ / °	90	90	90	90
V / Å^3	3018.43	905.853	1669.68	924.543
Z	1	4	8	4
D_{calc} / g cm^{-3}	1.968	2.544	2.561	2.155
λ(MoK_α) / Å	0.71073	0.71073	0.71073	0.71073
T / K	170	293	295	295
Crystal size / mm^3	0.06 x 0.10 x 0.14	0.15 x 0.17 x 0.04	0.18 x 0.06 x 0.04	0.04 x 0.08 x 0.06
μ / mm^{-1}	3.943	6.868	4.073	7.711
F(000)	1680.0	624.0	960	552
Refl. collected/unique	51846 / 42521	11161 / 2584	17328 / 2215	29409 / 1007
Parameters	292	83	84	56
$\Delta\rho_{max}$, $\Delta\rho_{min}$ / e Å^{-3}	3.124; –2.013	1.171; –2.151	0.530; –0.710	0.284; –0.236
$R[F^2 > 4\sigma(F^2)]$	0.0831	0.0432	0.0252	0.0252
w$R(F^2)$	0.2695	0.1140	0.0617	0.0517
Goodness-of-fit, S	1.248	1.009	1.029	1.120

Table S1. Continued.

	***N*-Me-3IPy**$^+$ I$^-$	N-Me-4ClPy+ I-	***N*-Me-4IPy**$^+$ I$^-$
Molecular formula	$C_6H_7I_2N$	C6H7NICl	$C_6H_7I_2N$
M_r	255.48	255.48	255.48
Crystal system	orthorhombic	triclinic	triclinic
Space group	*C* mca	P –1	*P* –1
Crystal data:			
a / Å	7.3058(6)	6.0048(2)	5.0538(3)
b / Å	12.2654(6)	7.2529(2)	7.4109(4)
c / Å	20.9488(14)	10.2302(2)	12.5936(6)
α / °	90	102.575(2)	90.511(4)
β / °	90	106.111(2)	91.413(4)
γ / °	90	91.628(2)	104.845(5)
V / Å^3	1877.19	415.902	455.732
Z	8	2	2
D_{calc} / g cm^{-3}	2.455	2.04	2.528
λ(MoK_α) / Å	0.71073	0.71073	0.71073
T / K	293	295	295
Crystal size / mm^3	0.18 x 0.07 x 0.08	0.04 x 0.09 x 0.15	0.01 x 0.1 x 0.15
μ / mm^{-1}	6.628	4.088	9.782
F(000)	1248.00	240	312
Refl. collected/unique	4661 / 987	12968 / 2429	5523 / 2773
Parameters	57	83	83
$\Delta\rho_{max}$, $\Delta\rho_{min}$ / e Å^{-3}	1.141; –1.265	0.624;-0.895	1.034; –0.867
R[F^2 > 4[illegible](F^2)]	0.0513	0.0289	0.0381
w*R*(F^2)	0.1487	0.0720	0.0937
Goodness-of-fit, *S*	1.101	1.034	0.985

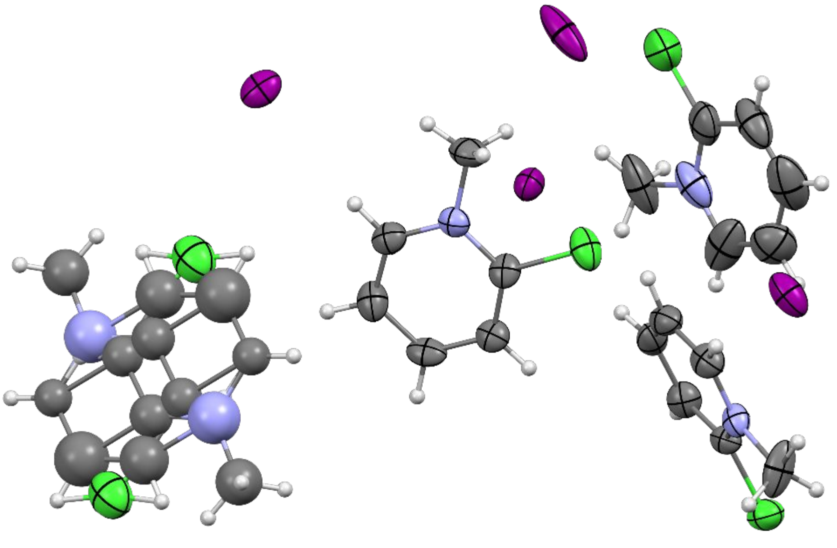

Figure S1. Molecular structure of ***N*-Me-2ClPy**$^{+}$ I^{-} showing the atom-labelling scheme. Displacement ellipsoids are drawn at the 50 % probability level, and H atoms are shown as small spheres of arbitrary radius.

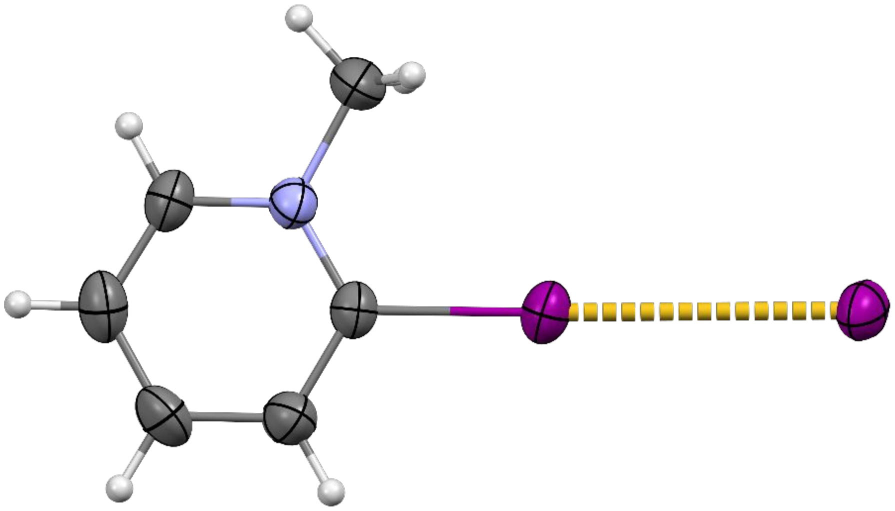

Figure S2. Molecular structure of ***N*-Me-2iPy**$^{+}$ I^{-} showing the atom-labelling scheme. Displacement ellipsoids are drawn at the 50 % probability level, and H atoms are shown as small spheres of arbitrary radius.

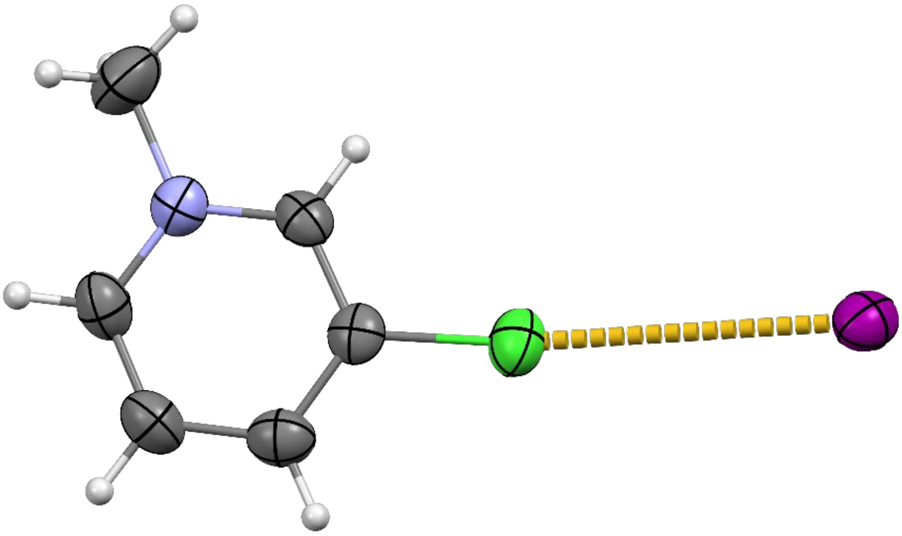

Figure S3. Molecular structure of ***N*-Me-3ClPy**$^{+}$ I^{-} showing the atom-labelling scheme. Displacement ellipsoids are drawn at the 50 % probability level, and H atoms are shown as small spheres of arbitrary radius.

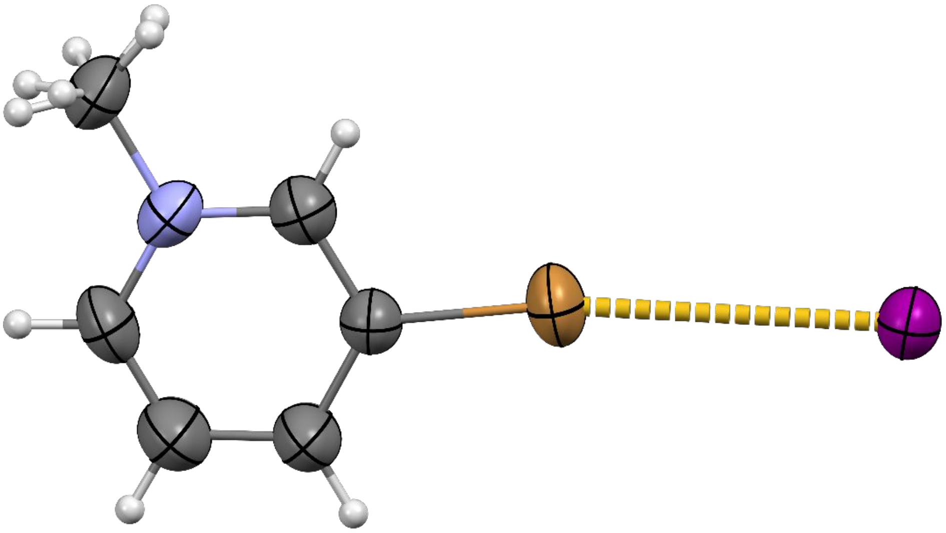

Figure S4. Molecular structure of ***N*-Me-3BrPy**$^{+}$ I^{-} showing the atom-labelling scheme. Displacement ellipsoids are drawn at the 50 % probability level, and H atoms are shown as small spheres of arbitrary radius.

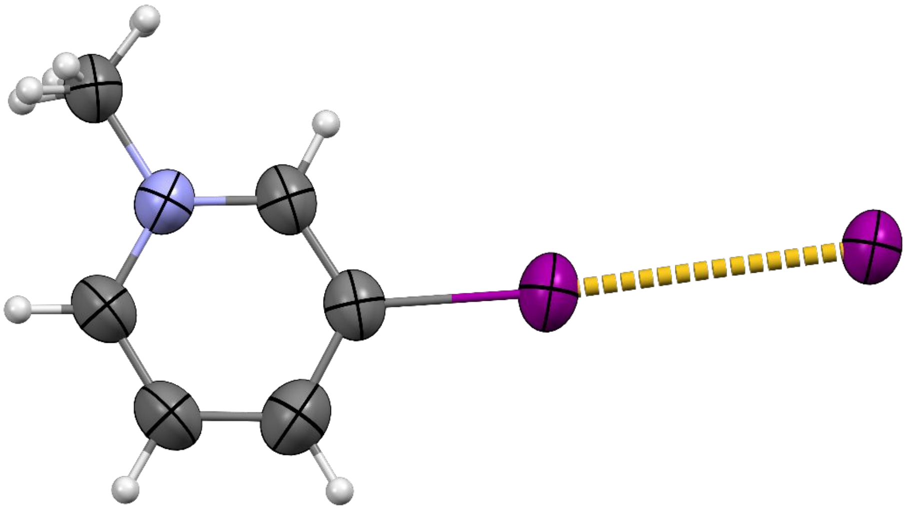

Figure S5. Molecular structure of ***N*-Me-3IPy**$^+$ I$^-$ showing the atom-labelling scheme. Displacement ellipsoids are drawn at the 50 % probability level, and H atoms are shown as small spheres of arbitrary radius.

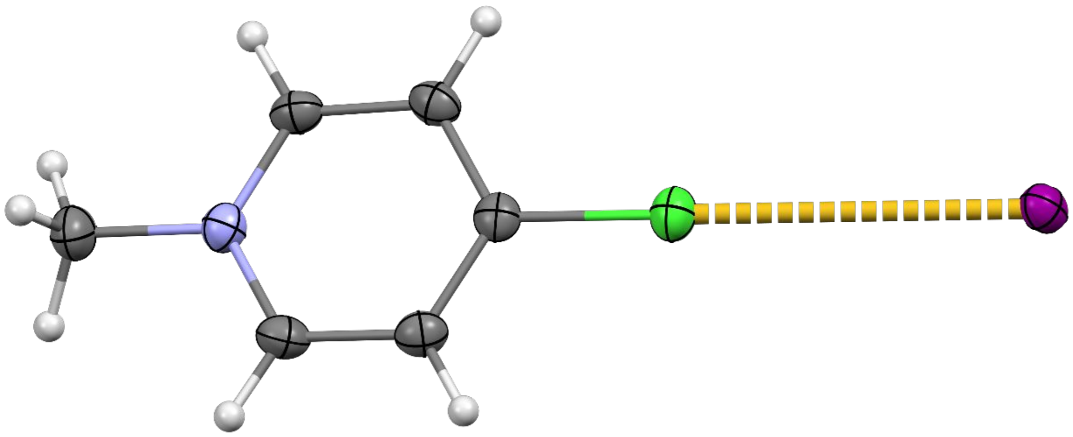

Figure S6. Ortep plot of the asymmetric unit of ***N*-Me-4cLPy**$^+$ I$^-$ showing the atom-labelling scheme. Displacement ellipsoids are drawn at the 50 % probability level, and H atoms are shown as small spheres of arbitrary radius.

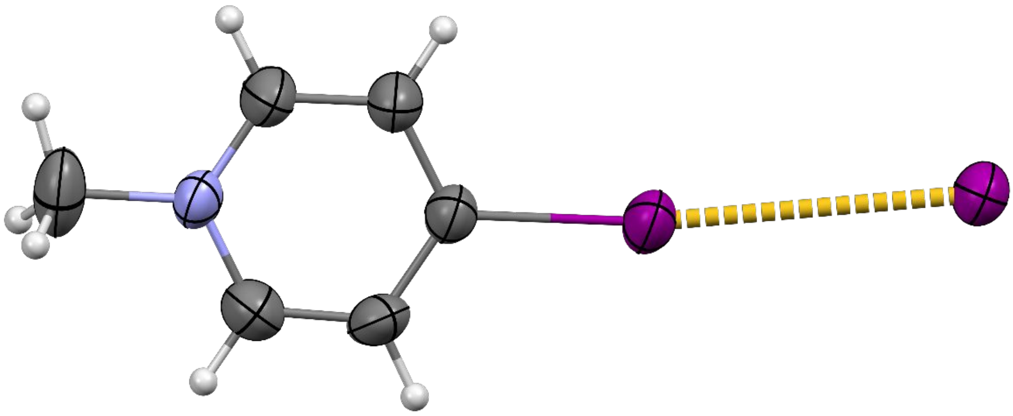

Figure S7. Ortep plot of the asymmetric unit of ***N*-Me-4IPy**$^+$ I$^-$ showing the atom-labelling scheme. Displacement ellipsoids are drawn at the 50 % probability level, and H atoms are shown as small spheres of arbitrary radius.

Table S2. An overview of supramolecular interactions in iodides of *N*-methylated halogenopyridinium cations at room temperature.

***N*-methylated halogenopiridinium iodides**	**Atom1**	**Atom2**	**Length**	**Length-VdW**	***R.S.* / %**	**ESP**
**[2-ClPyMe]I**	Cl3	I2	3.496	-0.234	6.27	422.4
	Cl2	I3	3.509	-0.221	5.92	422.4
	Cl4	I2	3.511	-0.219	5.87	422.4
	H17	I3	2.994	-0.186	5.85	534.6
	H4	I3	3.015	-0.165	5.19	478.0
	H10	I3	3.041	-0.139	4.37	478.0
	H8	I1	3.043	-0.137	4.31	480.6
	C15	C10	3.264	-0.136	4.00	413.8
	H14	I2	3.046	-0.134	4.21	480.6
	H15	I1	3.073	-0.107	3.36	478.0
	H6A	I4	3.084	-0.096	3.02	472.7
	H2	I1	3.088	-0.092	2.89	480.6
	H5	I2	3.093	-0.087	2.74	534.6
	H11	I2	3.104	-0.076	2.39	534.6
	C15	C11	3.330	-0.070	2.06	413.8
	H18C	Cl2	2.887	-0.063	2.14	472.7
	Cl1	Cl2	3.502	0.002	-0.06	422.4
	C11	I4	3.687	0.007	-0.19	452.1
	C1	I2	3.688	0.008	-0.22	474.0
	H16	Cl1	2.958	0.008	-0.27	478.0
**[2-IPyMe]I**	I1	I2	3.459	-0.501	12.65	492.32
	H6C	I2	3.114	-0.066	2.08	463.55
	H4	I2	3.172	-0.008	0.25	471.16
	H6A	I2	3.178	-0.002	0.06	468.37
	H3	I2	3.195	0.015	-0.47	465.90
	H5	I2	3.228	0.048	-1.51	521.55
**[3-ClPyMe]I**	Cl1	I1	3.776	0.046	-1.15	378.41
	H5	I1	3.048	-0.132	4.15	537.09
	H1	I1	3.113	-0.067	2.11	529.03
	C1	Cl1	3.406	-0.044	1.38	462.48
	C5	C5	3.404	0.004	-0.12	448.40
	C1	I1	3.687	0.007	-0.19	462.48
	H3	I1	3.193	0.013	-0.41	477.87
	C2	Cl1	3.485	0.035	-1.03	423.08
**[3-BrPyMe]I**	Br2	I2	3.637	-0.193	5.04	420.23

	H12C	I2	3.069	-0.111	3.49	397.45
	Br2	C12	3.525	-0.025	0.71	420.23
	H9	I2	3.166	-0.014	0.44	472.17
	H12B	I2	3.204	0.024	-0.75	410.08
	H12C	Br2	3.078	0.028	-0.92	397.45
	H11	I2	3.229	0.049	-1.54	533.28
**[3-IPyMe]I**	I1	I2	3.538	-0.422	10.66	452.06
	H6A	I2	3.025	-0.155	4.87	425.59
	C1	I2	3.661	-0.019	0.52	454.7
**[4-ClPyMe]I**	Cl1	I1	3.605	-0.125	3.4	377.6
	H1	I1	3.041	-0.139	4.37	548.1
	C3	C3	3.393	-0.007	0.21	427.0
	H6B	I1	3.198	0.018	-0.57	355.36
	H5	I1	3.213	0.033	-1.04	548.1
**[4-IPyMe]I**	I1	I2	3.552	-0.408	10.3	454.44
	H5	I2	3.083	-0.097	3.05	521.40
	C5	I2	3.646	-0.034	0.92	436.81
	H6A	I2	3.199	0.019	-0.60	454.07
	H4	I1	3.207	0.027	-0.85	470.11
	C1	I2	3.724	0.044	-1.19	436.46
	H1	I2	3.220	0.040	-1.26	521.40

Table S3. An overview of supramolecular interactions in iodides of protonated halogenopyridinium cations at room temperature.

Protonated halogenopiridinium iodides	Atom1	Atom2	Length	Length-VdW	*R.S.* / %	ESP
**[2-ClPyH]I**	H3	I1	2.549	-0.631	19.84	689.1
	Cl1	I1	3.768	0.038	0.10	434.7
	H1	I1	3.059	-0.121	3.81	492.9
	H5	I1	3.190	0.010	-0.31	492.9
**[2-BrPyH]I**	H4	I1	2.548	-0.632	19.87	677.7
	Br1	I1	3.575	-0.255	5.92	481.9

	H2	I1	3.206	0.026	-0.82	492.0
**[2-IPyH]I**	H1	I2	2.566	-0.614	19.31	665.5
	I1	I2	3.467	-0.493	12.45	511.3
	C6	I1	3.720	0.040	-1.09	449.0
**[3-ClPyH]I**	H5	I1	2.630	-0.550	17.30	700.2
	Cl1	I1	3.739	0.009	0.2	389.1
	H2	I1	3.000	-0.180	5.66	546.3
**[3-BrPyH]I**	H4	I1	2.743	-0.437	13.74	693.0
	Br1	I1	3.589	-0.241	6.3	437.9
	H3	I1	3.093	-0.087	2.74	487.5
	H2	I1	3.164	-0.016	0.50	546.3
**[3-IPyH]I**	H1	I1	2.813	-0.367	11.54	688.0
	I1	I2	3.516	-0.444	11.2	468.9
	H4	I1	3.098	-0.082	2.58	480.7
	H6	I1	3.151	-0.029	0.91	535.2
	C6	I1	3.706	0.026	-0.71	466.0
	C5	C2	3.427	0.027	-0.79	420.6
	H2	I2	3.220	0.040	-1.26	532.9
**[4-ClPyH]I**	H5	I1	2.601	-0.579	18.21	687.3
	Cl1	I1	3.733	0.003	-0.10	392.9
	H1	I1	3.044	-0.136	4.28	548.1
	H3	I1	3.088	-0.092	2.89	548.1
**[4-BrPyH]I**	H5	I1	2.632	-0.548	17.23	688.8
	Br1	I1	3.648	-0.182	4.8	438.9
	H1	I1	3.029	-0.151	4.75	542.8
	H3	I1	3.157	-0.023	0.72	542.8
**[4-IPyH]I**	H1	I2	2.719	-0.461	14.50	677.1
	I1	I2	3.532	-0.428	10.8	470.0
	H6	I2	3.084	-0.096	3.02	533.2
	H2	I2	3.158	-0.022	0.69	533.2
	C2	I2	3.710	0.030	-0.81	458.6

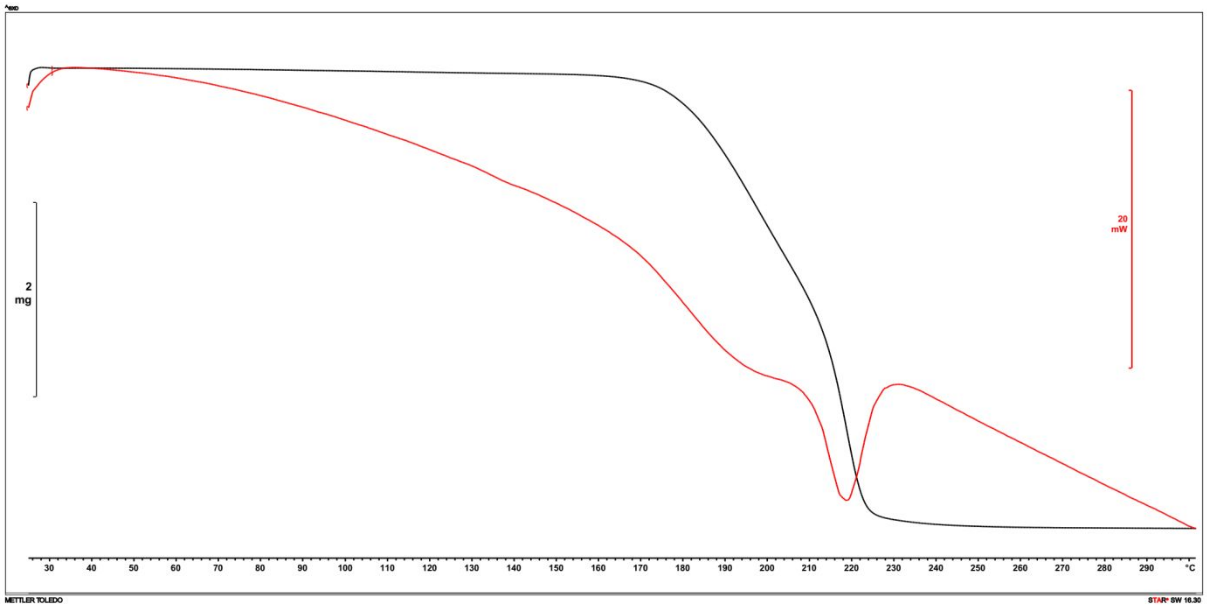

Figure S8. TG (black) and DSC (red) thermograms of ***N*-Me-2ClPy**$^+$ I$^-$.

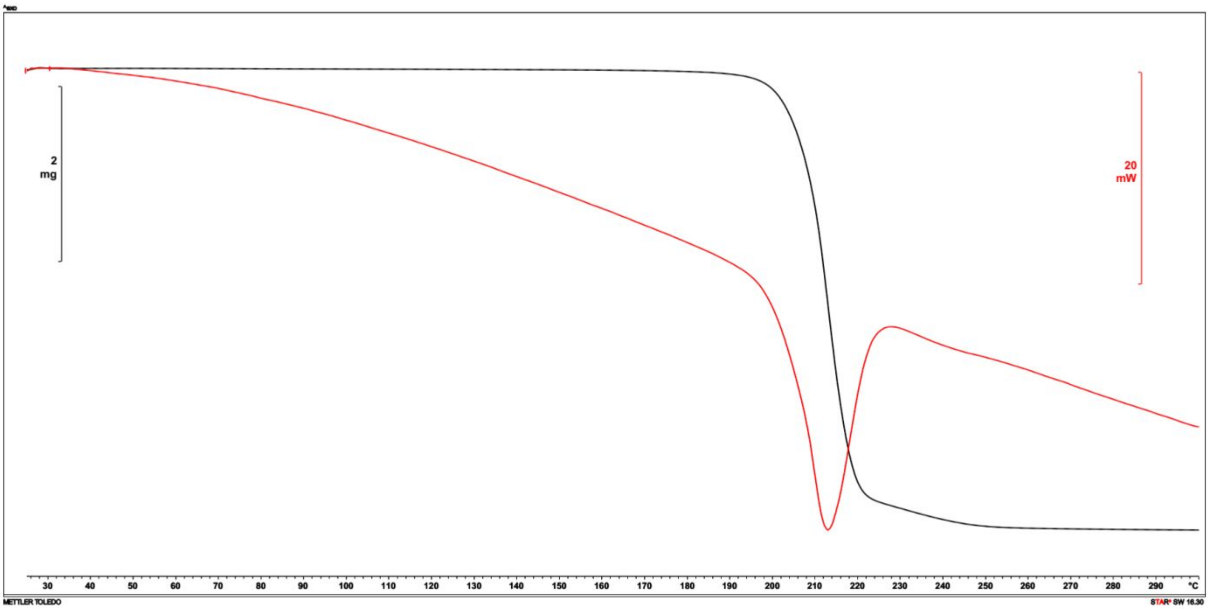

Figure S9. TG (black) and DSC (red) thermograms of ***N*-Me-2IPy**$^+$ I$^-$.

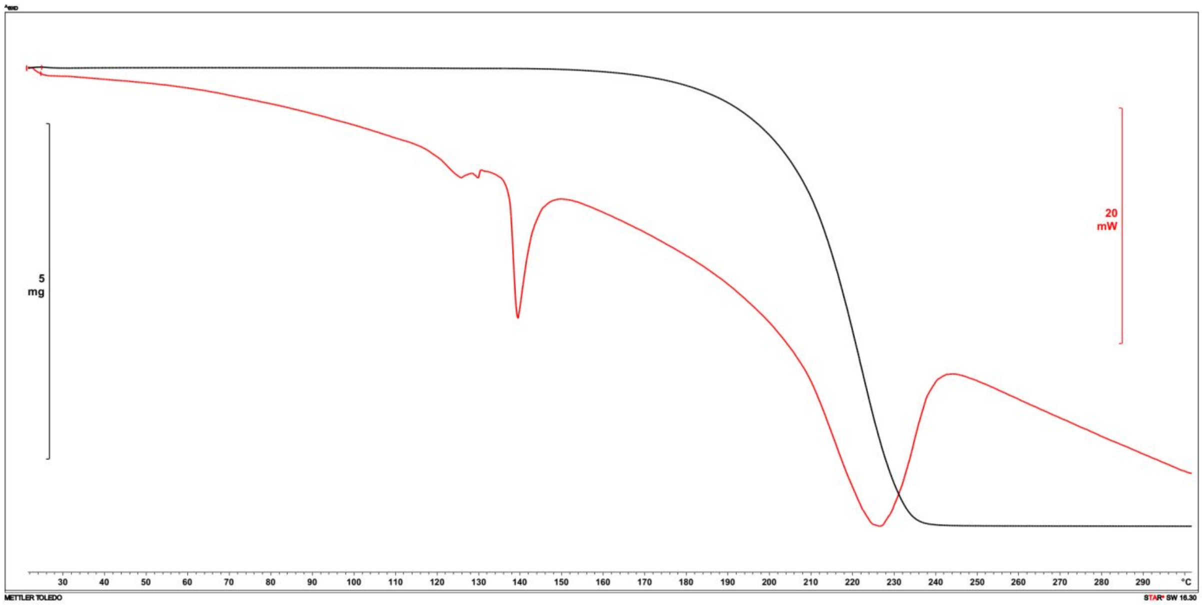

Figure S10. TG (black) and DSC (red) thermograms of ***N*-Me-3ClPy**$^+$ I$^-$.

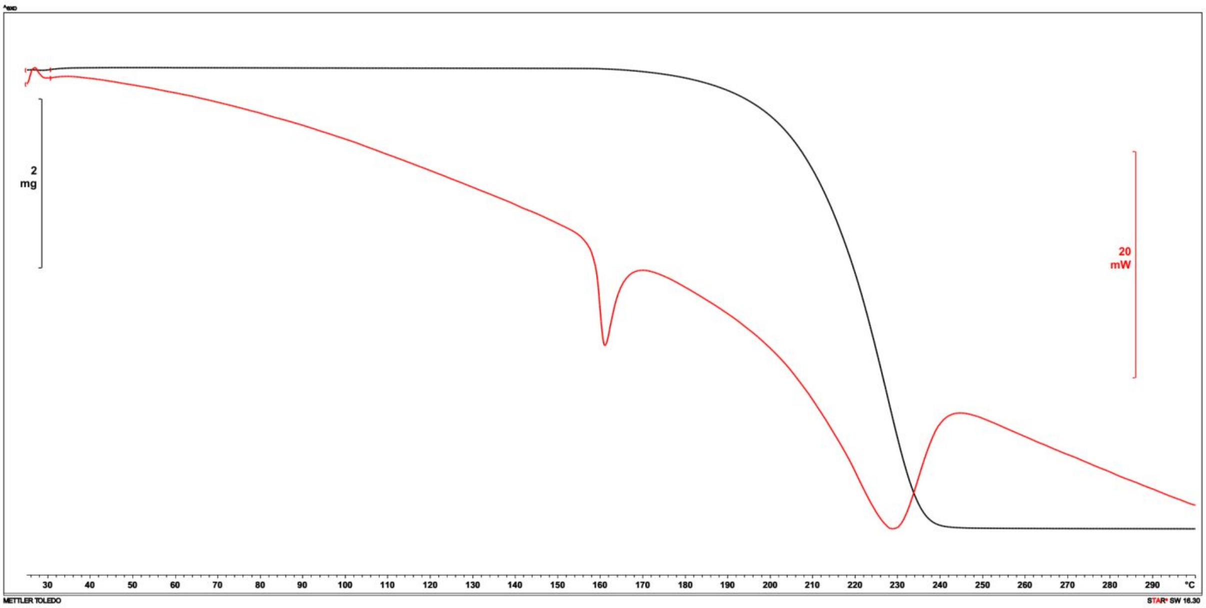

Figure S11. TG (black) and DSC (red) thermograms of ***N*-Me-3BrPy**$^+$ I$^-$.

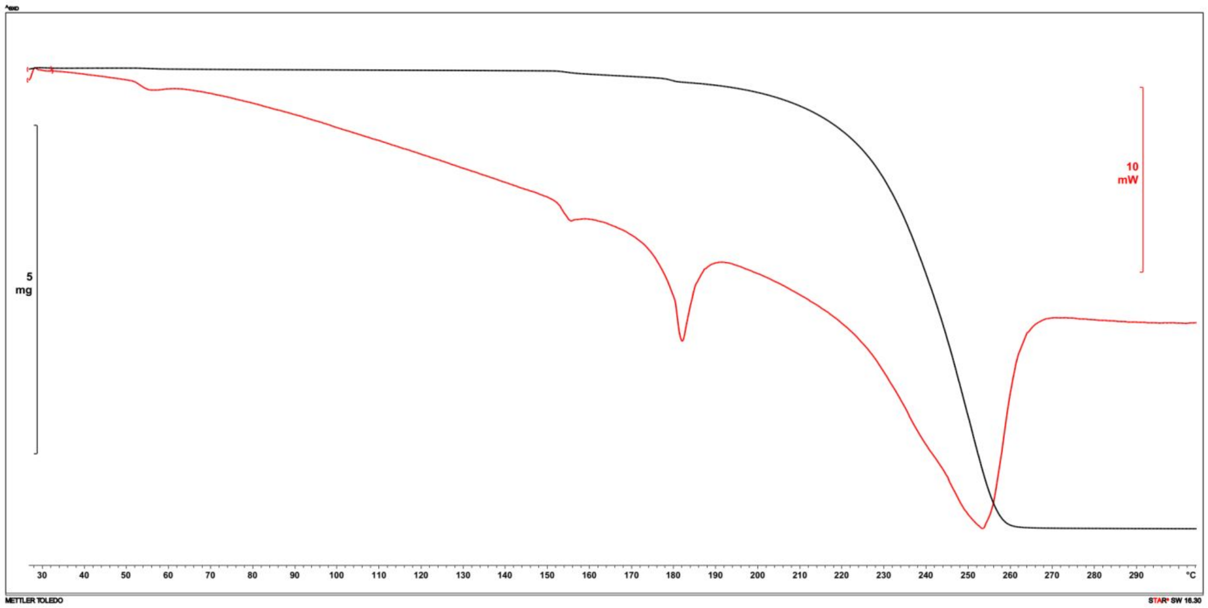

Figure S12. TG (black) and DSC (red) thermograms of ***N*-Me-3IPy**$^+$ I$^-$.

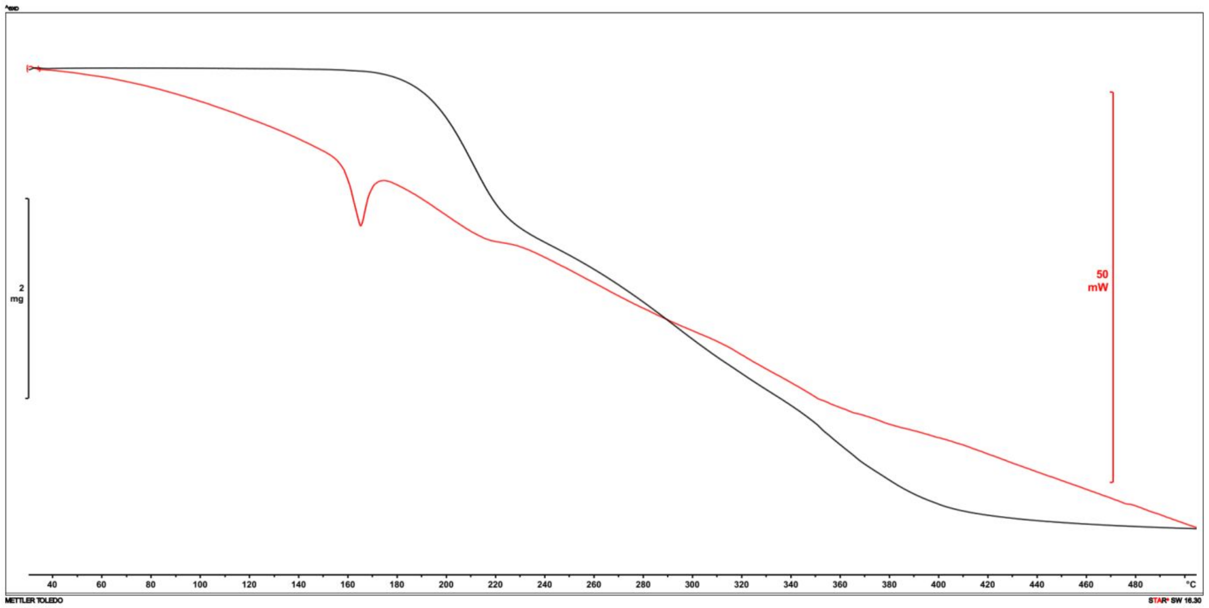

Figure S13. TGA (black) and DSC (red) thermograms of ***N*-Me-4ClPy**$^+$ I$^-$.

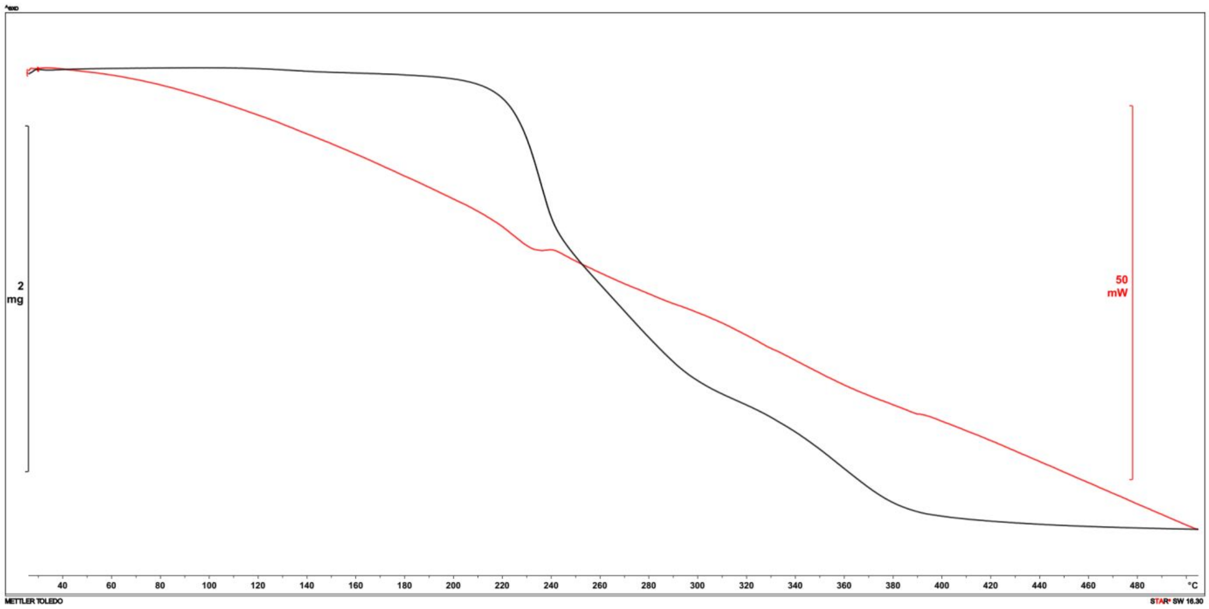

Figure S14. TGA (black) and DSC (red) thermograms of ***N*-Me-4IPy**$^+$ I$^-$.

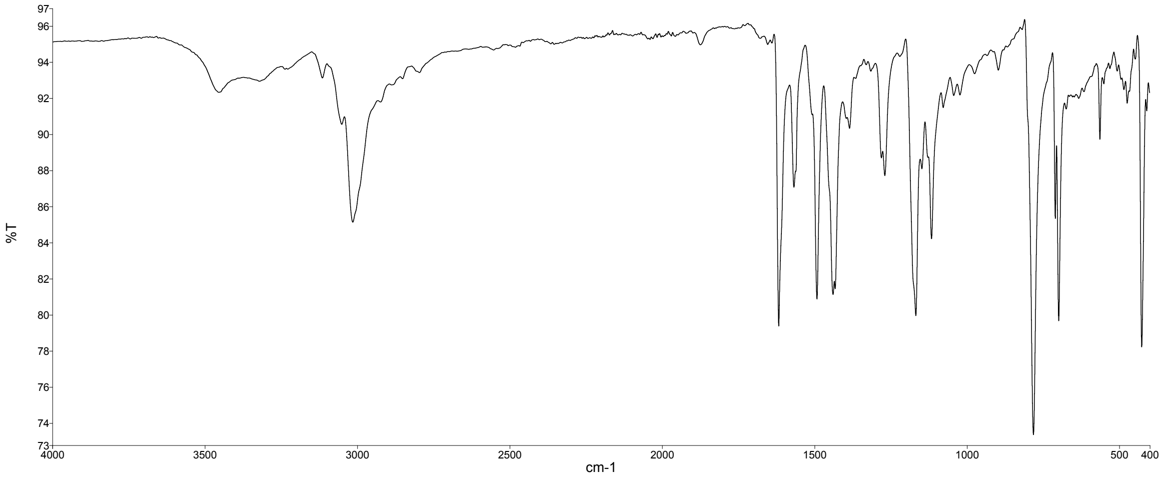

Figure S15. IR spectrum (ATR) of ***N*-Me-2ClPy**$^+$ I$^-$.

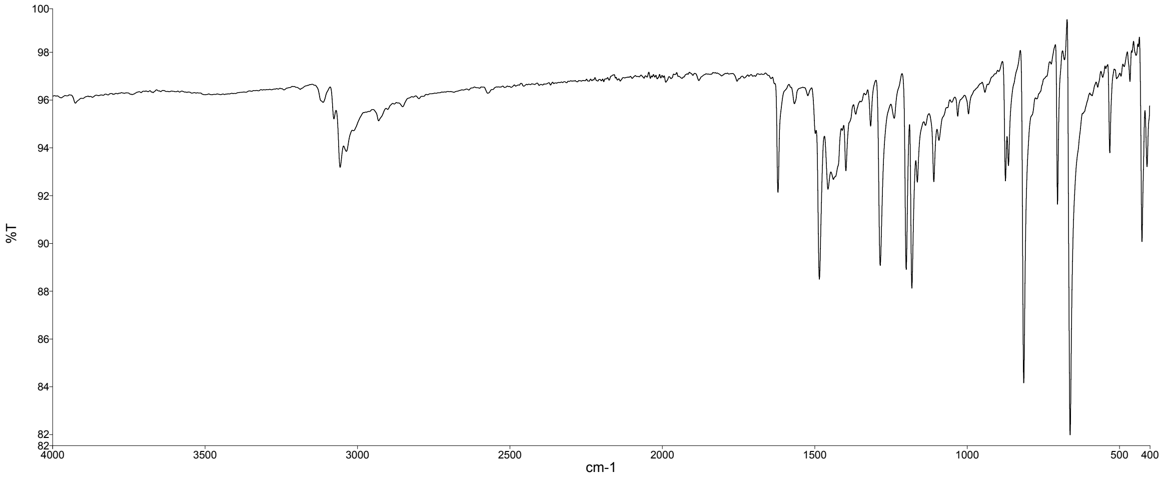

Figure S16. IR spectrum (ATR) of ***N*-Me-3ClPy**$^+$ I$^-$.

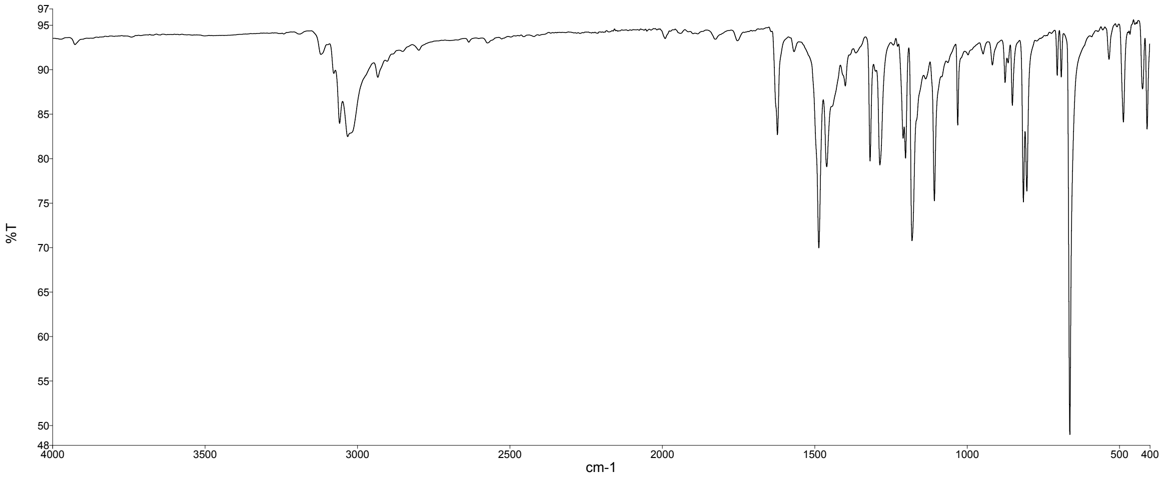

Figure S17. IR spectrum (ATR) of ***N*-Me-3BrPy**$^+$ I$^-$.

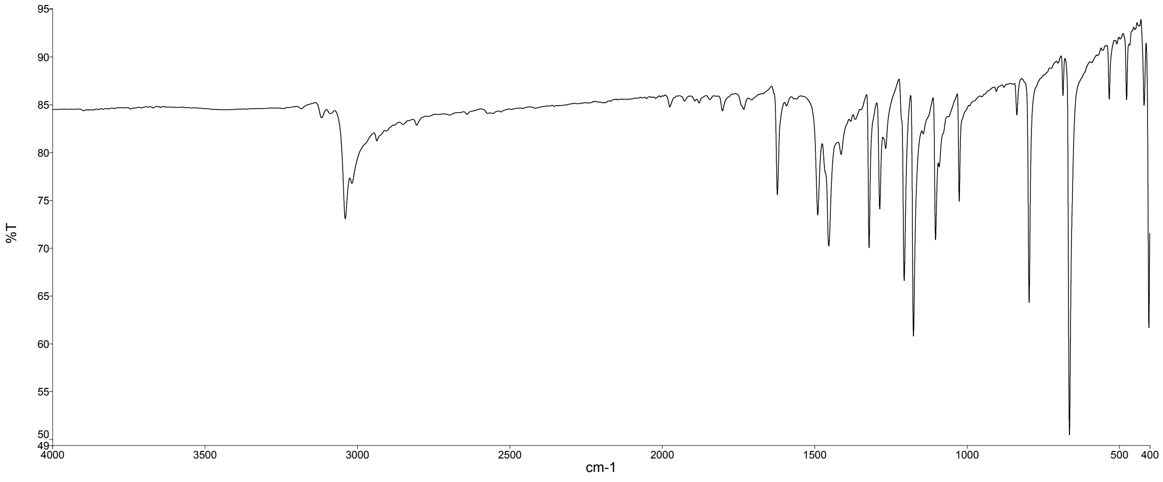

Figure S18. IR spectrum (ATR) of ***N*-Me-3IPy**$^+$ I$^-$.

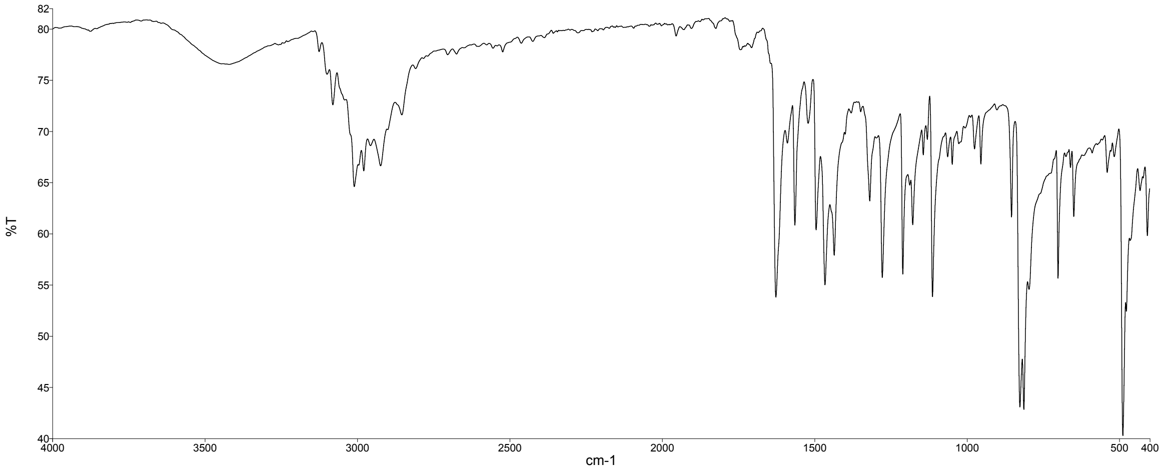

Figure S19. IR spectrum (ATR) of ***N*-Me-4ClPy**$^+$ I$^-$.

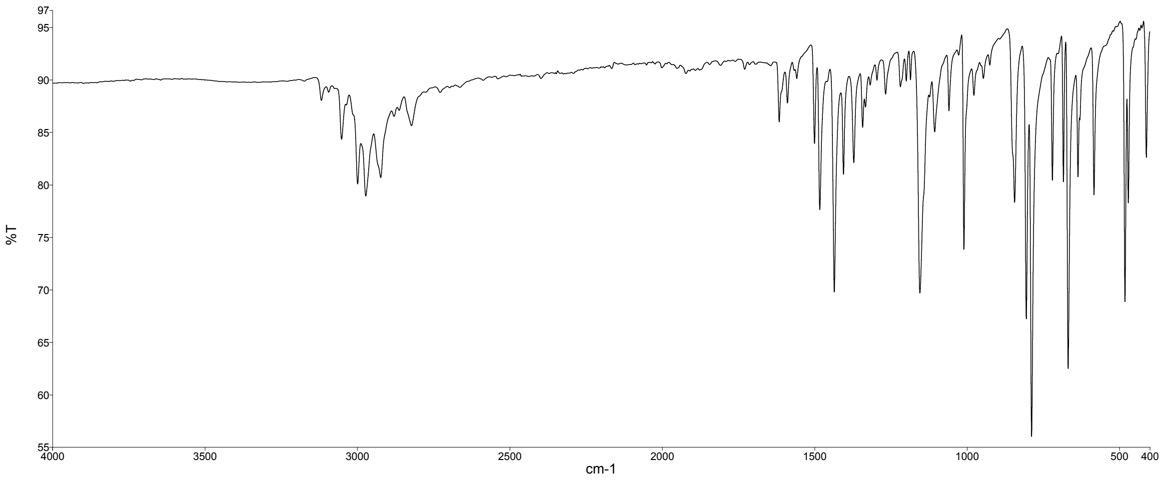

Figure S20. IR spectrum (ATR) of ***N*-Me-2IPy**$^+$ I$^-$.

Total electron energies and Cartesian coordinates for optimised structures of halogenopyridines:

2ClPy

E = −707.872254589 a.u.

C	-0.473106	-0.016668	-0.000038
C	0.196445	1.214165	0.000007
C	1.591133	1.182317	0.000220
C	2.243877	-0.055569	0.000331
C	1.462765	-1.212570	0.000201
N	0.119524	-1.198049	0.000044
H	2.155954	2.111242	0.000266
H	-0.356020	2.147907	-0.000084
H	3.327696	-0.125586	0.000518
H	1.925968	-2.197817	0.000328
Cl	-2.236291	-0.012909	-0.000333

2BrPy

E = −2821.39607377 a.u.

C	0.135117	-0.015109	-0.000015
C	0.803562	1.215746	0.000097
C	2.198672	1.181012	0.000297
C	2.849986	-0.057874	0.000417
C	2.068669	-1.214450	0.000357
N	0.724365	-1.195620	0.000116
H	2.765672	2.108732	0.000358
H	0.253828	2.150742	0.000012
H	3.933901	-0.128246	0.000562
H	2.529659	-2.200699	0.000405
Br	-1.796847	-0.006204	-0.000259

2IPy

E = - −7167.56484186 a.u.

C	0.600114	-0.012977	0.000075
C	1.275619	1.215343	0.000170
C	2.671206	1.178980	0.000369
C	3.320652	-0.060189	0.000483
C	2.537407	-1.215745	0.000415
N	1.193438	-1.193754	0.000198
H	3.239114	2.106260	0.000424
H	0.732615	2.154336	0.000083
H	4.404564	-0.132185	0.000629
H	2.996952	-2.202719	0.000475
I	-1.550138	-0.003809	-0.000228

3ClPy

E= −707.868646440 a.u.

C	0.208287	-1.193463	-0.000112
C	-0.493484	0.017753	-0.000058
C	0.209867	1.222606	0.000170
C	1.605284	1.159548	0.000316
C	2.223773	-0.094788	0.000200
N	1.545153	-1.250838	0.000015
H	-0.316131	2.173040	0.000227
H	2.201617	2.067969	0.000510
H	3.309643	-0.175590	0.000341
H	-0.329765	-2.139878	-0.000292
Br	-2.247282	0.009434	-0.000235

3BrPy

E = −2821.39344734 a.u.

C	0.815562	-1.194502	-0.000051
C	0.113467	0.015942	0.000033
C	0.815038	1.221686	0.000252
C	2.210723	1.160824	0.000400
C	2.830424	-0.092798	0.000338
N	2.153520	-1.249627	0.000093
H	0.291786	2.173219	0.000331
H	2.806057	2.069709	0.000557
H	3.916144	-0.173185	0.000407
H	0.283589	-2.144333	-0.000209
Br	-1.802386	0.004430	-0.000216

3IPy

E = −7167.56356653 a.u.

C	1.282614	-1.193146	0.000019
C	0.575427	0.016464	0.000095
C	1.284106	1.220515	0.000322
C	2.679735	1.160305	0.000475
C	3.298735	-0.093147	0.000410
N	2.620652	-1.249062	0.000171
H	0.768678	2.176250	0.000401
H	3.275024	2.069248	0.000633
H	4.384399	-0.174482	0.000488
H	0.756793	-2.146488	-0.000140
I	-1.551946	0.002886	-0.000198

4ClPy

E = −707.869825485 a.u.

C	1.596598	-1.142177	0.000021
C	0.198564	-1.205814	0.000007
C	-0.502861	0.000027	-0.000018
C	0.198568	1.205825	-0.000003
C	1.596639	1.142140	0.000033
N	2.297730	-0.000005	0.000032
H	-0.316634	2.161218	-0.000015
H	2.176622	2.064093	0.000026
H	2.176594	-2.064100	0.000041
H	-0.316693	-2.161162	0.000008
Cl	-2.254650	-0.000001	-0.000031

4BrPy

E= −2821.39433763 a.u.

C	2.205344	-1.142101	0.000024
C	0.806695	-1.205948	0.000017
C	0.106492	0.000019	-0.000009
C	0.806699	1.205951	0.000007
C	2.205373	1.142075	0.000046
N	2.906184	-0.000003	0.000036
H	0.295201	2.163068	-0.000006
H	2.785270	2.064167	0.000031
H	2.785247	-2.064168	0.000045
H	0.295154	-2.163023	0.000022
Br	-1.808222	0.000000	-0.000024

4IPy

E= −7167.56389206 a.u.

C	2.675101	1.141605	-0.000004
C	1.276627	1.204061	0.000018
C	0.569511	-0.000223	0.000061
C	1.276794	-1.204272	0.000028
C	2.675375	-1.141378	-0.000028
N	3.377002	0.000067	-0.000021
H	0.773147	-2.165451	0.000056
H	3.255109	-2.063440	-0.000012
H	3.255090	2.063989	-0.000034
H	0.772523	2.165529	0.000033
I	-1.557270	0.000003	-0.000007

Total electron energies and Cartesian coordinates for optimised structures of protonated halogenopyridines:

2ClPyH

E = −708.224428954 a.u.

C	1.598438	1.193956	-0.000003
C	0.203348	1.257312	0.000000
C	-0.523228	0.069484	0.000011
C	1.499370	-1.201345	-0.000008
C	2.257043	-0.045759	0.000002
H	2.173827	2.115705	-0.000001
H	-0.326209	2.203985	-0.000009
H	-0.406466	-1.974687	0.000002
H	1.918050	-2.202021	-0.000004
H	3.339303	-0.116097	0.000011
N	0.143363	-1.114294	0.000002
Cl	-2.230110	0.007723	-0.000002

2BrPyH

E = −2821.75015538 a.u.

C	2.195962	1.193985	-0.000005
C	0.800246	1.256399	0.000017
C	0.072486	0.068992	0.000016
C	2.096413	-1.201013	-0.000005
C	2.854905	-0.045485	-0.000012
H	2.771242	2.115849	-0.000010
H	0.274401	2.204859	-0.000006
H	0.192888	-1.974448	0.000006
H	2.514664	-2.201962	0.000000
H	3.937195	-0.115873	-0.000009
Br	-1.799866	0.003650	-0.000004

N 0.740690 -1.113351 0.000012

2IPyH

E = −7167.92220824 a.u.

C 2.663882 1.194580 -0.000002

C 1.267931 1.251889 0.000004

C 0.532589 0.066470 0.000007

C 2.567984 -1.198223 -0.000005

C 3.326053 -0.042546 -0.000002

H 3.236376 2.118246 -0.000002

H 0.748424 2.203678 -0.000007

H 0.674091 -1.978498 0.000000

H 2.988008 -2.198479 0.000000

H 4.408441 -0.111351 0.000004

N 1.212614 -1.112379 0.000004

I -1.560269 0.002265 -0.000001

3ClpyH

E = −708.223210732 a.u.

C -0.551749 0.026721 -0.000003

C 0.158341 -1.171312 -0.000019

C 2.229772 0.012671 -0.000004

C 1.555458 1.225851 -0.000006

C 0.158052 1.238905 0.000006

H -0.316234 -2.146762 -0.000012

H 2.012026 -2.021770 0.000034

H 3.309955 -0.081821 -0.000004

H 2.122434 2.150809 -0.000001

H -0.383985 2.180748 0.000009

Cl -2.271624 -0.008577 0.000002

N 1.510594 -1.133203 0.000013

3BrPyH

E = −2821.74901562 a.u.

C 0.053482 0.030758 -0.000001

C 0.760328 -1.168185 -0.000012

C 2.836244 0.008388 -0.000005

C 2.165551 1.223380 -0.000004

C 0.767490 1.240191 0.000006

H 0.287241 -2.144125 -0.000006

H 2.611971 -2.025540 0.000027

H 3.916186 -0.089192 -0.000010

H 2.735448 2.146633 0.000001

H 0.232561 2.185823 0.000011

N 2.113467 -1.135427 0.000010

Br -1.830750 -0.003795 0.000000

3IPyH

E = −7167.92096600 a.u.

C 1.244109 1.237873 -0.000003

C 0.519239 0.032103 -0.000010

C 1.232547 -1.164311 0.000005

C 3.312277 0.006162 -0.000005

C 2.642574 1.221219 0.000004

H 0.718824 2.188786 0.000004

H 0.766864 -2.143761 0.000006

H 3.082682 -2.026902 -0.000003

H 4.392028 -0.093479 0.000008

H 3.212902 2.144258 0.000015

I -1.584591 -0.002219 0.000000

N 2.586505 -1.135650 0.000001

4ClPyH

E= −708.228175221 a.u.

C -0.560968 -0.000002 -0.000002

C 0.144168 1.217774 0.000002

C 1.525586 1.189358 0.000007

C 1.525588 -1.189356 0.000001

C 0.144168 -1.217774 -0.000002

H -0.375948 2.169768 0.000003

H 2.136614 2.085655 0.000010

H 3.195995 0.000001 0.000008

H 2.136614 -2.085654 0.000001

H -0.375943 -2.169771 -0.000005

N 2.176946 0.000000 0.000006

Cl-2.272188 0.000000 -0.000005

4BrPyH

E = −2821.75349269 a.u.

C 0.044326 -0.000009 0.000000

C 0.749861 1.216833 0.000005

C 2.131951 1.189199 0.000009

C 2.131962 -1.189189 0.000002

C 0.749864 -1.216837 0.000000

C 0.234947 2.171429 0.000004

H 2.742689 2.085709 0.000012

H 3.802861 0.000008 0.000009

H	2.742694	-2.085703	0.000002
H	0.234970	-2.171444	-0.000002
N	2.783891	0.000001	0.000007
Br	-1.831234	0.000000	-0.000005

4IPyH

E = −7167.92471437 a.u.

C	0.508855	0.000031	0.000003
C	1.223720	-1.214197	0.000006
C	2.606001	-1.188418	0.000004
C	2.606040	1.188384	-0.000004
C	1.223730	1.214209	0.000006
H	0.717032	-2.173096	0.000000
H	3.216068	-2.085428	-0.000007
H	4.277988	-0.000032	-0.000009
H	3.216079	2.085416	0.000001
H	0.717119	2.173148	0.000019
N	3.259210	-0.000005	-0.000006
I	-1.584318	-0.000001	-0.000001

Total electron energies and Cartesian coordinates for optimised structures of *N*-methylated halogenopyridines:

2ClPyMe

E = −747.543238053 a.u.

C	-1.897337	-1.225941	0.000016
C	-0.527758	-1.479202	0.000231
C	0.368136	-0.411371	0.000460
C	-1.425594	1.123032	-0.000093
C	-2.352026	0.098520	-0.000227
H	-2.601337	-2.053314	-0.000262
H	-0.134756	-2.490115	0.000220
H	-1.716604	2.166923	-0.000221
H	-3.409280	0.341188	-0.000673
C	0.864262	2.023246	0.000006
H	1.488724	1.979630	0.893111
H	1.489137	1.978807	-0.892792
H	0.286458	2.944947	-0.000325
N	-0.084900	0.875747	0.000303
Cl	2.059639	-0.692235	-0.000208

2BrPyMe

E = −2861.06803401 a.u.

C	-2.172838	-1.469921	0.000046
C	-0.779726	-1.473644	0.000090
C	-0.086451	-0.263985	-0.000110
C	-2.126842	0.924868	-0.000050
C	-2.856401	-0.248094	-0.000073
H	-2.717692	-2.409817	0.000140
H	-0.216879	-2.400523	0.000313
H	-2.600078	1.899745	0.000030

H	-3.939988	-0.197394	-0.000152
C	-0.042987	2.223783	0.000082
H	0.579031	2.295579	0.892956
H	0.578969	2.295715	-0.892800
H	-0.780360	3.023726	0.000272
N	-0.763106	0.921007	-0.000017
Br	1.795149	-0.260346	-0.000016

2IPyMe

E = −7207.23883537 a.u.

C	-2.524911	-1.546461	0.000022
C	-1.133775	-1.470631	0.000039
C	-0.497569	-0.226908	0.000024
C	-2.609893	0.844757	-0.000074
C	-3.275484	-0.365451	-0.000035
H	-3.015656	-2.515741	-0.000015
H	-0.528742	-2.370413	0.000103
H	-3.136949	1.791554	-0.000111
H	-4.360245	-0.373542	-0.000105
C	-0.614529	2.261943	0.000019
H	0.001047	2.374342	0.893347
H	0.001805	2.373839	-0.892817
H	-1.399705	3.015257	-0.000487
N	-1.248121	0.916238	0.000104
I	1.605892	-0.145141	-0.000012

3ClPyMe

E = −747.544118791 a.u.

C	-0.640119	1.433450	0.001913
C	-0.894357	0.053689	-0.000102
C	0.176824	-0.834262	-0.003414
C	1.719705	0.950647	-0.002229
C	0.682332	1.874969	0.001007
H	-1.464860	2.140631	0.003547
H	2.763620	1.241891	-0.002945
H	0.918588	2.933941	0.002011
C	2.561349	-1.370670	0.004490
H	2.538897	-1.921311	0.946426
H	2.435056	-2.053675	-0.836522
H	3.510750	-0.847399	-0.092064
N	1.452553	-0.376160	-0.004862
H	0.038092	-1.910260	-0.004904
Cl	-2.502495	-0.564567	0.000500

3BrPyMe

E = −2861.06976208 a.u.

C	0.125530	1.571685	0.004710
C	-0.278581	0.229044	-0.000546
C	0.688849	-0.770428	-0.008992
C	2.418779	0.834596	-0.005554
C	1.489286	1.866432	0.002685
H	-0.612972	2.368072	0.008883
H	3.488520	1.009017	-0.007052
H	1.840068	2.893252	0.005401
C	3.005715	-1.560721	0.011616

H	3.047423	-1.982609	1.017624
H	2.708919	-2.325379	-0.706402
H	3.981726	-1.165847	-0.265192
N	2.007865	-0.455490	-0.012523
H	0.436556	-1.825269	-0.012888
Br	-2.104079	-0.251613	0.000679

3IPyMe

E = −7207.24151769 a.u.

C	0.679691	1.611482	0.005022
C	0.207734	0.288823	-0.000999
C	1.136939	-0.747910	-0.010235
C	2.938297	0.777549	-0.005928
C	2.054943	1.848380	0.003083
H	-0.016190	2.445323	0.009890
H	4.014801	0.904190	-0.007204
H	2.449323	2.859317	0.006389
C	3.418383	-1.639706	0.013024
H	3.466647	-2.040630	1.027338
H	3.071821	-2.406787	-0.679474
H	4.403673	-1.295023	-0.296419
N	2.469656	-0.492759	-0.013955
H	0.847042	-1.793093	-0.014624
I	-1.851710	-0.151994	0.000528

4ClPyMe

E = −747.548473088 a.u.

C	-1.031210	0.000070	0.000025
C	-0.319568	-1.210382	-0.003391
C	1.062424	-1.175900	-0.011158
C	1.062075	1.176745	-0.011139
C	0.320132	1.210641	-0.003372
H	1.658009	2.082900	-0.013012
H	-0.832611	2.166890	-0.002470
C	3.225944	-0.000685	0.021970
H	3.559816	-0.024146	1.061487
H	3.597184	-0.876414	-0.509435
H	3.597705	0.898084	-0.468729
N	1.739570	0.000634	-0.017327
H	1.659071	-2.081639	-0.013031
H	-0.831755	-2.166762	-0.002511
Cl	-2.744800	-0.000370	0.006552

4BrPyMe

E = −2861.07370136 a.u.

C	-0.382977	0.000193	-0.001902
C	0.328980	-1.209562	-0.004720
C	1.711536	-1.175845	-0.011359
C	1.711324	1.176692	-0.011340
C	0.328556	1.209983	-0.004700
H	2.307429	2.082791	-0.012788
H	-0.179508	2.168343	-0.004186
C	3.874931	-0.000802	0.023396
H	4.208227	-0.024155	1.063133

H	4.246646	-0.876656	-0.507504
H	4.247231	0.897914	-0.467029
N	2.388993	0.000597	-0.017007
H	2.308247	-2.081596	-0.012809
H	-0.178903	-2.167996	-0.004229
Br	-2.260469	-0.000194	0.003663

4IPyMe

E = −7207.24479840 a.u.

C	0.119679	0.000250	-0.002709
C	0.840468	-1.206774	-0.005209
C	2.223395	-1.175045	-0.011354
C	2.223254	1.175883	-0.011334
C	0.840116	1.207264	-0.005188
H	2.818704	2.082471	-0.012617
H	0.340727	2.170139	-0.004811
C	4.387239	-0.000865	0.024192
H	4.720609	-0.024246	1.063970
H	4.759428	-0.876760	-0.506405
H	4.760026	0.897882	-0.465909
N	2.902082	0.000573	-0.016828
H	2.819400	-2.081322	-0.012639
H	0.341207	-2.169698	-0.004856
I	-1.975086	-0.000127	0.002466

Total electron energies and Cartesian coordinates for optimised structures of *N*-methylatedhalogenopyridinium cation complexes with pyridine:

py

E = −248.18841213 a.u.

Symbol	X	Y	Z
C	-0.0000440	-1.3837990	-0.0000020
C	1.1973180	-0.6721800	0.0000020
C	1.1408380	0.7216160	0.0000370
C	-1.1407870	0.7216880	-0.0000310
C	-1.1973530	-0.6721230	-0.0000060
H	-0.0000630	-2.4697170	-0.0000040
H	2.1559900	-1.1805040	-0.0000090
H	2.0567760	1.3084890	0.0000090
H	-2.0567070	1.3085940	-0.0000050
H	-2.1560650	-1.1803720	0.0000010
N	0.0000350	1.4174710	0.0000010

(**2BrpyMe**)(**py**)

E = −3109.14634090 a.u.

Symbol	X	Y	Z
C	-4.0521450	-1.5727670	-0.0001940
H	-4.5414080	-2.5414200	-0.0003210
C	-2.6676170	-1.4944070	-0.0002430
H	-2.0482440	-2.3835700	-0.0004140
C	-2.0426350	-0.2501680	-0.0000640
C	-4.1492520	0.8110030	0.0002040
H	-4.6743560	1.7584800	0.0003690
N	2.5818620	-0.0325380	-0.0000710

C	5.3740720	-0.0409880	0.0001360
H	6.4593410	-0.0447250	0.0002180
C	3.2731170	-0.0350130	-1.1465010
H	2.6863520	-0.0352790	-2.0621260
C	4.6647030	-0.0388450	-1.1984770
H	5.1733250	-0.0412790	-2.1561540
C	4.6645230	-0.0396090	1.1986440
H	5.1730020	-0.0426130	2.1563960
C	3.2729460	-0.0357330	1.1464600
H	2.6860460	-0.0366050	2.0619970
C	-4.8074830	-0.3973610	0.0000230
H	-5.8905760	-0.4109310	0.0000620
C	-2.1443830	2.2144610	0.0003330
H	-1.5265050	2.3103060	0.8919990
H	-1.5267830	2.3106300	-0.8914910
H	-2.9230630	2.9726080	0.0005990
N	-2.7931780	0.8810290	0.0001750
Br	-0.1680860	-0.1279570	-0.0001080

(**2ClpyMe**)(**py**)

E = −995.57839581 a.u.

Symbol	X	Y	Z
C	-4.0100120	-1.5830910	-0.0003130
H	-4.5080690	-2.5471800	-0.0004930
C	-2.6252990	-1.5193840	-0.0003280
H	-2.0098380	-2.4115360	-0.0005130
C	-1.9934570	-0.2791540	-0.0000950
C	-4.0868530	0.8022410	0.0001880
H	-4.6028260	1.7547630	0.0003930

N	2.4877890	-0.0347890	-0.0000330
C	5.2845730	-0.0782580	0.0000210
H	6.3697510	-0.0955340	0.0000410
C	3.1823280	-0.0460250	-1.1442790
H	2.5977050	-0.0391140	-2.0614500
C	4.5742630	-0.0672550	-1.1979030
H	5.0823360	-0.0761960	-2.1559320
C	4.5742020	-0.0682210	1.1979180
H	5.0822250	-0.0779100	2.1559660
C	3.1822700	-0.0469400	1.1442380
H	2.5975990	-0.0407710	2.0613840
C	-4.7548020	-0.4004750	-0.0000570
H	-5.8379440	-0.4047510	-0.0000390
C	-2.0599710	2.1842890	0.0004180
H	-1.4411770	2.2713290	0.8923940
H	-1.4412700	2.2716910	-0.8915850
H	-2.8282070	2.9528130	0.0006150
N	-2.7303150	0.8607350	0.0001760
Cl	-0.2921210	-0.1603280	-0.0000370

(**2IpyMe**)(**py**)

E = −7455.70706021 a.u.

Symbol	X	Y	Z
I	-0.0368070	-0.0983680	-0.0000840
C	-4.1609340	-1.5834380	0.0000460
H	-4.6322300	-2.5610470	0.0000450
C	-2.7777130	-1.4765270	-0.0000140
H	-2.1508840	-2.3608000	-0.0000580

C	-2.1616980	-0.2250050	-0.0000140
C	-4.2979090	0.7961120	0.0000970
H	-4.8402760	1.7338010	0.0001380
N	2.6594570	-0.0315260	-0.0000580
C	5.4424780	-0.0142780	0.0001650
H	6.5276590	-0.0080530	0.0002520
C	3.3442140	-0.0277120	-1.1506560
H	2.7533240	-0.0332170	-2.0634460
C	4.7348070	-0.0188080	-1.1996650
H	5.2441340	-0.0164970	-2.1567720
C	4.7346140	-0.0188730	1.1998830
H	5.2437870	-0.0166190	2.1570710
C	3.3440300	-0.0277680	1.1506510
H	2.7529930	-0.0333180	2.0633460
C	-4.9372370	-0.4226290	0.0001070
H	-6.0199920	-0.4539790	0.0001580
C	-2.3293360	2.2357480	0.0000300
H	-1.7135220	2.3464690	0.8916420
H	-1.7134660	2.3464420	-0.8915460
H	-3.1234380	2.9779950	-0.0000120
N	-2.9436300	0.8874420	0.0000340

(**3BrpyMe**)(**py**)

E = −3109.14364832 a.u.

Symbol	X	Y	Z
C	3.8516570	-1.6702970	0.0001510
H	4.3607060	-2.6269800	0.0002370
C	2.4635430	-1.5897000	0.0000950

H	1.8573770	-2.4903510	0.0001330
C	1.8451200	-0.3351380	-0.0000110
N	-2.8093180	0.0809490	-0.0001260
C	-5.6005310	0.1901350	0.0002280
H	-6.6850450	0.2321740	0.0003660
C	-3.5002330	0.1081980	1.1456040
H	-2.9131640	0.0850240	2.0607820
C	-4.8911100	0.1627140	1.1984850
H	-5.3989860	0.1829490	2.1564270
C	-4.8914190	0.1625950	-1.1982090
H	-5.3995440	0.1827300	-2.1560210
C	-3.5005280	0.1080860	-1.1456820
H	-2.9136950	0.0848220	-2.0610080
C	4.6033270	-0.5070180	0.0000990
H	5.6865560	-0.5048130	0.0001360
C	2.6460020	0.7961760	-0.0000570
H	2.2350500	1.8007540	-0.0001410
C	4.7907100	1.9441920	-0.0000830
H	4.5488130	2.5153180	-0.8952560
H	4.5485720	2.5155880	0.8948510
H	5.8473340	1.6890810	0.0001010
N	3.9931480	0.6940520	-0.0000020
Br	-0.0274290	-0.1517420	-0.0000980

(**3ClpyMe**)(**py**)

E = −995.57516453 a.u.

Symbol	X	Y	Z
C	3.7531740	-1.6999980	0.0002330

H	4.2545230	-2.6606710	0.0003750
C	2.3666040	-1.6105970	0.0001760
H	1.7499460	-2.5042980	0.0002690
C	1.7594110	-0.3502680	-0.0000030
N	-2.7497100	0.0622870	-0.0001590
C	-5.5456550	0.1599820	0.0001970
H	-6.6303680	0.1977580	0.0003360
C	-3.4437210	0.0867860	1.1435930
H	-2.8584250	0.0664900	2.0601640
C	-4.8352790	0.1354390	1.1978900
H	-5.3428980	0.1536880	2.1561030
C	-4.8355920	0.1352490	-1.1976760
H	-5.3434610	0.1533410	-2.1557600
C	-3.4440180	0.0866050	-1.1437350
H	-2.8589600	0.0661620	-2.0604550
C	4.5138110	-0.5420310	0.0001110
H	5.5969810	-0.5479630	0.0001460
C	2.5675340	0.7769480	-0.0001160
H	2.1584210	1.7823620	-0.0002590
C	4.7194400	1.9082340	-0.0002090
H	4.4812560	2.4805580	-0.8955930
H	4.4810600	2.4809030	0.8949000
H	5.7740330	1.6450660	-0.0000410
N	3.9125880	0.6633120	-0.0000590
Cl	0.0555000	-0.1712170	-0.0000830

(**3IpyMe**)(**py**)

E = −7455.70458390 a.u.

Symbol	X	Y	Z
I	0.1191020	-0.1379370	0.0001180
C	-4.0205450	-1.6451080	-0.0001990
H	-4.5381150	-2.5973060	-0.0002930
C	-2.6307650	-1.5737230	-0.0001360
H	-2.0405230	-2.4852890	-0.0001730
C	-1.9912340	-0.3277130	-0.0000250
N	2.8682530	0.0972250	0.0001420
C	5.6511420	0.2167750	-0.0002830
H	6.7354560	0.2623820	-0.0004490
C	3.5535000	0.1272020	-1.1491590
H	2.9629550	0.1015990	-2.0619560
C	4.9433280	0.1871000	-1.1996110
H	5.4519360	0.2092340	-2.1569420
C	4.9437040	0.1868820	1.1992600
H	5.4526140	0.2088260	2.1564350
C	3.5538590	0.1269950	1.1492350
H	2.9636000	0.1012330	2.0622120
C	-4.7616490	-0.4759300	-0.0001460
H	-5.8448730	-0.4632510	-0.0001840
C	-2.7899760	0.8055830	0.0000100
H	-2.3787520	1.8103870	0.0000950
C	-4.9244750	1.9760630	0.0000670
H	-4.6776530	2.5455040	0.8950160
H	-4.6771840	2.5459600	-0.8944590
H	-5.9838260	1.7321020	-0.0002790

N	-4.1395970	0.7191360	-0.0000480

(**4BrpyMe**)(**py**)

E = −3109.14713478 a.u.

Symbol	X	Y	Z
C	-3.8525800	-1.1738110	0.0096720
H	-4.4536400	-2.0756650	0.0215930
C	-2.4738980	-1.2068820	0.0154850
H	-1.9639290	-2.1632970	0.0296880
C	-1.7588460	-0.0039620	0.0000690
C	-2.4722590	1.2020340	-0.0185650
H	-1.9595910	2.1570830	-0.0322700
C	-3.8491940	1.1709140	-0.0241540
H	-4.4519380	2.0722970	-0.0386440
N	2.9235070	0.0003400	-0.0017900
C	5.7178600	0.0053890	0.0032290
H	6.8029840	0.0072510	0.0052030
C	-6.0040630	0.0161120	0.0173950
H	-6.3745850	-0.9606990	-0.2843340
H	-6.3381450	0.2466760	1.0286920
H	-6.3621580	0.7684990	-0.6828670
N	-4.5239140	-0.0017360	-0.0124310
C	3.6180100	-0.0148350	-1.1458740
H	3.0332850	-0.0297070	-2.0626450
C	5.0099190	-0.0132700	-1.1957810
H	5.5194080	-0.0269070	-2.1528380
C	5.0056030	0.0216000	1.1997300
H	5.5113980	0.0368780	2.1587130

C	3.6138960	0.0180680	1.1447200
H	3.0260110	0.0305730	2.0595120
Br	0.1110590	-0.0051820	0.0004050

(**4ClpyMe**)(**py**)

E = −995.57948367 a.u.

Symbol	X	Y	Z
C	-3.7421310	-0.9313140	0.7126350
H	-4.3427290	-1.6429870	1.2681170
C	-2.3654160	-0.9544980	0.7346560
H	-1.8483260	-1.7078080	1.3182860
C	-1.6543070	-0.0010500	-0.0060710
C	-2.3684610	0.9488280	-0.7473440
H	-1.8541930	1.7026890	-1.3327240
C	-3.7457110	0.9186220	-0.7284800
H	-4.3477340	1.6301690	-1.2821860
N	2.8674050	0.0063350	-0.0006450
C	5.6652990	-0.0039160	0.0060860
H	6.7506400	-0.0078860	0.0086970
C	-5.8971810	0.0128460	0.0300580
H	-6.2634190	-1.0091980	0.1021860
H	-6.2217990	0.5938250	0.8930490
H	-6.2688820	0.4623630	-0.8880810
N	-4.4168770	-0.0094420	-0.0107670
C	3.5616020	-0.7510270	-0.8581320
H	2.9757710	-1.3539200	-1.5484530
C	4.9538440	-0.7914980	-0.8955780
H	5.4609660	-1.4250500	-1.6149950

C	4.9552940	0.7888960	0.9043060
H	5.4636570	1.4187050	1.6261270
C	3.5630340	0.7586390	0.8601560
H	2.9782270	1.3658700	1.5475110
Cl	0.0475840	0.0016350	-0.0053760

(**4ClpyMe**)(**py**)

E = −7455.70681042 a.u.

Symbol	X	Y	Z
I	0.1962060	0.0025650	0.0017070
C	-4.0127730	-0.9152020	0.7362380
H	-4.6132920	-1.6149960	1.3065890
C	-2.6335300	-0.9318110	0.7593300
H	-2.1294980	-1.6755360	1.3665810
C	-1.9115370	-0.0004720	0.0008560
C	-2.6360630	0.9271680	-0.7614250
H	-2.1334010	1.6725410	-1.3678400
C	-4.0142900	0.8984370	-0.7486560
H	-4.6183750	1.5939490	-1.3210380
N	2.9662510	0.0022340	0.0001300
C	5.7525610	-0.0034620	-0.0016080
H	6.8378480	-0.0057370	-0.0022440
C	-6.1662240	0.0146280	0.0117590
H	-6.5337500	-0.9699900	0.2920050
H	-6.5001770	0.7592180	0.7342880
H	-6.5301470	0.2634860	-0.9832450
N	-4.6870390	-0.0134140	-0.0107560

C	3.6510200	-0.7721400	-0.8502360
H	3.0587860	-1.3855070	-1.5253300
C	5.0419180	-0.8084080	-0.8887780
H	5.5491210	-1.4526930	-1.5982120
C	5.0463400	0.8043920	0.8864360
H	5.5570200	1.4465000	1.5953490
C	3.6552520	0.7738080	0.8496480
H	3.0664180	1.3894390	1.5256340

Table S4. Molecular electrostatic potentials (*MEP*s) on halogen, nitrogen and carbon atoms in neutral halogenated pyridines. All values are in kJ mol^{-1} e^{-1}.

pyridine	p*K*a	*MEP*(**X**)σ	*MEP*(**X**)	*MEP*(**N**)	*MEP*(**C1**)	*MEP*(**C2**)	*MEP*(**C3**)	*MEP*(**C4**)	*MEP*(**C5**)
**2ClPy**	1.1	26.5	-51.4	-40.4	8.0	-2.6	8.2	-1.7	2.7
**2BrPy**	1.5	61.3	-57.9	-35.8	8.0	-0.9	7.3	-2.1	2.0
**2IPy**	1.7	94.8	-53.6	-38.0	5.1	-2.5	6.6	1.3	4.5
**3ClPy**	3.3	50.7	-31.9	-38.6	4.7	5.6	3.3	-5.1	-6.9
**3BrPy**	3.5	89.1	-35.1	-38.5	1.4	4.1	7.3	-3.1	0.4
**3IPy**	3.5	122.0	-31.2	-36.6	1.9	1.0	5.7	-3.2	1.2
**4ClPy**	4.0	58.0	-22.6	-48.2	0.8	-3.6	10.3	-3.6	0.8
**4BrPy**	4.0	95.4	-26.6	-38.6	0.2	-3.1	10.0	-3.1	0.2
**4IPy**	4.0	128.5	-23.9	-44.2	3.5	-3.9	6.6	-3.9	3.5

Table S5. Molecular electrostatic potentials (*MEP*s) on hydrogen atoms in neutral halogenated pyridines. All values are in kJ mol^{-1} e^{-1}.

Pyridine	pK_a	*MEP*(**H1**)	*MEP*(**H2**)	*MEP*(**H3**)	*MEP*(**H4**)	*MEP*(**H5**)
**2ClPy**	1.1	-	122.8	140.1	129.7	104.6
**2BrPy**	1.5	-	121.4	140.3	131.5	106.6
**2Ipy**	1.7	-	121.3	139.5	131.7	104.5
**3ClPy**	3.3	94.3	-	133.4	132.2	99.3
**3BrPy**	3.5	93.2	-	131.6	132.0	102.0
**3Ipy**	3.5	91.8	-	130.1	132.1	101.2
**4ClPy**	4.0	103.2	126.1	-	126.1	103.2
**4BrPy**	4.0	103.1	124.8	-	124.8	103.1
**4Ipy**	4.0	103.0	122.8	-	122.8	103.0

Table S6. Molecular electrostatic potentials (*MEP*s) on halogen, nitrogen and carbon atoms in halogenopyridinium cations. All values are in kJ mol⁻¹ e⁻¹.

cation	p*K*a	*MEP*(**X**)σ	*MEP*(**X**)	*MEP*(**N**)	*MEP*(**C1**)	*MEP*(**C2**)	*MEP*(**C3**)	*MEP*(**C4**)	*MEP*(**C5**)
**2ClPyH**	1.1	434.7	367.9	485.4	485.7	434.1	436.5	426.2	470.1
**2BrPyH**	1.5	481.9	347.4	478.5	477.2	427.0	429.7	420.4	462.9
**2IPyH**	1.7	511.3	337.5	466.4	457.9	415.7	419.4	409.1	449.0
**3ClPyH**	3.3	389.1	329.2	491.1	476.0	444.4	440.4	434.5	483.1
**3BrPyH**	3.5	437.9	314.6	486.0	483.8	436.6	436.8	429.3	476.9
**3IPyH**	3.5	468.9	299.9	478.4	473.1	424.1	424.3	420.6	466.0
**4ClPyH**	4.0	392.9	334.2	481.9	468.7	428.8	446.8	428.8	468.7
**4BrPyH**	4.0	438.9	323.1	477.1	463.7	422.8	438.2	422.8	463.7
**4IPyH**	4.0	470.0	309.9	468.6	458.6	416.8	422.4	416.8	458.6

Table S7. Molecular electrostatic potentials (*MEP*s) on hydrogen atoms in halogenopyridinium cations. All values are in kJ mol⁻¹ e⁻¹.

cation	pK_a	*MEP*(**H1**)	*MEP*(**H2**)	*MEP*(**H3**)	*MEP*(**H4**)	*MEP*(**H5**)	*MEP*(H_N)
**2ClPyH**	1.1	-	498.1	492.9	493.0	548.6	689.1
**2BrPyH**	1.5	-	492.0	487.9	490.6	539.7	677.7
**2IPyH**	1.7	-	485.3	478.9	481.0	533.3	665.5
**3ClPyH**	3.3	547.6	-	495.2	501.2	546.3	700.2
**3BrPyH**	3.5	541.4	-	487.5	496.6	546.3	693.0
**3IPyH**	3.5	532.9	-	480.7	489.7	535.1	688.0
**4ClPyH**	4.0	548.1	497.4	-	497.4	548.1	687.3
**4BrPyH**	4.0	542.8	491.5	-	491.5	542.8	688.8
**4IPyH**	4.0	533.2	485.2	-	485.2	533.2	677.1

Table S8. Molecular electrostatic potentials (*MEP*s) on halogen, nitrogen and carbon atoms in *N*-methylpyridinium cations. All values are in kJ mol^{-1} e^{-1}.

cation	*MEP*(**X**)σ	*MEP*(**X**)	*MEP*(**N**)	*MEP*(**C1**)	*MEP*(**C2**)	*MEP*(**C3**)	*MEP*(**C4**)	*MEP*(**C5**)
**2ClPyMe**	422.4	363.7	483.2	470.4	412.8	413.8	406.8	452.1
**2BrPyMe**	464.3	341.0	481.9	452.1	406.4	408.2	404.3	443.3
**2IPyMe**	492.3	333.3	468.0	439.4	396.9	399.8	394.1	431.1
**3ClPyMe**	378.4	324.7	489.8	462.5	423.1	416.5	413.5	448.4
**3BrPyMe**	420.2	300.6	494.8	455.6	418.1	413.7	409.1	454.2
**3IPyMe**	452.1	288.2	484.9	454.7	405.2	408.3	405.1	454.4
**4ClPyMe**	377.6	321.1	491.4	453.4	411.6	427.0	411.6	453.4
**4BrPyMe**	423.1	307.5	486.5	449.5	407.9	420.3	407.9	449.5
**4IPyMe**	454.4	291.0	460.5	436.8	399.4	402.2	397.6	436.5

Table S9. Molecular electrostatic potentials (*MEP*s) on hydrogen atoms in *N*-methylpyridinium cations. All values are in kJ mol^{-1} e^{-1}.

cation	*MEP*(**H1**)	*MEP*(**H2**)	*MEP*(**H3**)	*MEP*(**H4**)	*MEP*(**H5**)
**2ClPyMe**	-	480.6	478.0	478.0	534.6
**2BrPyMe**	-	477.0	470.9	477.5	531.9
**2IPyMe**	-	468.9	465.9	471.2	521.5
**3ClPyMe**	529.0	-	477.9	482.3	537.1
**3BrPyMe**	523.0	-	472.2	481.0	533.3
**3IPyMe**	519.6	-	465.0	473.9	528.0
**4ClPyMe**	534.6	484.1	-	484.1	534.6
**4BrPyMe**	531.4	477.3	-	477.3	531.4
**4IPyMe**	521.4	470.1	-	470.1	521.4

Table S10. Binding energies for the pyridine-methylpyridinium cation complexes

complex	E_{bind} / kJ mol^{-1}
(**2ClpyMe**)(**py**)	−40.5
(**2BrpyMe**)(**py**)	−51.4
(**2IpyMe**)(**py**)	−67.9
(**3ClpyMe**)(**py**)	−33.8
(**3BrpyMe**)(**py**)	−43.9
(**3IpyMe**)(**py**)	−58.8
(**4ClpyMe**)(**py**)	−33.9
(**4BrpyMe**)(**py**)	−43.4
(**4IpyMe**)(**py**)	−57.4

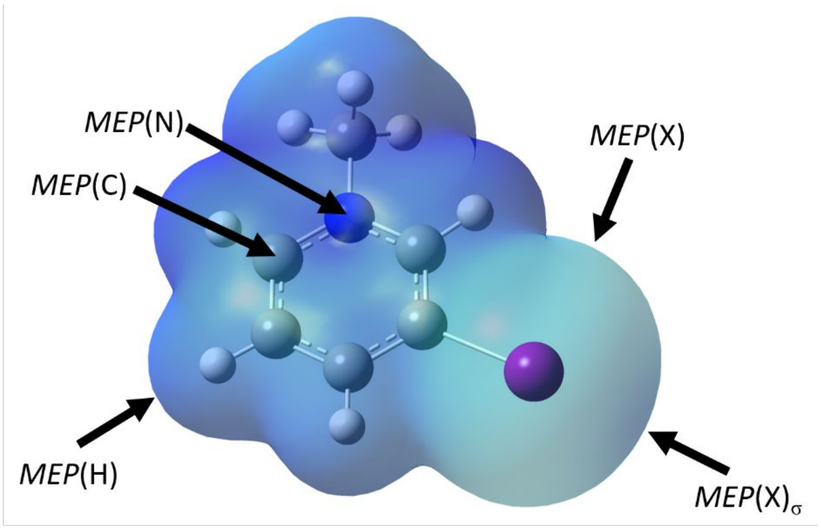

Figure S21. Positions in molecules and ions from which the values of molecular electrostatic potentials were used for an analysis.

Table S10. Results of CSD survay on halogen bonding of neutral halogenoheterocycles as halogen bond donors and oxygen, nitrogen, chloride, bromide and iodide as halogen bond acceptor.

		Number of strucutres	Number of strucutres with halogen bond	Precentage of structures with halogen bonds
Cl	*o*	1047	248	23,7%
	m	445	150	33,7%
	p	198	57	28,8%
	sum	1690	455	26,9%
Br	*o*	290	104	35,9%
	m	450	175	38,9%
	p	47	27	57,4%
	sum	787	306	38,9%
I	*o*	50	33	66,0%
	m	73	49	67,1%
	p	39	32	82,1%
	sum	162	114	70,4%

Table S11. Results of CSD survay on halogen bonding of protonated halogenoheterocycles as halogen bond donors and oxygen, nitrogen, chloride, bromide and iodide as halogen bond acceptor.

		Number of strucutres	Number of strucutres with halogen bond	Precentage of structures with halogen bonds
Cl	*o*	57	27	47,4%
	m	105	49	46,7%
	p	89	72	80,9%
		251	148	59,0%
Br	*o*	53	46	86,8%
	m	120	99	82,5%
	p	36	34	94,4%
		209	179	85,6%
I	*o*	12	11	91,7%
	m	37	30	81,1%
	p	20	18	90,0%
		69	59	85,5%

Table S12. Results of CSD survay on halogen bonding of *N*-methylated halogenoheterocycles as halogen bond donors and oxygen, nitrogen, chloride, bromide and iodide as halogen bond acceptor.

		Number of strucutres	Number of strucutres with halogen bond	Precentage of structures with halogen bonds
Cl	*o*	15	5	33,3%
	m	7	1	14,3%
	p	2	0	0,0%
		24	6	25,0%
Br	*o*	6	3	50,0%
	m	34	24	70,6%
	p	2	1	50,0%
		42	28	66,7%
I	*o*	2	2	100,0%
	m	55	52	94,5%
	p	3	2	66,7%
		60	56	93,3%

List of refcodes covered by the CSD survay

I. Neutral halogenoheterocycles

o-chloroheterocycles with a XB contact

ACAPAX	ADUPUN	AJIWEW	ALIBOP	ATEDEI	AXERUQ
AXESEB	BAGCUJ	BAGDAQ	BAJTIQ	BASYAY	BAWGOV
BIYHEZ	BOPSAC	BUFWAB	CADJOI	CANVIW	CARDUU
CISWAF	CIXYOY	CLQUIC	CUNCOF01	DAYFER	DIFMOW
DIMPOG	DIXTOU	DOTDUM	DUBZEG	EBEZIU	EDEGIE
EDOCUX	EDUZUZ	EHEWOE	ELOQEE	EMUTUE	EPOJID
EQIVEI	ETEMOH	EVERAZ	FACNEG	FACROR	FAQZII
FEMZAA	FIQLIB	FOBMIT	FOBMOZ	FOWKIM	FOXVOF
FUFNOJ01	FUFNOJ02	FUWMOA	GAJSET	GAJSIX	GAYWAH
GEBJEF	GECDUP	GERBAJ	GESKIZ	GIPDAM	GIWMEF
GOLZEM	GOWXEY	GOZSUL	GUSXEX	GUZLIX	GUZMEU
HAYDUG	HAYDUG10	HCPYIN	HCTDPY	HIFWUO	HORSOY
HUMNOS	IJIHAL	IJITUR	IMAPIW	INADAE	INADOS
IYIYAQ	JALNOC	JEHYIF	JETVAI	JEYPAF	JUHKUU
JUHLIJ	KABBIA	KUPVAS	KUQTOG	KUTGEM	LADPUF
LEQFOF	LEQFUL	LEYPOU	LIQVIS	LIQVOY	LIQVUE
LIQWAL	LOBVIJ	LOFXUC	LOFYAJ	LULZAW	LUSZIL
MAZZET	MEDJUB	MEQNUS	METPOR	MOYJEQ	MOYJIU
NARLAT	NAZBOI	NIYGUZ	NOMJEE	NOQWEW	NOZKOF
NOZKOF01	NUKFIK	NUKFUW	NUPMUH	NUSKIX	OBUQIN
OCESIB	OGAXAW	OMUMER	OPIXIV	OQILAD	OWOZOQ
OWOZOQ01	PACMEN	PAQHEX	PATKEB	PELKUN	PELKUN01
PELLIC	PELLUO	PEYLUD	PEYMEL	PIJGOG	PIMYES
PIRDAX	PODXUB	PORPOD	PORWIC	PUHGAB	PUHHIK
PUKJUB	PUMHAI	PUVTAE	PUVTEI	QAPROP	QAWPAG
QEDCAF	QOWPAW	QUXDET	RAGQUO	RAGZUX	RAVVEP
REMDIW	RIDSAY	RIKFEZ	RODYEP	ROJFIF	ROJFIF01
SITPES	SODLEF	SOKKIP	SOLJIN	SUGSOF	SUJPEV
TERPAI	TIPQEQ	TIQKIP	TONVIE	TUBYUN	TUGBUU
TUNNIA	TUPGAO	UBOHEA	UCOGEZ	UQELOS	UQETEQ
URAXOB	URAYUJ	UREDEC	UVAZOH	VANCAR	VELCAR
VELCEV	VELCIZ	VELDAS	VETHUY	VICXIP	VIXWUX
VOJZUQ	VOQMEU	VURLUQ	WAKMEB	WANQAE	WANQEI
WEVKIU	WINQEQ	WIZVUX	WOGVUK	WOHZEA	WULSAA
WUPNUT	WUPPAB	WUVBEX	XAQVAP	XAYPUK	XECYOT
XEMZAR	XEPRIU	XICMEE	XIJNOV	XIXTAB	XOKGUA
XOLMUJ	XONQEY	XUGXUU	XUGZOR	XUMDAN	YAFLEW
YAMMOP	YATKOW	YATKOW01	YELMAG	YOSHOD10	YOYBUK
YOYREK	YUJMAU	YUPPUV	YUQROS	YUYTUJ	YUZGUV
YUZHIK	YUZSIX	ZAGQAD	ZAKQIO	ZAQVEU	ZAQWIZ
ZEVMOE	ZIDXIW	ZIGTEO	ZIGTIS	ZOTWAI	ZOTWUC
ZOVWUC	ZUBVEA				

m-chloroheterocycles with a XB contact

ADILUV	AJISEU	APOCUG	APODAN	ARAMUD	BAGCET
BAGCIX	BAGCUJ	BAGDAQ	BAPMAJ	BAPMUD	BAWGOV
BEKBUP	BIZFEW	BOFVAU	BUJHOG	BUKGOG	CIGLEK
COBFAC	COBFIK	COPREG	COXZOF	CUHJIZ	CUJYOW
CURLIM	DADFET	DIHGEH	DOQQEH	DOXKAE	DUCXAB
DUHQUT	DUHRAA	EBUZEI	ECACIU	EDEGIE	EZONOY
FARHOY	FEMVUQ	FEQHIU	FORHAX	FOSPIR F	UWGOW
GALHIN	GEKXEB	GOWXEY	GUFLEB	GUPICL10	HAKPIU
HIHGIO	HODJAM	IBICEC	IDUTEH	IMUROZ	INAFAG
INAFEK	IPOJOP	IQEWAF	IRUDOP	IRUFIL	IRUGAE
IRUGEI	JABLOO	JETVAI	KARDEN	KESSUZ	KETVEM
KOBFOX	KUPVAS	KURGOU01	KUXMIB	LAQNEX	LEBSAO
LEGQAS	LISQAI	LUSZIL	MABSUH	MCPHOT	MIFFUD
NARZUE	NECNOY	NIPDOH	NIXWIB	NUJYAV	ODEDOT
OGEZIJ	OGEZOP	OMAZUA	OPUPAT	PACLEO	PCLPYR03
PEDWOM	PEDWOM01	PEDWOM02	PEDWOM03	PEJLOF	PORWIC
PUHWEX	PUHWIB	PUVCER	PUVTAE	PUVTEI Q	AKBAH
QIVSIA	QOZXUA	QULJAKR	EMDIW	RUFXUM	SUGSOF
SUJJAL S	UJPEV T	EDPEA	TESHUX	TOHNEM	TURWAE
TURWAE01	UQETEQ	URAXER	UZUZAS	VACCEJ	VAGSOO
VAMFIB	VAMFOH	VESWAU	VEZWOO	VOFXIA	VORHAO
VUXNIO	WAQLEG	WICZIU	WIPDAD	WOHZEA	WUDHEK
WUPQEG	XACHIW	XIFMAB01	XUFMET	XUGXUU	XUVNAE
YAGCEP	YELMAG	YOSHOD10	YOXBIW	YOZZAQ	YOZZAQ01
YOZZAQ02	YUXPIT	YUYVOF	YUZHIK	ZESQIA	ZIDXOC

p-chloroheterocycles with a XB contact

AKATAI	AKEYAT	BAFHUO	BIWHIZ	DCMPHN10	DIFQOB
DIFQUH	DIFRES	DIFRIW	DUJQIJ	FOFZEI	FOFZUY
FUMJOP	GAKXAS	GUNCAW	GUZYOR	HCPYIN	HUVBIJ
JIRGOH	JIZVUL	KOGLIC	LISVOA	LULZAW	NEYJEH
OJAHAK	PACRAO	PANFOB	PCLPYR	PCLPYR02	PCLPYR03
PCLPYR04	PCLPYR05	PCLPYR06	PODXUB	PORWIC	QAHSOH
REMDIW	RIKDOH	RIKDUN	RIYXIH R	OWYOU	RUDHIK
UDEWAD	UQEMAG	VEWFAH	VEWFAH01	VURLUQ	WAFNOI
WEGVUA	WIBGOG	XADFOB	XAXFOU	XEQNOX	XOTBAM
YELDUR	ZAQNEM	ZUSLIK			

o-bromoheterocycles with a XB contact

ABOWUK	APUQOT	APUQOT01	ATILIY	ATILOE	AXONAE
BAFGEW	BAGCOD	BAVLOC	BIYNOP	CARRAR	CEKTET
CEKTOD	CEXWIO	CEXWOU	CEXWUA	CEXXAH	CUPLEH
DEYPAZ	DONRIK	DUFWIL	DUKFOH	EFUZAJ	EGIDAA
EGIDEE	EQABOP	ETADIO	EVAKIX	FARHOW	FEDVIU
FEGCIE	FILMAR	FIPGAN	FIQKEW	GUNSUG	GUZLOD
GUZLUJ	HIJCAG	HIMNOJ	IGOJEW	IGOJIA	IGOKAT

IGOKEX	IMUQOY	INADEI	INADIM	INADUY	INAFAG
INAFEK	JUHKII	JUHKOO	KEKZIK	KEMVIK	KETWIQ
KIWXUM	KOTMIP	LEGPAR	LOBVEF	LOCWEG	LOTFUV
LUMMIS	LUMSAP	MUHNUB	NAJWAA	OGIDUF	OJUKEL
OLOWOE	OQOJEM	OQOLEO	PIMZUI	QAMRUU	QEGSIH
QUNJAN	RAYTEQ	RIKDIB	RIKKUU	RIVPOE	ROSBUZ
SIQRUI	SIQSAP	SIZCEM	SIZDEN	SUKZUV	SULWEC
TEPXAQ	TUFXIC	UDEWIL	URUJID	VIXWIL	VORFEP
WACWII	WUTZET	WUTZUJ	XALMEF	XESQOB	XIGFAV
XIJNUB	XOBPIQ	YAGLEY	YELREN	YELSAK	YUYVEV
ZIMLIT	ZOTWEM				

m-bromoheterocycles with a XB contact

ACELEA	ADAQUS	AFOMUF	AJAPOS	AKICOP	ANEFOP
ANEFUV	AQAYUO	ARAMOX	AXADIN	AZUWUO	BAGCOD
BAYLIY	BIFTIV	BMIOXP	BOYHAZ	BUDHUE01	CARREV
CEBMED	CEWWIM	CIZJAY	COBDOM	DAMSAM	DIVMUT
DOPLUP	DUJQAD	DUKGEX	DUKVUD	DUPDUO	DUTKEK
EBERIN	EPUPUD	EROHID	ETUPUF	FEMWAX	FICXEW
FILQID	FOFFEO	FOFGAL	GAQFEL	GEVROR	GIBCUR
GUZLET	GUZLIX	GUZMAQ	GUZMEU	HIXZEV	HIXZIZ
HUGJOJ	IMUQOY	IMUQUE	IMURAL	IMURUF	INADAE
INADEI I	NADIM	INADOS	IPOCIB	IRUFAD	ISOHAB
JODTIH	KIZRUJ	KIZRUJ01	KONKOQ	KONKUW	KONTOZ
KOSKIP	KUBLOJ	KUHZEU	KUSPIY	KUSPOE	LADCIE
LADCOK	LADDAX	LADDEB	LEGPUL	LEJLER	LIBYAZ
LIQVIS	LIQVOY	LIQVUE	LIQWAL	LIQWIT	LIQWOZ
LISQOV	LISRUC	LUBBOZ	LUPDIL	MACKOS	MBNERG10
MELSII	MEPDIV	NAVJOL	NAVJOL01	NOLLAD	NUJWAR
NUPYAZ	OBEMOA	OBENAN	OCIXAC	OCIXEG	OJUKOV
OMEGIY	OPUNIZ	OSEJUT	PADDAC	POCJEY	PORNOB
PUJJUZ	PUKDIJ	PUVVEK	PUYYIT	QAFQOE	QEDKES
QOLSEQ	QULJEP	QUXDET	RAQJIE	RAQJUQ	RAWWAQ
RAWWEU	REFDIS	REMBUI	REMCET	RIKDEX	RIKDUN
RIKFAV	RIKKOO	RIKKOO01	RIKLAB	RISYUO	ROWVAD
SADNOB	SOTDEK	SULWAY	TADZUX	TEHNAX	TESLEK01
TIFLEZ	TIFLID	TIFLOJ	TIJJUS	TIPGEG	VELVUG
VIVREZ	VOTLEX	WACWII	WACWOO	WANWOX	WAPVIV
WAWFUY	WIBGIA	WIZVUX	XALGOJ	XOCQOX	XUTKED
YABDEN	YOCXUM	YODHIL	YODZUP	YOFLOX	YOFLUD
YOFMAK	YOFMEO	YULYOU	YULYUA	YUQQIL	YUYVUL
YUYVUL01	YUZSIX	YUZSUJ	ZAPWAQ	ZEFLAA	ZUPPEI
ZUPPIM					

p-bromoheterocycles with a XB contact

MABYEX	AFUMAS	AXOHUS	AXOHUS01	DOHXIK	FAHKIL
FOFYOR	FOFZAE	FOFZOS	GUZLOD	LISVIU	MICJUE
MUXZOW	MUYMUQ	MUYNAX	RIKCUM	RIKLAB	RISDUV

SIBTAZ	SIBTAZ01	TIMYEV	VEWDUZ	WIBGIA	WONKOA
XEBSIH	XEBTAA	ZAVFEK			

o-iodoheterocycles with a XB contact

ANUFAS	BAGCET	BAGDAQ	CAHZOC	CAHZUI	CAJBAS
COBDUU	COBFAC	DUJSAD	ELEWOI	GIBGEG	GIBGIK
IFUPAD	IMIVEI	IMIVIM	IMIVOS	IMIVUY	IMUQUE
IMURAL	IMUREP	IMURIT	IMUROZ	IMURUF	LUGPUA
MOSFUW	OPEYEO	QADVID	TAQSIQ	UDEWOR	WULSOO
YEBCOA	YELQUC	YOVDOD			

m-iodoheterocycles with a XB contact

ANUFEW	BACSUX	BUFWAB	CAZZAH	CIZKED	DINGEP
DINGIT	DINGOZ	EROQEJ	EVUXUQ	FEMWEB	IBIJUB
IMUQOY	IMURAL	IMUREP	IMURIT	IMUROZ	IMURUF
INADIM	INADOS	INADUY	INAFAG	ISOFEC	LIQVIS
LIQVOY	LUGPOU	LUGPUA	MAVVEM	MAVVIQ	NANREC
NECYOL	OBIRIC	OPUNOF	OPUPIB	OPUPUN	PACMIT
PACPAO	PACPAO01	PUYYAL	REMBUI	REWGIJ	SUJJAL
WARXEU	XAFPAX	XEPKIP	XIHFEZ	YUDJEN	ZUWZAV
ZUWZAV01					

p-iodoheterocycles with a XB contact

BAGCIX	BAGCOD	BAGCUJ	BAGDAQ	BAGDEU	DAYFER
DINGUF	DINHAM	DINHEQ	DINHIU	DINHOA	DINYIL
DINYOR	FOFZIM	JAQNOF	JAQNUL	LACWOD	MABVUK
QADVEZ	QADVID	QADWAW	QEDKIW	QUPNAT	TAQSAI
TAQSEM	UREJOS	UYOVIO	UYOVOU	VOTLEX	WIBGEW
XALHEA	YIFYOC				

o-chloroheterocycles without a XB contact

ABEROP	ABOJIL	ACALUN	ACAMAU	ACAMOI	ACANID
ACANOJ	ACANUP	ACAPEB	ACAPIF	ACIRIN	ADASEE
AFAYUD	AFINOU	AFOHOU	AFOPER	AFUHEQ	AHAJIE
AJIVOF	AJIVUL	AJIWAS	AJIWEW01	AKAGOK	AMELEM
AMPDAM	ANOWOR	APIFEL	APOTIL	ARELAL	ARUVOA02
ASOPAA	ATIBOW	AVAGEJ	AXESAX	AXESIF	AXONEI
AYONEI	AZIGUM	AZUNUF	AZUPIU	BACVIO B	AGDEU
BAGDIY	BAGJEA	BAVPOF	BAVPUL	BAVQAS	BENFIL
BENFOR	BEQSEV	BESGIS	BICSOY	BIZFEW	BIZKUS
BIZLAZ	BIZVIR	BOPFIX	BOXRIR	BOXROX	BUDZAC
BUFGEQ	BUFWEF	BUJHOG	CABVIM	CADJEY	CADJIC
CALHII	CANDEB	CANDEB01	CAPVIA	CAZRAY	CEJMAI
CEYHIA	CHPYRD	CHPYRD01	CHXIQL	CIQFUE	CITWEI
CIWMED	CIWMON	CIXYUE	COBXIC	COLBAI	CPYRIF
CUNCOF	CUNGUQ	CURLIM	DAKKUV	DATNAP	DAYFIV
DEBZUH	DESCIO	DEVSON	DEVYEJ	DEVYEJ01	DEXXAI

DEZXEO	DIFBEC	DIFJUA	DIFVAS	DIGWEW	DIGWIA
DIHFUV	DIHHAE	DIHHEI	DINHEQ	DINHIU	DINHOA
DIVCUI	DIXTUA	DOCMUE	DOJXEG	DOKRIH	DOKRON
DOKRUT	DOTSOU	DOXMOV	DOYLIP	DUCSIE	DUDFOA
DUKKUQ	DUMPUY	DUTTIV	DUTTIV10	DUVJEK	DUYHUC
DUZTEZ	EBITER	ECIKUW	ECILAD	EDEREL	EFEYAR
EFEYEV	EFINUF	EFUMIC	EFUMOI	EHIYEC	EJOGUI
EJOHAP	EKIJAK	ELEREV	ELESAS	EMAQOA	EMAQOA01
EMAYID	EMUTIS	EMUTOY	EPOJAV	EPOJEZ	EPOJUP
EPOKAW	EPOKEA	EQIROM	EQIVIK	ERIYEJ	EROCIX
EROZOB	EVACEL	EVELUN	EVUTUM	EYATII	EYILON
EZEMOM	EZEMUS	EZENAZ	EZENED	FACNAC	FAKVAS
FARHOY	FEQLUL	FIDCAY	FINKIX	FINKIX01	FIQKIA
FIQKUM	FIQLOH	FOBHOW	FOBHOW01	FOBMEP	FOKMUQ
FOQFEY	FOSPIR	FOWVOD	FOXVUL	FOXWAS	FOXWEW
FUBLIY	FUBYEJ	FUFNOJ	FUGWAG	GADVAL	GAGGON
GAGGUT	GAQTUP	GATLOE	GAVJIX	GECDUP01	GECFAX
GEHYEY	GETVEH	GEVDOB	GEVFUJ	GEZNII	GICGIL
GIGXAV	GIPZAH	GIRVAF	GITMUS	GIVFAV	GIXQAI
GOGKIX	GOGYEJ	GOKNAX	GOLZAI	GOPPOT	GOQFIE
GORNEJ	GOYQUI	GOZSOF	GOZTAS	GUGZUE	GUGZUE01
GUHSOS	GUKYUJ	GUKZAQ	GUKZEU	GUPICL10	GUSXOH
GUSXUN	GUZLET	GUZMAQ	HAJCOL	HAJDIG	HAJDUS
HAJFUU	HANBAA	HANFOS	HANFOS01	HANFOS02	HANFOS04
HEQWIK	HESNUP	HEWZUH	HEXMEF	HEXNIK	HEZBOF
HIFXAV	HIJCAG	HIJCEK	HIPWEI	HIRLAW	HIVGAX
HOBXOM	HODJAM	HOGVUW	HOLHAS	HONLUR	HORSUE
HOTVOC	HOTZUM	HUFXAH	HUGHOH	HUGHUN	HUYSUR
ICUGOE	IDOQOI	IFAXAP	IFUWAI	IGEYUO	IGIBAE
IGODAL	IGOKIB	IHIZUW	IHOHOF	IHOHOF01	IHOYEM
IHOYEM01	IJEGAF	IJIGOY	IJIGOY01	IJIGUF	IJIHAL01
IJUTUD	ILUDOL	ILUDUR	ILUFAZ	IMAPOC	IMIBUB
IMICAI	IPCPYA	IRABOS	IRABOS01	ISATIH	ISICET
ISOFIG	ITUTIC	IVIQUZ	IVOBEC	IWIVUG	IXOYIE
IYIXUJ	IZITUH	IZIVET	IZIVUJ	JALNIW	JAYVAH
JEBNOU	JECVIX	JEDRUG	JEDSAN	JEDSER	JEDSIV
JEDSOB	JEDSUH	JEDTAO	JEDTES	JEDTIW	JETTUA
JEVVEM	JIPXUC	JIWNEI	JUSCEI	JUSCIM	JUSCOS
JUSCUY	JUSDAF	JUSDEJ	KARQEC	KARQIG	KATHEV
KEBDIF	KEGGUA	KENCAJ	KEQLOI	KEQLOI01	KEQLOI02
KEQVAE	KEXYOC	KEXYOC01	KEXYOC02	KEXYUI	KEXZAP
KEYNOS	KEZPUCKIKJIY	KIKJIY01	KIKLOG	KOCJUI	KOGYEN
KOYZEG	KUGBUK	KUGCEV	KULQOY	KUSMER	KUSMER01
KUSQOF	KUVBEJ	KUVCOU	KUVFEN	KUVLET K	UZLEX
LACYIZ	LACYOF	LACYUL	LADQAM	LAMDOW	LAQTUU
LAVZAM	LAXDOE	LEHKUF	LEJPOG	LEMXOQ	LENYAG
LILJOH	LILJUN	LIQWEP	LIQWIT	LIQWOZ	LIWJUX
LIXCEA	LOHRUW	LOHSAD	LOTYIE	LUFWOB	LULZIE

LUNYAW	LUNYAW01	LUTJAO	LUZTAE L	UZTEI	MAFSOD
MAFSUJ	MAFTAQ	MAFTEU	MAJTOI	MARGOB	MASGOF
MCPHOT	MEGYAZ	MEHDAF	MENMUO	MEPWUZ	MEPWUZ01
MEQPAA	METPOR01	MIGYEH	MINRAC	MINRAC01	MIZKUD
MOTDIJ	MOVMUG	MUDVIQ	MUKQAK	MUNSAR	MUVTED
MUVWAC	NADSAM	NAGYIF	NAGYIF01	NAHHIN	NAJVUT
NASGUL	NASXEL	NELZAI	NEPNIH	NEQPIJ	NERXOY
NEVKEF	NIDZIK	NIGKEW	NIJPUT	NIJQAA	NIMXOY
NINSAG	NIPQIO	NIPZUI	NIYHAG	NOCTEG	NOCTEG01
NOHDOF	NONBIE	NOQXOH	NORNIS	NOZLAS	NOZLEW
NOZLIA	NOZLOG	NUCYUH	NUHJIK	NUHJOQ	NUHKAD
NUHKIL	NUNJIQ	NUPLAM	NUQBUZ	NUQLER	NURFAI
NUSKAP	NUSKET	NUSKOD	OBIVIH	OBIVIH01	OCAGEG
OCEKEN	OCELEO	OCIQUP	OCIRAW	ODEKAJ	OGEZIJ
OGEZOP	OGINOK	OJAHEO	OMOHOP	OMOTOA	OMOVAO
OPIXER	OPIXOB	OTEDAT	OTEDEX	OTEDIB O	TEDOH
OTEDUN	OTEFAV	OWAMEG	OWECUP	OWEDOK	OWEDUQ
OWEFAY	OYUTIN	OZAPAI	PAGDAF	PALWEH	PAQHUN
PATTAI	PAVVIT	PAXNUZ	PAYBID	PAYBUP	PCLPYR
PCLPYR02	PCLPYR03	PCLPYR04	PCLPYR05	PCLPYR06	PEJLOF
PELLAU	PELLEY	PELLOI	PELMAV	PELMEZ	PEPFAU
PEVBIE	PIDJIX	PIDZIL	PIDZOR	PIKKOM	PILFAS
PIMXOB	PINVEN	POFCIX	POFTIQ	PONRIT	PONRIT01
PONXEW	POVJIT	PUHFII	PUHFOO	PUHFUU	PUHGEF
PUHGIJ	PUHJOS	PUJSAP	PUMGUB	PUVRAA	PUYSAG
PYOCHP	QAJHEQ	QAMNAU	QAXKUY	QEKNUQ	QIBZOQ
QIMREM	QINXIX	QINYAQ	QIRQEP	QIVSIA	QIYTAW
QOBKOI	QOBKOI01	QOFZAO	QOLLUZ	QOMQIT	QOMSOA
QOWNAS	QOWNUO	QOWPEA	QUDGII	QULQOE	QUNSOK
QUYGIB	QUYHEY	RABXEB	RAGCAG	RAGPUN	RAGQAU
RAGQEY	RAGREZ	RAKTEC	RAKTOM	RAPPOQ	RAXMAG
REHCUD	RELPIH	REWCEE	REXGIN	RIHYAK	RIKFAV
RIYXIH	ROLQEP	ROMNUD	ROMNUD01	RONGAF	ROWTOP
ROWTUV	RUJNUG	RURLUN	RUWLAZ	RUWLED	RUZZIX
SAGPOH	SARQIK	SAVBAT	SAVBAT01	SAWNUB	SEDDIQ
SIMQIP	SOQNUH	SOVYOU	SULSEY	SUXKAA	TAGVUV
TAGWAC	TAGYAE	TALVEK	TANYUF	TAQYOA	TAWJEG
TAWJIK	TCHYPY	TEJNOM	TEMFEY	TENHEB	TENHIF
TESHUX	TIGVOV	TINHUW	TINHUW01	TIWDUZ	TIWFEL
TIWFIP	TIWFOV	TIWGAI	TIWGOW	TOBPAC	TOLKEL
TOZPEG	TUBFAZ	TUBFED	TUCGUW	TUJMAO	TUJMES
TUJMIW	TUJMOC	TUJMUI	UCIVAD	UDOMEI	UDOMEI01
UJUNAQ	UKIXAQ	UMOCEE	UMULOD	UMUPIC	UNADIY
UNESAI	UNESAI01	UPICUT	UPICUT01	UQAPEI	URAZAQ
URAZEU	USOFEO	USOTUU	USOVAC	UTOLEV	UTOLEV01
VABJAL	VABLAN	VACDIP	VEFXAH	VELCOF	VELCUL
VIDCUH	VIKJAB	VIPQUH	VISJOZ	VISJOZ01	VOBBUJ
VOJYEA	VOJYIE	VOMDOT	VOQTIF	VOSFOA	VUHQAS

VUHQEW	VUHQEW01	VUNVIL	VUPFUJ	WACVAZ	WACZOR
WATKUX	WEDFIW	WIHJOP	WINXOG	WIZWEI	WOJBAA
WOJDEG	WOLHUB	WOXHUO	WOXJAW	WUDKEO	WUFBEG
WUFNIW	WULSAA01	WULSII	WULSII01	WULTAB	WULTAB01
WULXIN	WULXOT	WUMJEV	WUMJIZ	WUPFOD	WUPQOO
WUPQUU	WUVBAT	XAQLUY	XAQMAF	XAQMEJ	XAQMIN
XAQMOT	XAQMUZ	XAYFIP	XEBZEL	XECNEA	XEPHIL
XEVKOY	XEYQIC	XIBZIS	XIRDAE	XISSEY	XIVCIO
XIVDEN	XOMMUI	XONPUN	XONQAU	XONQAU01	XONQIC
XONQOI	XOSWIL	XOYVUD	XOZJED	XOZJIH X	OZXAM
XUBFEH	XUFNIX	YABJET	YAFLEW01	YAGMID	YAGMUP
YAGNEA	YAJCIX	YAMMIJ	YAMROU	YATLAH	YATLEL
YAVREU	YAWHOV	YEBHUL	YEDTIL	YEFDAR	YEHSUA
YEHTEL	YEKSEN	YELSEO	YEXGIS	YICNEE	YICNEE01
YIQWIF	YOBNEL	YOCGAB	YOHTEW	YOHTIA	YOSHOD
YOYQUZ	YUBKUD	YUCSOF	YUDFEJ	YUDKIT	YUHCAI
YUQRUY	YUQSAF	YUQTAG	YUQVIQ	YUYTUJ01	YUZGOP
YUZVOG	YUZWAT	YUZWIB	YUZWUN	YUZXUO	ZADVUZ
ZADWAG	ZADWEK	ZANROX	ZEVMUK	ZEVNAR	ZIGTEO01
ZIGTOY	ZIGVEQ	ZIJNEN	ZIRHUD	ZIRJAL	ZIRJEP
ZOKFAJ	ZOVWIQ	ZOVWOW	ZUSLIK	ZUTMAE	ZUTMIM
ZUWYEX					

m-chloroheterocycles without a XB contact

ACAKAT	AKAWOZ	ALEGEF	AMCLPY	AMCLPY11	AMCLPY12
ARANEO	ARANOY	AROBIV	AYEWOR	AYONEI	BAGDEU
BICROW	BICSAJ	BIFTER	BIKGEK	BIKGEK01	BIYJOL
BUBCEI	BUKGIA	BUKGUM	BUKHAT	BUKHEX	BUKHIB
BUZWAU	CANVIW	CFNPYR	CIJWOI	CISQUS	CIZJIG
CMXSAP	COBFEG	COMKAR	COPCAN	COPCER	COPQII
COXZEV	COXZEV01	COXZIZ	CPYPTZ	CPYRIF	CUHNEY
CUHNEY10	CUHNEY20	CUHNEY22	CUHNEY23	CUHNEZ	DADDUH
DADFAP	DEXXAI	DIHFUV	DIHGIL	DIXTUA	DOKWUW
DOQQEH02	DUCXAB01	DUJROQ	DUTSET	EBEQOS	EBEQUY
EBERAF	EBESAG	EBUZIM	EDELII	EGUVOR	EPOKOM
EVASIF	EVEWEJ	EXUVAV	EZAKAS	FAFGUP	FAFHUQ
FAFJAY	FAQREW	FEWHUM	FIDXOI	FIDXOI01	FUGWOW
FUQLIN	FUWGIQ	GASVUU	GAYMEB	GETVEH	GEWXOY
GITKUQ	GITMUS	GOHJAR	GOYLIS	HABMOM	HAJDIH
HALSES	HCPYIN	HCTDPY	HIFWUO	HISCES	HOFPUO
HUFWOU	HUHXUE	HUJFEY	HUKFAX	HUKFEB	IBUSEG
IDOQOI	IGAPOW	IHOYAI	IMURIT	IPCPYA	IQIBES
IQUROD	IQURUJ	ITUZII	IWEYOY	JABLAA	JABLEE
JABLII	JABMAB	JASGIU	JELZUV	JETTUA	JOCNIA
JOKLON	JOWWUP	JUGCOF	JUKFIH	JUVGAL	KARDEN01
KECHOQ	KENGER	KEQYUC	KESTAG	KESTAG01	KEZTIT
KIFVOM	KOFFOB	KOSWOH	KOYZEG	KURGOU	KUVHIV
KUZTOP	LARQIH	LEBSES	LEBSIW	LEDZEB	LEGPIZ

LEQWUZ	LITNUX	LOFWEK	LOHXAI	LOJBIW	LUCQEI
LUCQIM	LUTJAO	LUWNAV	LUWNAV01	MEGYAZ	MENMUO
MENSIK	MENSOQ	MENSUW	MEPXIO	MIWNAK	MOGZEP
MOJCUK	MOJDEV	MOMBUN	MOZMUK	MURRAV	NARBEQ
NARZUE01	NEBZAX	NEBZAX01	NECNUE	NECPAM	NEPNIH
NEQPIJ	NIXDUT	NIXDUT01	NIXDUT02	NIXDUT03	NIXDUT04
NIZKEP	OBUZOC	OJUKUB	OJUKUB01	OMAVIJ	OMUMER
OPEYEO	OWECID	PACMAL	PACMUF	PACNOA	PCLPYR
PCLPYR02	PCLPYR04	PCLPYR05	PCLPYR06	PCPOTZ	PEGXIK
PEWWAS	PIHCAM	PODXUB	POMYAS	PUGYAS	PUHWOH
PUJNIS	PUVLAW	QAFQOE	QAHKUI	QAMYUA	QAWPAG
QEDCAF	QETWUH	QEVHOR	QEVHOR01	QICTUR	QOFZAO
QUKKOY	RAVVEP	RAWZAS	ROWTOP	ROWTUV	SEMZIU
SIMQOV	SIXTOL	SOVWOS	SOVWUY	SOVXOT	SOVXUZ
SUJPIZ	SUSYUC	TCHYPY	TIHNUU	TOKKEM	TONVIE
TOSVUS	TOTBIP	UCUTUH	UCUVET	UCUVET01	UMUPIC
UNAHAU	UNAWEN	UNAWIR	UQAPOS	UWEDIL	UXIKAP
VAGSEE	VAGSII	VAGSUU	VAGVAD	VAMGEY	VAMGIC
VAMLUT	VAMMAA	VAWRUI	VEKGUQ	VEMQOV	VENKUV
VIDLIG	VIDLOM	WAVSIW	WECGIW	WECGIW01	WECGIW02
WECGUI	WECHAP	WIMCON	WUFYUT	WUFYUT01	WUKRAY
WULXEJ	WUPQAC	XAJHEY	XAJHIC	XANFAU	XEBPAY
XEFMIH	XIFLEE01	XIFLUU01	XIFMEF01	XOYVUD	YABJET
YAGXUB	YIFLUV	YIQWIF Y	OMGOZ	YOMWIJ	YOSHOD
YULGOD	YUZGOP	YUZGUV	ZIHDEC	ZOVKAZ	ZOVKAZ01
ZUSLIK					

p-chloroheterocycles without a XB contact

ABOVUK	AJETAL	AJETAL01	AKAWOZ	AKORIDA	NOWUX
BAWMIV	BIKFEJ	BIWGOE	BOPSEG	BOXVIW	BUFTEC
CIVCOC	CIYGEZ	CTMPMQ	CUHDOB	CUMTUB	DAFGOJ
DAFGUP	DATRUN	DIFRAO	DOMPOM	DUQMUZ	EFINUF
EREQOH	EREQOH01	EREQOH02	ESIFOD	ESIVEK	FAMPIV
FARMIW	FEJXID	FEQLUL	FEQMAS	FEQMUM	GAMHAG
GIXMIM	GOPPOT	GUDPAX	GUDPEB	GUDPOL	GUDPUR
GUFLEB	GUNCEA	GUNDOL	GUNFAZ	GUZLIZ	HABGIA
HEGPOA	HEXMIJ	HEXMOP	HUYSUR	IDEXOG	IKAQIV
IMAPUH	IPUSAP	IYIYOE	IZIVET	JIHFEL	JIRHAU
JUKBAU	JUSROG	KEKWOP	KIPCIX	KUPVAS	LISQIP
LOLVIR	LUFVOA	LUFVUG	LUFWAN	MURNOC	NAFGOU
NEYJIL	OGUCOL	PANFUH	PEDTIE	PEGXUW	PIDHAO
PUFTIW	QARGOF	QARGUL	QOBKOI	QOBKOI01	QOQXUP
QUYGIB	RAHROJ	RAZLIQ	RAZLIQ01	REBTID	REBTOJ
REMWUD	RIHMAW	ROGBAS	ROMNUD	ROMNUD01	ROWYUA
ROXFIW	RUNCAH	SALQON	SASFIE	SEVWEX	SOVXEJ
SOVYEK	TARHUS	TAWFOP	TIBXEI	TUGRUK	TUGSAR
TUNNIA	UDEWIL	UGEGIW	UGONOT	UMADER	UNAWEN
UTUZUF	UXEYUS	UXEZAZ	UXEZED	VEWFAH02	VUSVOX

WAFNIC	WAMSOT	WANNIK	WANNIK01	WANNOQ	WANPAE
WEDGOD	WEJFEX	WEJFEX01	WIQROF	XADGAO	XAJPOO
XEHNOO	XOFBUS	YAGMOJ	YAHDES	YIBYIR	YUCVAW
YUYTER	ZEPXAV	ZULCOY	ZZZAAC	ZZZAAC01	

o-bromoheterocycles without a XB contact

AFUKUK	AFULAR	AFULUL	AGUGOY	AJEWUI	ALOPEY
ALOPEY01	APAPAK	ATILAQ	ATILUK	ATIMAR	AYUKUB
BAJTEM	BAJTEM01	BAJTEM02	BEGJIJ	BEVCOX	BOBDON
BOMYOS	BRHXPY	BUVQEO	BUVQIS	CALVAO	CARREV
CATWUQ	CATXAX	CEKTIX	CEXWEK	CITTEH	CIWDUK
CIWMIH	CIWMUT	CIXYUE	CUPLIL	CUVDAA	DAQHAG
DEFGUQ	DIQZUA	DOHDEK	DOLWOS	DOLWUY	DONREG
DOTJUS	DUPKEF	EHITIA	EMAXEY	EMAXIC	EMAXOI
EMAXUO	EMAYAV	EQUVIW	EXAKAR02	EYIWUE	EZIFEA
FEGCAW	FILMIZ	FUTYEB	GESXAE	GIWBEW	GOZCEF
GUXTEA	HEMNUL	HIFTAR	HIGDIM	HOBBOS	HOBCEJ
HOBTOI	HODKUH	HOLSEH	HUBQUT	HUHBES	HUPXUM
IGOJOG	IGOJUM	IKELUH	JADQAK	JAGKAG	JAGKEK
JAJWOJ	JEDHEI	JIQKOM	KAGGUV	KAQXOR	KOVGAF
LAQWIM	LOBLEV	LOTFUV01	LUGPOU	LUMFUX	LUMGOR
LUMGOS	LUMGUX	LUMGUY	LUMYOH	MEHLOB	MEHLOB01
MOCSUT	MUSTEA	NAFGOU	NAPRUS	NIWJIO	NIZFOS
NIZFUY	NOSLOZ	OBOCIS	OBOCOY	OCATUJ	OHEKET
OJUKEL01	OLOGUR	OLOVET	OLOVET01	OLOVET02	OLOWAQ
OMIZAO	OQIXUK	OQIYAR	OQOJOW	OQOKUD	OQOLAK
OTANUT	PABCIG	PABPER	PAYBOJ	PEDWUR	PIMRIP
PIMZES	PURZEJ	QECDOU	QIYVEC	QQQGGY	QUBZUL
QUZPEH	RIKCEW	RIKDAT	RIKDEX	RISROB	SOQRUO
SUBXUL	SUKZOP	SUNSUQ	SUNTIF	SUYRIN	TAQSOW
TILPOT	TINVIW	TOKHOT	TOTWII	TUGNUG	UCURIV
UDEWEH	UDIZEO	UDIZEO01	UGAYIK	UHUFIM	UHUFUY
VOJNEN	VOMREU	VOMROE	VOQYUW	VOVZEO	VUGRAT
VUNRUU	VUVTEO	WAJCAN	WELYOE	WEPSEQ	WIWWEH
WOXHOI	WUTYUI	WUTZAP	WUXFAW	XALMEF01	XANYAO
XAZZEF	XEBSON	XEBTII	XECDUH	XUTKAZ	YEHKUT
YIFFEZ	YIKQUG	YUYVEV01	ZIFSIQ	ZIMLEP	ZOBPAK
ZOWWIR					

m-bromoheterocycles without a XB contact

ABOWUK	ACELIE	AFULUL	AFUMAS	AJIMAI	ALACOG
ANIJEO	ARANAK	ARANIS	ARAYIE	AXABIL	AXAMOC
AXAMOC01	BAPNAK	BECZIU	BNMIER	BOBDON	BPYFSB
BUDHUE	BUDHUE02	BUVFAA	CADGAR	CAJXAN	CARRAR
CAYHAM	CEQREY	CEWWEI	CIGGAD	CIPYOQ	COBDOM01
COBDUU	COLVOP	COPREF	CORGEX	CUCXAZ	CUYMER
CUYQEV	CUYVIE	CUYXEC	DAKROZ	DAWTUT	DECGOK
DEZPAZ	DEZXIR	DOGQEX	DOGYUT	DOGYUT01	DORWAL

DUSFAZ	DUSKUY	EBESEK	EBESOU	EBETEL	EBEVAJ
ECAHOI	EGEXOF	EKIKIS	ELOCIT	ENELEP	EVAYUW
EWIYIS	FEGCAW	FEGCIE	FIFCAA	FIFCOO	FINHOC
FIQQII	FIQQII01	FOFFAK	FOFFUE	FOKKOH	GAHNAF
GAHNEJ	GAXCAM	GEJKIS	GENGAK	GEVROR01	GEWVIQ
GICVUJ	GICVUJ01	GICVUJ02	GICVUJ03	GOLSAC	GOYLUE
GUPFUV	GUZLOD	HAZNAZ	HAZNUT	HOBLAL	HOQPOV
HOTQAJ	HUZCOU	IBURIJ	IHELOY	IYARUW	JOLQAE
JUVGEP	KADHIK	KAZHEB	KEHRUM	KEKWIJ	KEPFOB
KIKLUM	KOMQUZ	KONLAD	KOSWUN	KUBLUP	KUSPEU
LADCAW	LADCEA	LADCUQ	LADDIF	LEGPEV	LEJLAN
LEJLIV	LEQWOT	LEQYIR	LIBYED	LIBYON	LIBYUT
LIQWEP	LOBXAC	LULZEA	LUQLOZ	LUQLUF	MADMUD
MADNAK	MBNERG	MEWSOY	MEWSOY01	MEWSOY02	MUPVOK
MUXHOE	MUYHUM	NAVJUR	NAVJUR01	NAZWUJ	NAZXAQ
NICNUK	NICPAS	NICPEW	NIQRIP	NIZLAM	NOLKUW
NULWOI	NUPXUS	OBEMEQ	OBEMIU	OBEMUG	OBENER
OBENIV	OCATUJ	OCIWIJ	OCIWUV	OCIXIK	OFADOP
OFIFUH	OGEDOU	OHEZEH	OJUKIP	OJUKIP01	OJUKOV01
OMEGAQ	OMEVUA	OQOJUC	OSEKAA	PACMEP	PACNAM
PACNUG	PESSUC	PESSUC01	PIXQEV	QAJXII	QAJXII01
QAJXOO	QECDIO	QECLIT	QQQFDD	QUCCUN	RAQJEA
RAQJOK	RAQKEB	RAQKIF	RAVXIY	RAVXOE	RAWWOE
REMBOC	REMCAP	REMCIX	REYQUK	RIFLOK	RIKCEW
RIKCUM	RIKDOH	RIRCEB	RISDEF	RISFAD	ROSGOY
RUZPUY	SAHJUK	SAHJUK01	SAHKEV	SAHKUJ	SAHKUJ01
SAWTIV	SEKVEL	TAJBOV	TAJBOV01	TAYJOT	TESLEK
TESPAK	TETPEO	TETPOY	TIFLUP	TIFLUP01	TOKFUV
TUBFAZ	TUGYOM	TUHCAB	UCOXES	UCURER	UKUZUX
ULEWAK	ULEWAK01	UNULOF	UNUWIK	URAQOU	VEJSAG
VIPYUO	VOJHEJ	VONGEM	VONHAJ	VOQYUW	VUHQAS
VUHQEW	VUHQEW01	VUPVAF	VUXSUD	WAGPEC	WANXOY
WANZUJ	WIBGEW	WIBGOG	WIHHON	WIHHUT	WIRLUH
WOTRIJ	WUMJIZ	XAXFOU	XIFMOP01	XITLUK	XITLUK01
XOBJOQ	XOCQIR	XUGYAC	XUGZOR	XUMRUV	YARQOZ
YEHTAH	YIBXAJ	YIKXOH	YODZUP01	YULYIO	YUSLEF
YUZSOD	YUZVOG	YUZVUM	YUZWAT	YUZWEX	YUZWIB
YUZWOH	YUZWUN	YUZXIC	ZIYHEX	ZOCMAI	

p-bromoheterocycles without a XB contact

BERYOP	BIZYOZ	CAWMAQ	DOHXIK01	DUTVAQ	ECUXUV
JETTUA	JETVAI	LISROW	LUBJOK	MAVVIQ	MAVVIQ01
MUXZIQ	REHROO	RIKCOG	RISDIJ	SAWTUG	SAWTUG01
SIBSOM	UDEWOR	UDEWUX	UDEXAE	UDIZEO	UDIZEO01

o-iodoheterocycles without a XB contact

CAVRIB	CAVROH	FAKTUK	ISODUQ	JETTUA	QADVEZ
QOLSEQ	QQQGHA	TAQSAI	TIPNOX	UYOVIO	UYOVOU
WEPSIU	WULSEE	WULSEE01	WULSOO01	XUGLEQ	

m-iodoheterocycles without a XB contact

ASOQIJ	EQOLII	GIJTOK	GOYMAL	INAFEK	ISOFIG
JOCTEB	JOCTEB01	JOCTEB02	KEMVIK	MAVVEM01	MAVVIQ01
NOQCAY	OBIREY	PACLOY	PACNEQ	PAQMOM	PUGPOX
QADVUP	RIRCAX	RIRCAX01	RUHWID	TIPQEQ	VEGTIM
XEVREY					

p-iodoheterocycles without a XB contact

BIZPEG	DINHUG	ICITAS	MAVVEM	MAVVEM01	UREJEI
VOMDOT					

II. Protonated halogenoheterocycles

Protonated *o*-chloroheterocycles with a XB contact

AFUDOW	BONZAG	BUBWIF	CALHEE	CATXOK	CIHCIG
CLPYSB	DIBVUG	DOCNEP	EMIFUE	FISBEO	GICFOP
IKUCAU	LAVNUU	LAVNUU01	MADPAK	MADPAK01	NOKVIS
NOKVIS01	OFELUJ	PEGVAB	TAWJIK	TEJCOD	UBEKAP
XAHYIS	ZIFHII	ZOLXAC			

Protonated *m*-chloroheterocycles with a XB contact

ALAHUQ	BEKCAW	BIJRUK	BUVSER	BUVSIV C	EFJIJ
COPRIK	DEDPOU	DEDPUA	DEDQAH	DENFIO	DIBWER
EMIGIT	FISBOY	FISCAL	FUGWIO	HOTJIK	IDENUB
IKUCEY	JIPHAS	JUGWUF	KUZJIZ	NOGDOC	NULRES
NUWGAP	OHIWEI	PEBYIG	PEBYOM	PELGAP	PELGAP01
POMKUZ	SACTAS	TIZZUA	TODFAV	TUCQEQ	TUQKEW
UHUHEM	ULASAC	UXENUI02	VEPKOR	VEPKOR01	VEPKOR02
VEPKOR03	VEPKOR04	VEPKOR05	VERBAY	VOQMUJ	XOMVAX
ZESQIA					

Protonated *p*-chloroheterocycles with a XB contact

AGEYAN	AGOLIU	AROVOV	CAVKER	CAVKUH	CIHCAY
DIBWIV	DIBWOB	DIBWOB01	DIBWOB02	DIBWOB03	DIBWOB04
DIQZEI	FEJXID	FETYIO	FISBUE	FISLEY	JAQNIZ
JETDER	JETDER01	JETGEU	KIWGED	PEGSAW	PEGSEA
POBFER	SAZYIC	SAZZID	SAZZID01	SAZZID02	SAZZID03
SAZZID04	SAZZID05	SAZZID06	SAZZID07	SAZZID08	SAZZID09
SAZZID10	SAZZID11	SAZZID12	SAZZID13	SAZZID14	SAZZID15
SAZZID16	SAZZID17	SAZZID18	SEHHET	SEHHIX	SEHHIX01
SEHHIX02	SEHHIX03	SEHHIX04	SEHHIX05	SEHHIX06	SEHHIX07

SEHHIX08	SEHHIX09	SEHHIX10	SEHHIX11	SEHHIX12	SEHHIX13
SEHHIX14	SEHHIX15	SEHHIX16	SEHHIX17	SEHHIX18	SIWVOL
UXEPAQ02	UXEPIY02	UXESOH02	VADBAF	VADBEJ	XEHNUU

Protonated *o*-bromoheterocycles with a XB contact

AGEVOY	AGEVUE	AGEWAL	AHUVUU	AHUWAB	BEGJIJ
BIZCIX	CAKNAF	CIGZIC	CIGZOI	CITTEH	DEMQOE
DUKSUY	DUKTAF	DUKTEJ	EMIGEP	EQUVOC	FEBGEZ
FEBGID	HAGYOE	HILZAG	HIMBEN	HIMBIR	HIMNOJ
IJOHUK	IJOJAS	MOPHOP	MOPHUV	MOPJAD	MUGWOC
NAJWAA	RACLIR	SIVJEP	TEPXAQ	TEPXEU	TIZZOU
TOBCIX	UXERAS02	VINQOA	VINQUG	VOQMOD	VOQMOD01
VOQMOD02	WOCROX	WUTJOK	ZUMNUT		

Protonated *m*-bromoheterocycles with a XB contact

AFULIZ	AGEVOY	AGEVUE	AGEWAL	AHUWEF	AHUWEF01
AHUWEF02	AHUWEF03	AHUWIJ	AHUWIJ01	AHUWIJ02	ALAJAY
BEXPIF	BRPYCH	CACSOP	CEFFEB	CIHBAX	CIHBAX01
CIHBAX02	CIHBEB	DICJUV	DOQQOR	DUVCIH	EMAYIB
EMAYOH	FORKUS	FUHRAB	HIBLIQ	HILZEK	HILZIO
HILZOU	IDUGIZ	JUGWOZ	JUHLEF	KECJEK	LABQIR
LADBUP	LAKWOL01	LEJKUG	LICBEF	LIFLOD	MAJTOH
MISMAC	MOPHOP	MOPHUV	MOPJAD	MOPJEH	MOPJIL
MOTBUT	NAJWOO	NERWEM	NULQIV	NULRAO	NULRIW
NULRUI	NULSET	OHIWIM	OHIWOS	OHUQUG	PEBYAY
PEBYEC	PEGTON	PUKJIP	QOVLEV	QUBLEF	QUBLIJ
QUSDAK	RISDAB	RUZXOA	SUYXIU	SUYYAN	SUZBEV
SUZZIX	TUCPEP	TUCPIT	TUCPOZ	TUCQAM	TUCWEW
UJODUS	UXEREW02	UXEROG02	UXERUM02	VERBEC	VERBIG
VINQOA	VINQUG	VOQNAQ	VOQNIY	VOQNIY01	WAGDIT
WAGDIT01	WIGWUG	XACYIM	XEYXAB	XILVAQ	YAGXIO
YEFWIQ	YIKQIU	YOKSEY			

Protonated *p*-bromoheterocycles with a XB contact

AHUWOP	AHUWUV	CAVKIV	CAVLAO	CIHBOL	CIHBUR
DIQZIM	FETYEK	GALKOT	GALKUZ	HEBDAW	HILZUA
HIMBAJ	JAQNEV	JETFUJ	LABQOX	NUFGAX	OMOLAG
OMOMEL	OMOMEL01	PEGSIE	PUQSAW	RISDAB	SAZYOI
SAZYUO	SAZZAV	SAZZEZ	SIVJIT	TEHVIO	TEHVIO01
TEHWEL	UXENOC02	UXESAT02	YOKLAO		

Protonated *o*-iodoheterocycles with a XB contact

BEXPOL	FOBSIA	FOBSOG	HIBLAI	JULREQ	JULRIU
JULROA	JULRUG	SIVHAJ	VOCTIS	VOCTOY	

Protonated *m*-iodoheterocycles with a XB contact

ALAJEC	CIYVUE	CIYWAL	FOBSUM	FOBTAT	IGAMIM
IGAMIM01	JAQNAR	JULSAN	JULSER	JULSIV	JULSOB
NAJWUU	NUFTOA	NULQOB	PAXYET	QIQGAA	QIQGEE
TIZZEK	TUCPUF	TUCQIU	TUCRAN	VEGSIL	VEGSOR
VEGSUX	WUWNAE	WUWNEI	YAGXUA	YAGXUA01	URACEY

Protonated *p*-iodoheterocycles with a XB contact

AGOLAM	AGOLEQ	CAVKOB	CAVLES	DIQZOS01	FETYAG
FOBTEX	FOBTIB	JULSUH	JULTAO	NAJXAB	TEHVOU
TEHVOU01	TEHWIP	INOPAF	OMOLEK	OMOMAH	OMOMAH01

Protonated *o*-chloroheterocycles without a XB contact

ACALUN	ACAPAX	ACEHEY	BONZEK	CALHII	CATJEO
CIGZUO	DEMQUK	DIBVUG01	DIBWAN	DUDFUG	DUDGER
LOTYOK	MOGBEQ	MOGBIU	OGEMEV	OGEMEV01	PIFJAQ
QOVLIZ	QOVLIZ01	QQQGRM	ROLRAM	ROLRAM01	SAQSAH
TIZZIO	UFIPUU	UFIQAB	UFIQEF	VOQMIX	YIKCED

Protonated *m*-chloroheterocycles without a XB contact

ACUBIL	AFAYIP	ALAHUQ01	AREJUF	CASBEE	CEFJOP
CIHBIF	CIKWUP	DEGMUY	DOQQIL	DOQQIL02	DOSNEF
DUPFUQ	DUSYIA	EGUVEH	ELULAB	EMIGAL	FOVBUO
FUTRER	GOJROO	HIBLEM	KOJFEX	KOXWEB	KUSVEA
MOGYUF	MOGZOA	MOHCAQ	MOYKES	MOYKES01	NAJWII
NULQER	NULQUH	NULROC	NULSAP	NUQMAQ	ODEDIN
OHIWAE	POFHAW	PUJKOV	RUZXUG	SABKEN	SAXCOK
SECWEE	SUZKUU	SUZNEH	TUCQOA	TUPTUU	TUPTUU01
TURJOF	VOQNEU	VURZIU	WAFKUN	XOKHUB	XOKHUB01
ZOBVAQ	ZUZLOX				

Protonated *p*-chloroheterocycles without a XB contact

BEMFEE	CIHCEC	CLPYCS	ELAGIK	KIBPET	LERFIZ
LIJFOA	LORNEM	MUWMAV	NURXAZ	OMOKUZ	OMOLIO
PANFUH	TEHVEK	TEHVEK01	TEHWAH	YUNTOT	

Protonated *o*-bromoheterocycles without a XB contact

GODHOZ	JADQAK	NOSLOZ	QQQGSA	SAQSEL	WUTJUQ
XEYXAB					

Protonated *m*-bromoheterocycles without a XB contact

AFULEV	ALAJAY01	BPYFSA	BPYFSB	DADGEX	DOQQOR02
DUSQAK	FIVDIX K	USTEY	LADBOJ	LAKWOL	LIBYIH
MUNSUM	NEWNIP	PUKDOP	QOZYOV	QQQGSD	RISFEH
SUYNUW	UXENIW02	YOKSAU			

Protonated *p*-bromoheterocycles without a XB contact

RISDOP	SIWVUR

Protonated *o*-iodoheterocycles without a XB contact

NAJWEE

Protonated *m*-iodoheterocycles without a XB contact

ALAJEC01	BEXPUR	BEXQAY	BIVMID	QQQGSG	VEGSEH
VEGTAE					

Protonated *p*-iodoheterocycles without a XB contact

SIWWAY	SIWWAY01

III. *N*-alkylated halogenoheterocycles

N-alkylated *o*-chloroheterocycles with a XB contact

AKEJUW	JETTOU	XEGYEN	XODWUK	APODOB

N-alkylated *m*-chloroheterocycles with a XB contact

KOHTUY

N-alkylated *p*-chloroheterocycles with a XB contact

-

N-alkylated *o*-bromoheterocycles with a XB contact

EDEQEL	NELGIV	WUKLIX

N-alkylated *m*-bromoheterocycles with a XB contact

BEYQIG	CIPYUY	EDEQEL	HIBMOX	IPEXAE	MEMVOQ
MEMWAD	QOXFOZ	QOXFUF	QULJEP	TUCTAN	WUJMAS
WUJMEW	WUJMIA	YARTIW	YARTOC	YARTUI Y	ARVOE
YARVUK	YARWAR	YOFLOX	YOFLUD	ZADNEX	OPUNUL

N-alkylated *p*-bromoheterocycles with a XB contact

BRCDOP

N-alkylated *o*-iodoheterocycles with a XB contact

EDEQOV	QEJTUX	YORZIR

N-alkylated *m*-iodoheterocycles with a XB contact

AQUROV	CIPYIM	GADKEF	HIHXII	HIHXOO	HIHXUU
HIHYAB	HIHYEF	HIHYIJ	HIHYOP	NOXLUJ	OJOFAX
OJOFEB	OPANUR	OPUJAN	OPUPOH	OPUQAU	ORATUY
ORAVAG	ORAVEK	PAQMUS	PAQNED	QAXWOD	QAXXAQ
QOXFIT	QOXGAM	TAWDON	TAWDUT	UJIJEF	UJIJIJ

UJIJOP	UJIJUV	UJIKAC	UJIKEG	WUJMOG	WUJMUM
WUJNAT	WUJNEX	WUWMUX	YAMYUH	YARSOB	YARSUH
YARTAO	YARTES	YARVAQ	YARVEU	YARVIY	YUDGUC
YUDHAJ	YUDHEN	YUDHOX	YUDHUD		

N-alkylated *p*-iodoheterocycles with a XB contact

ORAVIO VIPQAP

N-alkylated *o*-chloroheterocycles without a XB contact

AFOTIA	AFOTIA01	COXDAU	PIFFER	PIFFOB	PIFFOB01
QAXHUV	UYOCAN	XODVET	YORZUD		

N-alkylated *m*-chloroheterocycles without a XB contact

AKEJOQ AYASEA CIPXUX QOVNOH QOVNOH01 YORMAV

N-alkylated *p*-chloroheterocycles without a XB contact

ACROEY YACVIK

N-alkylated *o*-bromoheterocycles without a XB contact

AFOTOG EDEQIP EDEQUB

N-alkylated *m*-bromoheterocycles without a XB contact

DIJWIC	EDEQIP	EDEQOV	MEMVUW	NOGXEO	QAXWUJ
REWTAQ	REXYOK	SURROO	WUJLUL		

N-alkylated *p*-bromoheterocycles without a XB contact

AGUZUX

N-alkylated *o*-iodoheterocycles without a XB contact

-

N-alkylated *m*-iodoheterocycles without a XB contact

TAWDED TAWDIH YUDHIR

N-alkylated *p*-iodoheterocycles without a XB contact

WALYAN

Table S13. Halogen bond lengths (CSD data for structures measured at room temperature) for halogen bonds with N, O and I^- as acceptors and chloroheterocycles as donors.

Halogen bond acceptors							
Nitrogen		Oxygen				Iodide	
Refcode	Halogen bond length / **Å**	Refcode	Halogen bond length / **Å**	Refcode	Halogen bond length / **Å**	Refcode	Halogen bond length / **Å**
KEKFAJ	3,258	CXQULO01	3,254	TODFAV	3,113	CIGZUO	3,768
POMKUZ	3,25	DOCNEP	2,967	UBEKAP	3,122	CIHBIF	3,739
SAZZID17	3,29	FOVBUO	3,081	VADBAF	2,964	CIHCEC	3,733
SAZZID18	3,241	FUGWIO	3,269	VADBAF	3,102		
		HOTJIK	3	VADBAF	2,927		
		IDENUB	3,232	VADBAF	2,952		
		IKUCAU	2,876	VADBEJ	3,144		
		IKUCEY	2,963	VADBEJ	3,258		
		KEKFAJ	2,936	XEHNUU	3,269		
		NUQMAQ	3,24	XEHNUU	2,977		
		TODFAV	3,223				

Table S14. Halogen bond lengths (CSD data for structures measured at room temperature) for halogen bonds with N, O and I^- as acceptors and bromoheterocycles as donors.

Halogen bond acceptors							
Nitrogen		Oxygen				Iodide	
Refcode	Halogen bond length / **Å**	Refcode	Halogen bond length / **Å**	Refcode	Halogen bond length / **Å**	Refcode	Halogen bond length / **Å**
NAJWOO	3,155	AFULIZ	3,206	QOVLEV	3,02	BEYQIG	3,629
MUGWOC	3,293	BRCDOP	3,257	RISDAB	3,218	BEYQIG	3,52
MUGWOC	3,09	BRPYCH	3,182	RISDAB	3,341	CIGZOI	3,575
TUCTAN	3,131	FETYEK	3,094	SUYXIU	3,33	CIHBEB	3,589
		GALKUZ	3,346	TUCTAN	3,181	CIHBUR	3,648
		HGCBPO10	3,065	WOCROX	2,98	MOPJIL	3,596
		MISMAC	3,243	XACYIM	2,997		
		MUGWOC	3,265	XIMVAR	3,029		
		PEGTON	2,863	YOKSEY	3,083		
